# In utero origin of myelofibrosis presenting in adult monozygotic twins

**DOI:** 10.1038/s41591-022-01793-4

**Published:** 2022-05-30

**Authors:** Nikolaos Sousos, Máire Ní Leathlobhair, Christina Simoglou Karali, Eleni Louka, Nicola Bienz, Daniel Royston, Sally-Ann Clark, Angela Hamblin, Kieran Howard, Vikram Mathews, Biju George, Anindita Roy, Bethan Psaila, David C. Wedge, Adam J. Mead

**Affiliations:** 1 Medical Research Council (MRC) Molecular Haematology Unit, MRC Weatherall Institute of Molecular Medicine, National Institute for Health Research Biomedical Research Centre, University of Oxford, Oxford, UK; 2 Cancer and Haematology Centre, Churchill Hospital, Oxford University Hospitals NHS Foundation Trust, Oxford, UK; 3 Big Data Institute, Li Ka Shing Centre for Health Information and Discovery, University of Oxford, Oxford, UK; 4 Ludwig Institute for Cancer Research, University of Oxford, Oxford, UK; 5 Department of Microbiology, Moyne Institute of Preventive Medicine, School of Genetics and Microbiology, Trinity College Dublin, Dublin, Ireland; 6 Haematology Service, Wexham Park Hospital, Frimley Health NHS Foundation Trust, Slough, UK; 7 Department of Cellular Pathology, John Radcliffe Hospital, Oxford University Hospitals NHS Foundation Trust, Oxford, UK; 8 Flow Cytometry Facility, MRC Weatherall Institute of Molecular Medicine, University of Oxford, Oxford, UK; 9 National Institute for Health Research Biomedical Research Centre, University of Oxford, Oxford, UK; 10 Department of Haematology, Christian Medical College, Vellore, India; 11 Department of Paediatrics, MRC Weatherall Institute of Molecular Medicine, University of Oxford, Oxford, UK; 12 Manchester Cancer Research Centre, The University of Manchester, Manchester, UK

**Keywords:** Myeloproliferative disease, Myeloproliferative disease, Translational research, Cancer genomics

## Abstract

The latency between acquisition of an initiating somatic driver mutation by a single-cell and clinical presentation with cancer is largely unknown. We describe a remarkable case of monozygotic twins presenting with *CALR* mutation-positive myeloproliferative neoplasms (MPNs) (aged 37 and 38 years), with a clinical phenotype of primary myelofibrosis. The *CALR* mutation was absent in T cells and dermal fibroblasts, confirming somatic acquisition. Whole-genome sequencing lineage tracing revealed a common clonal origin of the *CALR-*mutant MPN clone, which occurred in utero followed by twin-to-twin transplacental transmission and subsequent similar disease latency. Index sorting and single-colony genotyping revealed phenotypic hematopoietic stem cells (HSCs) as the likely MPN-propagating cell. Furthermore, neonatal blood spot analysis confirmed in utero origin of the *JAK2V617F* mutation in a patient presenting with polycythemia vera (aged 34 years). These findings provide a unique window into the prolonged evolutionary dynamics of MPNs and fitness advantage exerted by MPN-associated driver mutations in HSCs.

## Abstract (web summary)

Lineage tracing analyses of cells from two monozygotic twins presenting with myelofibrosis in adulthood provide evidence of in utero transplacental transmission of the tumorigenic clone.

## Main

*BCR-ABL1*^−^ MPNs are a heterogeneous group of myeloid neoplasms characterized by the excessive production of mature myeloid cells, typically driven by somatic mutations affecting the MPL/JAK/STAT signaling pathway^1^. Primary myelofibrosis (PMF) is a subtype of MPN associated with bone marrow (BM) fibrosis, disease-associated symptoms, splenomegaly and abnormal blood counts. MPN-associated mutations are thought to originate in a single hematopoietic stem/progenitor cell (HSPC), driving a subsequent clonal expansion, eventually culminating in overt MPN after an unknown period of time. Emerging evidence from whole-genome sequencing (WGS) studies suggest that the latency between acquisition of an initiating driver mutation and disease presentation can be prolonged, even occurring over decades, with considerable tumor-to-tumor heterogeneity^2–4^. Studies of the increased risk of myeloid neoplasms (including MPNs) following radiation exposure raise the possibility of a short disease latency, suggesting that the peak risk occurs as early as 2–3 years following radiation exposure and falls sharply thereafter^5^. However, MPN-associated driver mutations are much more prevalent in individuals with normal blood counts than in disease^6^, so-called clonal hematopoiesis^7^, suggesting that the fitness advantage imposed by an MPN-driver mutation and disease latency might vary considerably from person to person.

Studies of the shared clonal origin of childhood leukemia in monochorionic twins have provided unique insights into the developmental timing, evolutionary trajectories and disease propagating cells in these diseases^8^. However, such cases were hitherto considered to be unique to blood cancers of childhood. We describe a remarkable case of monozygotic twins presenting with *CALR* mutation-positive^9,10^ PMF, which originated in utero and presented with overt disease after an almost identical and prolonged latency of over 35 years in both twins.

## Results

### Clinical findings

**Extended Data Fig. 1:**
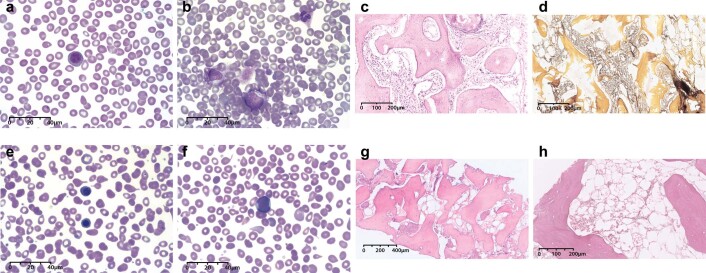
Blood film and bone marrow examination findings. **a,b**, Leucoerythroblastic blood film of twin A (May-Grunwald-Giemsa stain; 100x). **c**, Bone marrow trephine examination from twin A at diagnosis (hematoxylin and eosin stain). Reticulin stain (**d**) showed diffuse fiber network with scattered coarse fibers (WHO MF-3) (reticulin stain). **e,f**, Leucoerythroblastic blood film of twin B (May-Grunwald-Giemsa stain; 100x). **g**, Bone marrow trephine examination from twin B (hematoxylin and eosin stain). Reticulin stain (**h**) revealed evidence of patchy, irregular fibrosis and a hypocellular marrow (reticulin stain).

Twin A presented at the age of 37 years with symptomatic splenomegaly, measuring 21 cm on computed tomography imaging. Blood parameters were hemoglobin 138 g per liter, white cell count 5.9 ×10^9^ per liter and platelets 183 ×10^9^ per liter. Blood film examination revealed a leukoerythroblastic picture (Extended Data Fig. 1a,b). The BM showed typical features of PMF with distorted intertrabecular spaces, prominent dilated sinuses and evidence of heavily disrupted hematopoiesis with moderate numbers of highly atypical, small to medium-sized megakaryocytes forming small clusters, and World Health Organization MF-3 fibrosis (Extended Data Fig. 1c,d). Genetic testing revealed presence of a type 1 *CALR* mutation (NM_004343.4[CALR]:c.1092_1143del52 p.L367fs*46), a highly recurrent mutation in MPN^9,10^. Targeted next-generation sequencing revealed the presence of a frameshift *TET2* mutation (NM_001127208.2[TET2]:c.509del p.N170Tfs*13). The overall clinical presentation, genetic and morphological features were in keeping with a diagnosis of PMF. Due to disease-associated symptoms, twin A commenced treatment with the JAK1/2 inhibitor ruxolitinib with good clinical response. He remains on a stable dose of ruxolitinib (10 mg twice per day), with no disease-associated symptoms and reduction in spleen size to 17.6 cm on the most recent imaging. Blood parameters at the time of sampling for our studies were hemoglobin 138 g per liter, white cell count 7.3 × 10^9^ per liter and platelets 62 ×10^9^ per liter.

Twin A’s identical twin brother, twin B, presented 1 year later at the age of 38 years with marked splenomegaly, measuring 20 cm below the costal margin, and associated early satiety. Blood parameters were normal apart from borderline leukopenia (white cell count, 3.88 × 10^9^ per liter). Blood film examination revealed the presence of a leukoerythroblastic picture (Extended Data Fig. 1e,f). BM biopsy showed a hypocellular marrow with markedly abnormal bony trabeculae with irregular outlines and areas of notable thickening, alongside areas of increased reticulin fibers (Extended Data Fig. 1g,h). Genetic testing revealed presence of the same 52-bp deletion type 1 *CALR* mutation as seen in twin A. Targeted next-generation sequencing revealed no additional mutations in twin B. Taking clinical, genetic and morphological features into account, a diagnosis of PMF was made. He remains well, with no remarkable disease-associated symptoms, and to date, he has required no treatment.

There was no history of transfusion for either twin or other family history of MPN or other blood cancer. Information regarding whether the twins shared a monochorionic placenta was not available. The twins lived in the same household until the age of 25 years. Both are nonsmokers who do not drink alcohol, and they both have a normal body mass index and no autoimmune or other chronic medical conditions apart from MPN. They report no known exposure to ionizing radiation or occupational and chemical exposure.

### In utero origin of MPN

**Fig. 1:**
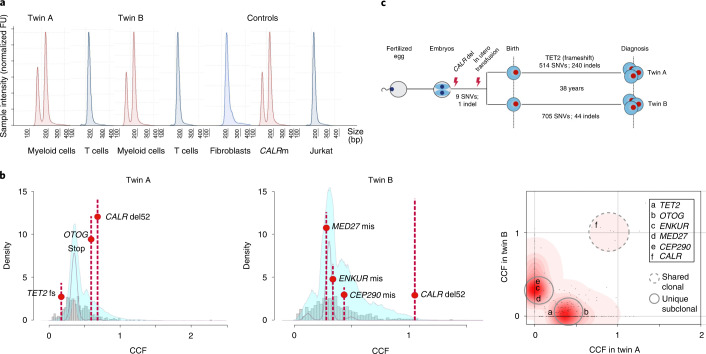
Genetic lineage tracing confirms a common in utero clonal origin of *CALR* mutation. **a**, Cell-lineage-specific *CALR* mutational analysis. T cell DNA electropherograms (blue) have a single peak at 207 bp, whereas DNA extracted from myeloid cells (red) shows two peaks, one at 207 bp and another one corresponding to the 52-bp deletion fragment (*CALRdel52bp* variant allele frequency (VAF) was calculated 28.4%, 32.6% and 43.7 for twin A, twin B and CALR type 1 myelofibrosis (*CALR*m) control, respectively). The absence of *CALR* 52-bp deletion in germline DNA was confirmed by analysis of dermal fibroblast DNA from twin B. DNA from a patient with known CALR type 1 myelofibrosis and DNA from Jurkat cells were used as positive and negative controls, respectively. Rearranged electropherograms from parallel experiments, all scaled to sample. FU, fluorescence units. **b**, Statistical modeling of the distribution of subclonal and clonal mutations by Dirichlet-process clustering. The histogram of mutations is represented with gray bars, with the fitted distribution as a gray line; 95% posterior confidence intervals for the fitted distribution are also shown (pale blue area). In the rightmost panel, a two-dimensional density plot shows Dirichlet clustering of the fraction of cancer cells (CCF) within each twin for all somatic mutations detected (black dots). Higher posterior probability of a cluster is indicated by increasing intensity of red. The cluster indicated around (1,1) corresponds to mutations present in all cells in both twins; clusters along the axes correspond to twin-specific clones. A subset of nonsilent coding mutations found in *TET2*, *OTOG*, *ENKUR*, *MED27, CEP290* and *CALR* are highlighted. **c**, Schematic representation of the shared in utero clonal origin and number of high-confidence shared variants and separate postnatal clonal evolution with total number of SNV and indels for each twin shown. fs, frameshift; mis, missense.

**Extended Data Fig. 2:**
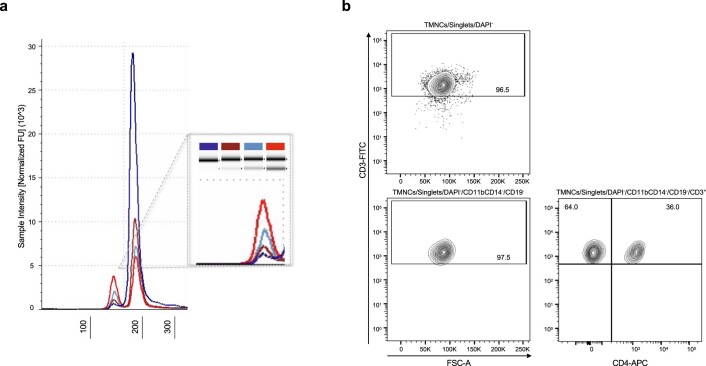
Cell-lineage tracing of the *CALR* mutation. **a**, Comparison of different sources of germline DNA for twin A. Automated electrophoresis for double-filtration CD3-enriched cell DNA is shown in dark blue, nail DNA in brown, single-filtration CD3-enriched cell DNA in light blue, while CD34-enriched cell DNA is shown in red. *CALRdel52bp* VAF was calculated 1.8%, 9.0%, 10.8%, and 37.1%, respectively. **b**, T cell purity assessment of the double-filtration CD3-enriched population by flow cytometry.

As the concordant disease in both twins might have occurred due to germline *CALR* mutation, we carried out mutational analysis of *CALR* in purified T cells (Extended Data Fig. 2) and myeloid cells for each twin and skin fibroblasts from twin B (Fig. 1a) by fragment analysis. The *CALR* mutation was present in myeloid cells, but only at a very low level in T cells from both twins. The *CALR* mutation was not detected in skin fibroblasts from twin B. These findings confirm that the *CALR* mutation was not present in the germline and was somatically acquired.

**Extended Data Fig. 3:**
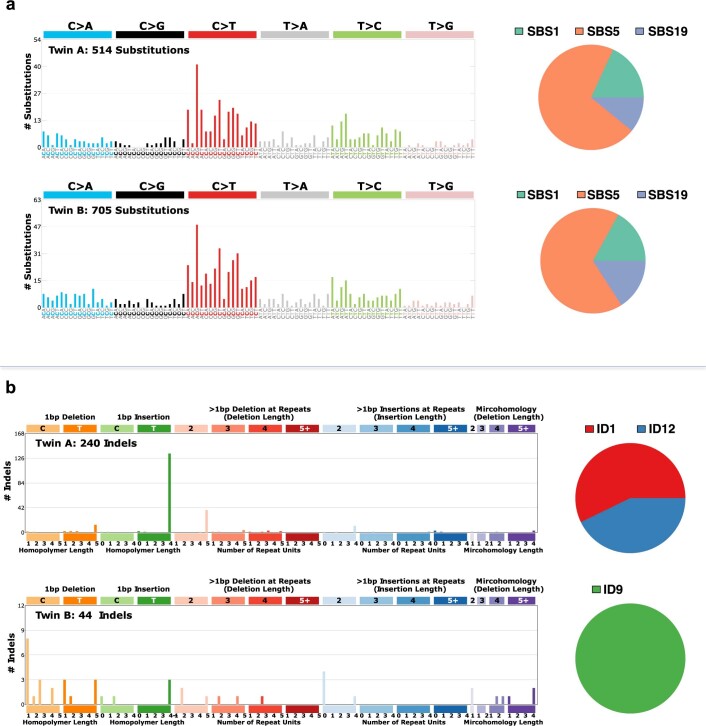
(a) Somatic point substitution and (b) indel spectra with corresponding mutational signatures for each of the twins. We identified 514 and 705 somatic single-nucleotide variants (SNVs), 240 and 44 somatic indels, and 5 structural variants unique to twin A and twin B, respectively. Analysis of the somatic point substitution signatures revealed shared signatures with near identical contributions in twin A and B. Indel signatures differed markedly between the twins; ID1 and ID12 accounted for respectively 57% and 43% of indels in twin A, while all indels in twin B were attributable to ID9.

We next reasoned that the somatic *CALR* mutation could either have been independently acquired by both twins or that the MPN was transmitted by intraplacental twin-to-twin transfer. To examine these two possibilities, we carried out lineage tracing of the MPN clone by WGS of peripheral blood granulocytes (derived from the MPN clone) and T cells (germline control) from both twins. If the MPN cells in both twins had a shared clonal origin, then we would expect to find a number of shared somatic variants in the granulocytes of both twins. We identified 514 and 705 somatic single-nucleotide variants (SNVs), 240 and 44 somatic indels and 5 structural variants (SVs) in twin A and twin B, respectively (Fig. 1b,c, Extended Data Fig. 3 and Supplementary Tables 1–3)^11–16^. The mean genome-wide substitution rate was 0.24 mutations per megabase, which represents a low mutational load, in line with the silent mutational landscape of MPNs^17^. In addition to CALR p.L367fs*46, which was the only shared mutation affecting a coding region, nine high-confidence SNVs were also detected in both twins, strongly supporting the in utero origin of the MPN (Fig. 1b,c and Supplementary Table 3).

**Extended Data Fig. 4:**
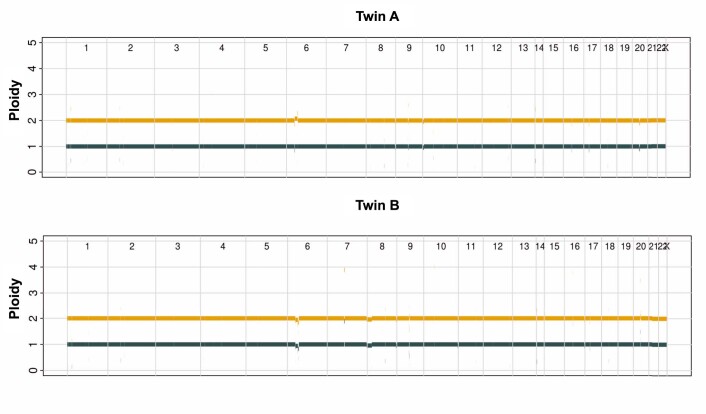
Genome-wide copy number profiles of twin A and twin B. Copy number profiles of twin A and twin B estimated using the Battenberg algorithm. The gold line corresponds to total copy number, the dark blue line corresponds to copy number of the minor allele. Average ploidy was estimated to be 1.98 and 2.03 for twin A and twin B, respectively. Aberrant cell fraction (cellularity) was estimated as 100% and 92% for twin A and twin B, respectively.

**Extended Data Fig. 5:**
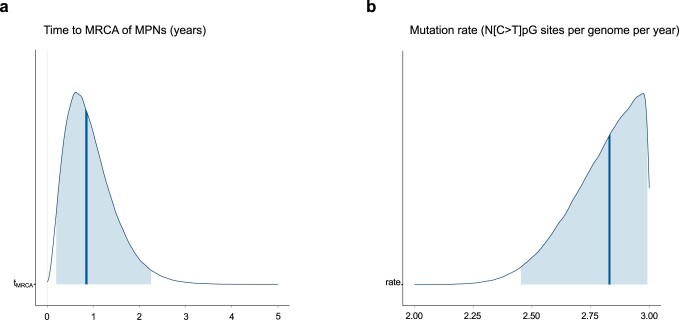
Posterior interval estimates from Markov chain Monte Carlo draws for (a) time to the most recent common ancestor (MRCA) of myeloproliferative neoplasms in twin A and twin B and (b) mutation rate. Posterior distributions with median (thick dark blue line) and 95% confidence interval (shaded blue area) for (**a**) MRCA timing and (**b**) mutation rate estimated using the numbers of somatic cytosine-to-thymine single-nucleotide variants (SNVs) at CpG sites (N[C>T]pG) in each twin. **a**. Time to MPN origin is estimated as years post-zygote (horizontal axis). **b**. Mutation rate is estimated as number of N[C>T]pG sites per genome per year (horizontal axis). N[C>T]pG mutation rate is truncated at an upper bound of 3 N[C>T]pG per genome per year; this bound was based on the highest N[C>T]pG rate reported in any Myeloid-MPN case in Gerstung et al.^3^.

No significant difference in overall somatic mutation burden was observed between the twins, although SNV and indel burdens differed when considered separately. Nonsilent somatic coding mutations exclusive to twin A included a frameshift indel not previously reported in *TET2*, an epigenetic regulator frequently mutated in hematological cancers, and a stop codon in *OTOG* associated with nonsyndromic hearing loss^18^, whereas twin B carried missense mutations in *MED27*, *ENKUR* and *CEP290* (Fig. 1b and Supplementary Table 4)^19^. The *ENKUR* variant is reported as a driver in the COSMIC (Catalogue of Somatic Mutations in Cancer) database^20^, but it is not associated with hematological cancer. Analysis of the mutational signatures by nonnegative matrix factorization^21^ revealed the presence of signatures 1, 5 and 19, all previously reported in MPNs, with near-identical contributions in twin A and twin B (Extended Data Fig. 3a)^3^. However, indel signatures differed markedly between the twins; ID1 and ID12 accounted for respectively 57% and 43% of indels in twin A, whereas all indels in twin B were attributable to ID9 (Extended Data Fig. 3b)^20^. This finding supports that the mutational processes were distinct, even though disease latency was almost identical in both twins. To assess clonal architecture, we clustered mutations based on cancer cell fraction (CCF), determined by adjusting the variant allele frequencies of SNVs for copy-number status and sample purity estimates (Fig. 1b and Extended Data Fig. 4)^22^. A number of studies have used mutational processes as a molecular clock to carry out chronological time estimates of disease latency in cancer^3,23^. The current study provided a unique opportunity to orthogonally validate such an approach for a blood cancer with a relatively silent mutational landscape such as MPN. We derived an estimate for the time to the most recent common ancestor of MPN cells in both twins (that is, the time of twin-to-twin transmission of the *CALR*-mutant clone) from WGS-identified SNV data arising from a clock-like mutational process. Time to the most recent common ancestor was consistent with an in utero origin of MPN (Extended Data Fig. 5 and Supplementary Table 5).

### Phenotypic and genetic analysis of HSPCs

**Fig. 2:**
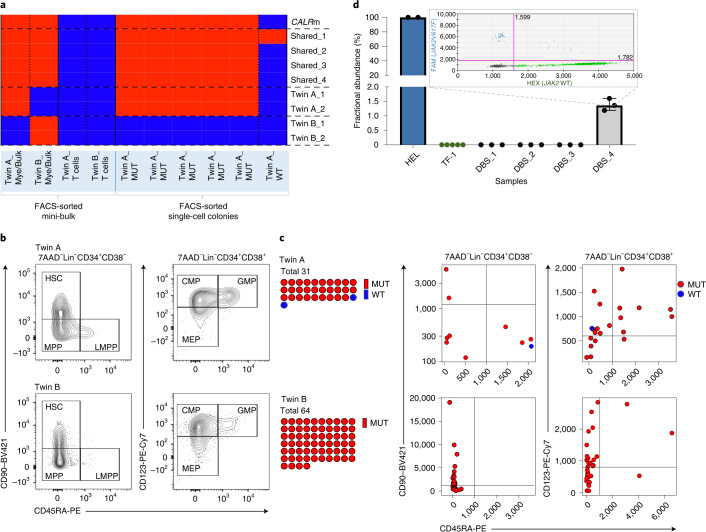
Single-cell-derived colony *CALR* genotyping and neonatal blood spot analysis. **a**, Orthogonal validation of the WGS results at the single-cell level. Somatic variants shared between the twins and somatic variants unique for twin A or twin B were assessed in DNA from fluorescence-activated cell sorting (FACS)-sorted single-HSPC colonies (from twin A) or mini-bulk populations using Sanger sequencing. The samples with presence of the studied variant are shown in red, whereas absence of the variant is shown in blue. Details of the tested variants are shown in Supplementary Table 6. **b**, Flow cytometry profiles of HSPCs for each of the twins. **c**, *CALR* genotyping of single-HSPC-derived colonies, integrated with index sorting data. **d**, Results of ddPCR analysis for the detection and quantification of *JAK2V617F* in DNA extracted from neonatal dried blood spots from patients diagnosed with *JAK2*-mutant myeloproliferative neoplasms as adults. In one out of three patients studied (DBS_4) *JAK2V617F* was detected in neonatal blood with a fractional abundance of 1.38% (three technical replicates in one experiment, independently validated by nested PCR). FAM channel-positive events on the *y* axis correspond to *JAK2V617F* positivity, and HEX channel-positive events on the *x* axis correspond to *JAK2* WT events. *JAK2V617F*-positive DNA from HEL cells, and JAK2 wild-type DNA from TF-1 cells and a dried blood spot from a patient with systemic mastocytosis (DBS_1), were used as positive and negative controls, respectively. All results were independently validated by a nested PCR method. Bars and error bars show median and 95% confidence interval, respectively. Relevant clinical information for the patients studied is provided in Supplementary Table 7. CMP, common myeloid progenitor (Lin^−^CD34^+^CD38^+^CD123^+^CD45RA^−^); GMP, granulocyte macrophage progenitor (Lin^−^CD34^+^CD38^+^CD123^+^CD45RA^+^); HSC, Lin^−^CD34^+^CD38^−^CD90^+^CD45RA^−^; LMPP, lymphoid-primed multipotent progenitor (Lin^−^CD34^+^CD38^−^CD90^−^CD45RA^+^); MEP, megakaryocyte-erythroid progenitor (Lin^−^CD34^+^CD38^+^CD123^−^CD45RA^−^); MPP, multipotent progenitor (Lin^−^CD34^+^CD38^−^CD90^−^CD45RA^−^); MUT, mutated; Mye, myeloid; PE-Cy7, PE-cyanine7; WT, wild-type.

Studies in monozygotic twins have provided unique insights into the cancer-propagating cells in childhood leukemia^8^. Some evidence suggests that the cell of origin in MPNs is the phenotypic HSC^1^. Twin-to-twin transfusion of the MPN clone provided an opportunity to study the cell-of-origin in MPN. If the *CALR* mutation originally occurred in an HSC, then the *CALR* mutation should be present in HSCs from both twins. We performed index sorting of single Lin^−^CD34^+^ HSPCs into individual wells for colony-forming assay, recording the surface phenotype for CD90, CD45RA and CD123. Resulting colonies were picked, and DNA was whole-genome amplified. We first confirmed that a number of the shared somatic mutations present in both twins were also present in the colony-derived material for twin A and bulk material for both twins. As expected, for the four shared mutations analyzed, they were all present in both twins, whereas somatic mutations exclusive to each twin were only detected in twin A and twin B, respectively (Fig. 2a and Supplementary Table 6). The *CALR* mutation was present in all colonies from twin A with shared mutations. One wild-type *CALR* colony from twin A showed presence of one of the shared mutations, supporting that this mutation occurred before the *CALR* mutation.

Analysis of 95 colonies demonstrated high clonal dominance of *CALR-*mutant cells, with 29 of 31 colonies in twin A and 64 of 64 in twin B showing presence of the *CALR* mutation. The two *CALR* mutation-negative colonies in twin A likely represent residual nonclonal hematopoiesis, as they were negative for additional shared variants between the twins. The index sorting data (Fig. 2b) allowed colonies to be traced back to canonical HSPC hierarchies, revealing presence of *CALR* mutation-positive phenotypic HSCs (Lin^−^CD34^+^CD90^+^CD45RA^−^) in both twins (Fig. 2c), supporting that the HSC is the propagating cell for the MPN clone.

### Neonatal blood spot analysis in *JAK2V617F*-mutant MPN

To determine whether *JAK2* mutation-positive MPN might also originate in utero yet present clinically decades later, we carried out *JAK2V617F* mutation analysis of neonatal blood spots stored from patients subsequently diagnosed with MPN as adults. We were able to identify three *JAK2V617F* mutation-positive MPN patients whose Guthrie cards were available (Supplementary Table 7). *JAK2V617F* mutation detection and quantification was performed by droplet digital polymerase chain reaction (ddPCR). The analysis revealed presence of the *JAK2V617F* mutation in the neonatal blood spot of one of the three patients studied, with a variant allele frequency of 1.38% (Fig. 2d). This patient presented with *JAK2V617F* mutation-positive polycythemia vera at age 34 years. These findings support that an in utero origin of the MPN clone, presenting after a prolonged disease latency, may be a widespread phenomenon in MPN, warranting further studies.

## Discussion

Emerging evidence from WGS studies of solid tumors supports that the latency between acquisition of an initiating driver mutation and presentation with overt cancer can be prolonged, with important implications for early diagnosis and intervention^2,3^. However, these studies are based on multiple-site biopsies of tumors^2^ or analysis of tumors with high genetic heterogeneity and require certain assumptions to be made about linearity of mutational processes in tumors^2,3^. Transplacental transmission of hematological neoplasms between twins has provided a unique opportunity to study disease latency and genetic evolution of blood cancers but was hitherto considered to be a phenomenon occurring exclusively during childhood, including pediatric MPN^24,25^. We describe transplacental transmission of a *CALR*-mutant clone between a pair of monozygotic twins, which resulted in the development of overt myelofibrosis after a period of almost four decades. This remarkable case provides definitive evidence of a very early developmental origin of *CALR*-mutant blood cancer, presenting in adults as overt disease decades later. Single-colony-derived WGS data support that a prolonged disease latency might also be a widespread phenomenon in *JAK2V617F-*mutated MPNs^4,26^ and that *JAK2* mutations might even occur in utero in some patients^26^. We also carried out neonatal blood spot analysis, which confirmed the in utero origin of *JAK2V617F* mutation in a patient presenting with polycythemia vera at an age of 34 years. Our findings definitively confirm long disease latency and in utero origin can occur for both *JAK2* and *CALR* mutation-driven MPN. Furthermore, the unique ability to follow the same clone in the two siblings revealed a strikingly similar and prolonged disease latency for *CALR*-mutation-positive MPN. This finding is compatible with the fitness advantage exerted by the clone being established early and not being contingent upon subsequent clonal evolution. However, it also remains possible that the *TET2* or other putative driver mutations identified in the twins collectively resulted in the same latency in the two twins. Moreover, the similar disease latency in both twins suggests that its primary determinants are cell-intrinsic rather than extrinsic factors, although environmental exposures in the twins would be largely shared in childhood. Notably, the similar disease latency might reflect the identical germline genetics, which may influence HSC response to MPN-associated mutations^27^. Our study also provided additional evidence that the phenotypic HSC is the key propagating population for MPN^1^. Of note, overexpression of fetal-associated genes in HSPCs has been demonstrated to promote the development of MPN in model systems, suggesting that fetal hematopoiesis might be permissive for the acquisition of MPN-associated mutations^28^.

A Danish cohort study previously reported concordance for MPN in 15% of monozygotic twins;^29^ although the authors concluded that this might reflect genetic predisposition to MPN, it may be that these cases were due to an in utero origin and transplacental transmission, as concordant cases were not observed in dizygotic twins. Furthermore, concordance of clonal hematopoiesis with identical somatic mutations in elderly twins has also been reported, suggesting that clones that arise in utero might even persist lifelong until old age without causing disease^30,31^. Whether an in utero versus postnatal acquisition of an MPN-driver mutation might influence the subsequent phenotype is unknown. Larger studies of blood cancers in monozygotic twins and of neonatal blood spots in sporadic MPNs are warranted, as this will provide a unique window into the evolution of MPNs and fitness advantage exerted by MPN-associated driver mutations. The long disease latency in combination with a better understanding of the evolutionary dynamics in MPN and availability of robust polygenic risk scores^27^ might open up opportunities for early intervention. Although currently the only curative treatment for MPN remains BM transplantation, there is accumulating evidence that certain treatments such as interferon or targeted inhibitors can induce molecular remissions, and it is also possible that the fetal origin in some cases of MPN might also present unanticipated therapeutic vulnerabilities, paving the way for disease-modifying early intervention strategies in MPN^32^.

## Methods

### Cell isolation

Granulocyte enrichment and isolation was carried out by Ficoll density gradient centrifugation (Ficoll-Paque PLUS; GE Healthcare Bio-Sciences AB), and subsequent red blood cell lysis of the pellet, using the QIAGEN RBC Lysis Solution (QIAGEN). Granulocytes were also sorted in mini-bulk by fluorescence-activated cell sorting (FACS) and whole-genome amplified, as described below. The antibody panel that was used is shown in Supplementary Table 8 and granulocytes were defined as 7AAD^−^CD3^−^CD71^−^CD19^−^CD11bCD14^+^CD33^+^. HSPC and T cell enrichment was done with magnetic-activated cell sorting of total mononuclear cells, using the CD34 MicroBead Kit UltraPure, human and CD3 MicroBeads (double-columnenrichment to increase purity of T cells) human kits, respectively (Miltenyi Biotec). The purity of the final T cell (CD3-enriched) population was assessed by flow cytometry analysis (Supplementary Fig. 1). The antibody panel that was used is shown in Supplementary Table 8, and T cells were defined as DAPI^−^CD11bCD14^−^CD19^−^CD3^+^. T cell purity was greater than 96% (Extended Data Fig. 2b).

Nail DNA from nail clippings of twin A and neonatal dried blood spot DNA were extracted using the QIAamp DNA Micro kit (QIAGEN) according to the manufacturer’s instructions.

Dermal fibroblast cell cultures were set up following a skin biopsy from the proximal thigh in twin B. Cells were cultured in Gibco AmnioMAX C-100 Complete Medium (Thermo Fisher Scientific) and Chang medium D (FUJIFILM Irvine Scientific) without additives. They were cultured at 37 °C, 5% CO_2_, and subcultured when confluent. At 22 days in culture, cells were removed from the subcultures in Chang medium using trypsin, and DNA was extracted. DNA from the cells in the AmnioMAX medium was extracted at day 27.

### Cell lines

A Jurkat cell line (CVCL_0065) was used as the *CALRdel52bp*-negative control. TF-1 (CVCL_0559) and HEL (CVCL_0001) cell lines were used as *JAK2V617F* positive and negative controls, respectively. All cell lines were purchased from American Type Culture Collection (catalog numbers TIB-152, CRL-2003, and TIB-180 for Jurkat, TF-1 and HEL, respectively). Cells were cultured according to American Type Culture Collection recommendations, and DNA was extracted using the DNeasy Blood & Tissue kit (QIAGEN).

### Microscopy

Peripheral blood microscopy was done using the Olympus BX60 microscope with the Olympus UPlanFl 40×/0.75 Infinity/0.17 and Olympus UPlanSApo 100×/1.40 Oil Immersion Infinity/0.17/FN26.5 objectives (Olympus). Imaging was performed with the INFINITY3S-1UR camera and use of the Infinity ANALYZE software, release 6.5 (Lumenera). Tissue microscopy was performed using a Nikon Eclipse E400 microscope (40×/20× objectives) (Nikon) on routinely prepared 4-μm sections cut from formalin-fixed paraffin-embedded blocks. Microscope fields were selected from whole-slide scanned images using the NanoZoomer 2.0-HT scanner in 40× mode and NDP.view2 viewer software v2.9.25 (Hamamatsu Photonics). Image analysis was done using Adobe Photoshop v21.2 (Adobe) and was limited to white balance of complete image and addition of scale bars.

### Flow cytometry and cell sorting

Flow cytometry and cell sorting was performed using a BD FACSAria Fusion Cell Sorter (Becton, Dickinson and Company) with FACSDIVA software v8.0.1. Sorting of single cells into 96-well plates was performed using the automated cell deposition unit. Verification of the single-cell sorting mode was established by sorting single fluorescent beads (Alignflow Flow Cytometry Alignment Beads; Thermo Fisher Scientific) into a flat-bottomed 96-well tissue culture plate. A fluorescence microscope was used to visualize the beads to verify that no wells contained more than one fluorescent bead and that they were centrally positioned in the wells. Further flow cytometry analysis was performed using FlowJo software v10.7 (Becton, Dickinson and Company).

Cryopreserved peripheral blood mononuclear cells were thawed and processed for flow cytometry analysis and cell sorting as previously described^33^. Staining panel details for granulocyte, T cell and HSPC analysis and sorting are shown in Supplementary Table 8, and the gating strategy is provided in Supplementary Fig. 1.

### Single-cell cloning assay

Single HSPCs (7AAD^−^, Lin^−^, CD34^+^) were sorted into 96-well plates with 50 μl of MethoCult H4435 Enriched (STEMCELL Technologies). The mean fluorescence intensities of CD34, CD38, CD90, CD45RA and CD123 were also recorded for each individual HSPC sorted using the index sorting feature. Colony output was assessed on day 14 under direct light microscopy, and individual colonies were picked. All colonies were flash-frozen and stored at −80^o^C for subsequent whole-genome amplification. Index-sorting analysis was performed by in-house bioinformatics pipelines using R studio v3.6.3.

### Whole-genome amplification

Whole-genome amplification for each sorted population was performed using the REPLI-g Mini/Midi Kits (QIAGEN).

### PCR

#### Twin studies

PCR target regions and corresponding primer set sequences for the lineage-specific *CALR* genotyping, and validation of the targeted sequencing and WGS results at the single-cell level are shown in Supplementary Tables 6 and 9. All PCRs were performed using the KAPA2G Robust HotStart ReadyMix PCR Kit (Merck) with the following conditions: 12.5 μl KAPA2G Robust HotStart ReadyMix (2×), primer set at 0.5 μM each, template DNA as required and PCR-grade water up to 25 μl; and the following cycling protocol: initial denaturation for 3 min at 95 °C, 35 cycles of denaturation for 15 s at 95 °C, annealing for 15 s at 60 °C, extension for 15 s at 72 °C and final extension for 3 min at 72 °C. All PCR amplicons were purified using either Agencourt AMPure XP Magnetic Beads (Beckman Coulter) or the Monarch DNA Gel Extraction Kit (New England Biolabs).

#### Neonatal dried blood spot studies

Neonatal dried blood spot mutational analysis was performed with use of ddPCR in a two-step PCR. *JAK2* exon 14 (chr9(GRCh38):5073730–5073813) was amplified in the first reaction, and the amplicon was then tested for presence of the NM_004972.3[JAK2]:c.1849G > T p.V617F mutation using a ddPCR assay. The same primers were used for both reactions. Primer and probe details are provided in Supplementary Table 9. The first round of PCR was performed using the KAPA HiFi HotStart ReadyMix PCR Kit (Merck) with the following conditions: 10 μl KAPA HiFi HotStart ReadyMix (2×), primers at 0.3 μM, 3 μl template DNA and PCR-grade water up to 20 μl; and the following cycling protocol: initial denaturation for 3 min at 95 °C, 20 cycles of denaturation for 20 s at 98 °C, annealing for 15 s at 62.5 °C, extension for 30 seconds at 72 °C and final extension for 1 min at 72 °C. ddPCR reactions were performed using the Bio-Rad QX200 AutoDG Droplet Digital PCR System (Bio-Rad Laboratories). Sample preparation was done with Bio-Rad’s Supermix for Probes (no dUTP) with the following conditions: 11 μl of the supermix (2×), primers at 900 nM each, probes at 250 nM each, 5 ng preamplified DNA template in a 1:100,000 dilution and PCR-grade water up to 22 μl. The amplification was performed using the Bio-Rad C1000 Touch Thermal Cycler with the following cycling protocol: initial enzyme activation for 10 min at 95 °C, 43 cycles of denaturation for 30 s at 94 °C, annealing/extension for 1 min at 57 °C and enzyme deactivation for 10 min at 98 °C (ramp rate 2 °C/s at all steps). DNA extracted from the Guthrie card of a patient with *JAK2* wild-type systemic mastocytosis (DBS_1) and TF-1 cells were used as negative controls, whereas HEL cell DNA was used as the positive control, with multiple replicates per run. Presence of *JAK2V617F* in one of the samples was independently validated by nested PCR. ddPCR data analysis was done using QX Manager Software, Standard Edition, v1.2 (Bio-Rad Laboratories).

### Gel electrophoresis

Initial assessment of DNA fragment size was performed using gel electrophoresis on a 3% ethidium bromide-containing (10^−4^ g per liter) agarose gel in TAE buffer (Tris acetate-EDTA, Tris acetate 40 mM and 1 mM EDTA, pH 8.3) at 150 mV. Approximate DNA fragment size was determined using a 50-bp ladder (PCRBIO Ladder III, PCR Biosystems).

### Automated electrophoresis

Separation, identification and quantitation of the type 1 *CALR* mutation within the different cell populations examined was performed with use of the Agilent Fragment Analyzer automated fluorescence-based capillary electrophoresis and the DNF-905 dsDNA kit of 35–500 bp sizing range and/or the Agilent 2200 TapeStation automated electrophoresis system, using the D1000 ScreenTape System and the A.0202 SR1 software version (Agilent Technologies).

### Sanger DNA sequencing

Sanger DNA sequencing was performed using the Applied Biosystems 3730 DNA Analyzer (Thermo Fisher Scientific) with use of the BigDye Terminator v3.1 chemistry. Results were visualized and annotated using the SnapGene Viewer software, v5.3 (GSL Biotech) and the Heatmapper visualizer^34^.

### Next-generation sequencing

Next-generation sequencing of granulocyte DNA from each twin was performed using an adapted method based on the previously described method by Silveira et al.^35^. The genes and transcript accessions covered by the panel are shown in Supplementary Table 10.

Notably, the targeted panel analysis for twin A showed presence of two TET2 mutations at chr4(GRCh38):105234449 (NM_001127208.2[TET2]:c.509del p.N170TfsTer13) and chr4(GRCh38):105259633 (NM_001127208.2[TET2]:c.3818 G > A p.C1273Y), with VAFs 9.21% and 8.63%, respectively. Both mutations were validated by Sanger sequencing both in bulk populations and in single-HSPC colonies (data not shown). The frameshift mutation was also detected in the WGS data, but the substitution was supported from a single caller only and therefore did not meet the variant calling criteria set in the study (as described below). This is a missense mutation that is not annotated in ClinVar (https://www.ncbi.nlm.nih.gov/clinvar/) database, but it appears in COSMIC as pathogenic (https://cancer.sanger.ac.uk/cosmic/mutation/overview?id=145018270), with a CADD score of 27.2 (ref. ^36^), whereas the SIFT^37^ and PolyPhen^38^ tools predict its effect to be deleterious and probably damaging, respectively. However, because it was not included in the high-confidence set for WGS data, it was excluded from the downstream analysis.

### WGS

Paired-end sequencing of matched tumor and germline DNA samples for each twin was performed by Novogene, and 350-bp insert libraries were prepared for all samples and run on the NovaSeq 6000 System (Illumina) to generate 150-bp reads, aiming for 30× sequencing coverage.

### Whole-genome data processing and alignment

Whole-genome sequence FASTQ files were processed and aligned as follows. The quality of short insert paired-end reads was assessed by FASTQC v0.11.9 (https://www.bioinformatics.babraham.ac.uk/projects/fastqc/). Sequencing reads were aligned to the human reference genome (GRCh38.p13) using the BWA-MEM^39^ (v0.7.17) aligner in default mode. Duplicates were marked using Picard tools v2.3.0 (https://broadinstitute.github.io/picard/); indels were realigned and base quality scores were recalibrated according to Genome Analysis Toolkit best practice to generate analysis-ready BAM files. The resulting sequencing coverage ranged from 36.5× to 42.4×.

### Variant calling

We reported somatic SNVs and indels that overlapped across three different software packages: MuTect2 (Genome Analysis Toolkit v4.1.2.0), strelka2 (v2.8.4) and Octopus (v0.6.3-beta)^11–13^. Random forest filtering was applied to both somatic and germline calls generated using the Octopus algorithm^13^. Somatic variants notated as ‘somatic’ and ‘PASS’ in the VCF files generated by Octopus and strelka2 and “PASS” in the VCF files generated by MuTect2 were retained and constitute the high-confidence call set. Variants were classified as shared or unique based on overlap between the twin call sets.

### Copy-number estimation

The Battenberg package (v.2.2.8; https://github.com/Wedge-lab/battenberg) was implemented in R 3.3.0 to estimate tumor cellularity and detect clonal and subclonal copy-number aberrations (CNAs) for each sample^40^ (Extended Data Fig. 4). An approach similar to the R-only WGS pipeline described at https://github.com/Wedge-lab/battenberg/blob/master/inst/example/battenberg_wgs.R was used, including the preprocessing, allele-counting and phasing steps and using genome assembly hg38. Replication timing correction was not implemented and SV breakpoint information was not included. Default parameters were used, but the PHASING_GAMMA parameter was increased to 2 to correct for overfitting. To run Battenberg in a way that was compatible with the hg38 genome build, Impute2 inputs were converted to hg37 using liftOver^41^. Heterozygous SNP positions were then phased using Beagle 5 (ref. ^41^) with use of the 1000 Genomes genotypes as a reference panel and the output was converted to hg38. The ‘ntot’ values for nearly all CNAs identified by Battenberg were approximately 2, suggesting that these were likely to be homozygous SNPs rather than somatic CNAs. To confirm whether these were heterozygous SNPs in the normal, distributions of allele frequencies of all SNPs within these CNAs in both the tumor and normal samples were inspected; copy-number segments were excluded where these distributions did not correspond to ‘real’ somatic CNAs.

### Mutation clustering

Copy-number changes called by Battenberg (‘Copy-number estimation’) along with VAFs for each mutation were used to calculate CCF and prepared as input for DPClust^22^, a Bayesian Dirichlet algorithm available at https://github.com/Wedge-lab/dpclust. Before running DPClust, we used the vafCorrect realignment tool (https://github.com/cancerit/vafCorrect) to calculate VAF values for all somatic variant calls (‘Variant calling’) from tumor and germline BAM files directly^42^. The vafCorrect read mapping and base quality thresholds were set to 30. Variants with VAF > 0.05 in either germline sample or VAF < 0.1 in a tumor sample were flagged and removed prior to mutation clustering.

For each twin, we inferred the number of subclones and the fraction of tumor cells within each subclone using DPClust (v2.2.2) implemented in R 3.4.0 to cluster mutations according to their CCF. A pipeline similar to that described at https://github.com/Wedge-lab/dpclust/blob/master/inst/example/dpclust_pipeline.R was used with 1,000 iterations to run the Markov chain Monte Carlo chain (no.iters) and discarding 200 iterations as burn-in (no.iters.burn.in or burnin). Clusters that included less than 1% total mutations were removed.

DPClust was also used to cluster mutations according to their CCFs across samples from both twins simultaneously (Supplementary Table 11). DPClust input files for multidimensional DPClust contained all mutations across both samples. Variant sites with total coverage below 20 reads or with a mutation multiplicity estimate (no.chrs.bearing.mut) of zero in either sample were flagged and removed prior to multidimensional clustering. Depth at variant sites was determined using vafCorrect.

To perform statistical tests on individual mutations and establish confidence intervals for CCF estimates, the binomial probability function pbinom was used in R. This returns the likelihood pbinom(*x*, *y*, z) of observing *x* or fewer mutant reads from a depth of *y* given an expected VAF of *z*.

### SV analysis

SVs were called using Manta^14^ (v1.6.0; https://github.com/Illumina/manta) and LUMPY^15^ (v0.2.13; https://github.com/arq5x/lumpy-sv). The LUMPY express wrapper was used to run LUMPY jointly on tumor-normal pairs. SV breakpoints identified by the algorithm were then genotyped using SVTyper^16^ (v0.7.1; https://github.com/hall-lab/svtyper), run as a command line python script, and variants with any alt (AO) evidence in the normal were removed. In Manta, tumor-normal analysis workflows were configured using the configManta.py script with default settings; SVs were then filtered to include only calls marked as “PASS”. For each twin, high-confidence somatic calls were derived by requiring either both callers to support an SV or one caller with additional support from a nearby CNV changepoint. In comparing alignments between Manta and LUMPY, a bidirectional ‘slop’ of 200 bp was used to account for possible imprecision in some reads. SVs were annotated using the online AnnotSV interface v3.0.2 (https://lbgi.fr/AnnotSV/runjob) using default settings^43^.

### Extraction of mutational signatures

Single-base substitution, doublet-base substitution and indel signatures were extracted by nonnegative matrix factorization, as implemented in SigProfilerMatrixGenerator v1.1.23 (https://github.com/AlexandrovLab/SigProfilerMatrixGenerator) using Python 3.8.3 (ref. ^21^). SigProfilerExtractor (v1.1.0; https://github.com/AlexandrovLab/SigProfilerMatrixExtractor) was used to determine the proportion of mutations in each sample attributable to specific mutational signatures within the COSMIC database of signatures (COSMIC v3.1, available at https://cancer.sanger.ac.uk/cosmic/signatures).

### Putative driver mutation analyses

To identify nonsilent coding mutations of interest, we considered 82 genes with relevant biological evidence^44–46^ (Supplementary Table 12) as well as genes from the COSMIC Cancer Gene Census list (August 2020, http://cancer.sanger.ac.uk/census). Somatic variants were annotated using Ensembl Variant Effect Predictor release 103 (ref. ^19^). Variants were considered pathogenic if their clinical significance was pathogenic, their impact was high or their CADD score was ≥30 (ref. ^36^).

### Bayesian estimates of myeloproliferative neoplasm developmental timing

An estimate for the time to the most recent common ancestor (MRCA) of MPN cells in both twins, *t*_MRCA_ (that is the time at which in utero transfusion took place), was derived using a Poisson Bayesian model. Known data used in the model included the ages of both twins at sampling (39 years 11 months 26 days for twin A, 38 years 5 months 14 days for twin B) and mutation count data generated in this study. Using the pigeonhole principle, it was inferred that mutations from clusters 1 and 2 with CCF > 0.5 occurred on the same individual cell lineages in twin B and twin A, respectively (Supplementary Table 11). Mutations shared by the twins and occurring on the same cell lineage were assigned to cluster 3 (Supplementary Table 11). An initial model was built using these lineage-restricted counts (Supplementary Table 5). The mutation rate and the number of SNVs (somatic variation data obtained from twin A and twin B) was modeled as mutations per genome per year. The prior on the mutation rate was exponential with mean of 10.6 clonal substitutions per genome per year; this estimate was based on reported clonal point mutation counts and donor ages in patients with MPN within the Pan-Cancer Analysis of Whole Genomes (PCAWG; see Gerstung et al., clonal counts from source data for Fig. 2 and donor ages Extended Data Fig. 8b)^3^. Pre-MRCA mutation count data (SNVs shared by the twins and occurring on the same cell lineage) had observed values of 7. Post-MRCA mutation count data (lineage-restricted SNVs unique to each twin) had observed values of 91 and 90 for twin A and twin B, respectively. An upper bound on the rate parameter of the Poisson process (mutations per genome per year) was set based on the highest reported clonal substitution rate in a patient with MPN in PCAWG.

Results presented in the main text derive from a model using C>T somatic mutations in a NpCpG context (N[C>T]pG) (Supplementary Table 5a), as these mutations are hypothesized to arise in a clock-like manner as a result of spontaneous deamination of 5-methylcytosine and can be used been to estimate MRCA timing in cancers^3,23^. N[C>T]pG mutation counts were extracted using the R/Bioconductor package MutationalPatterns (v1.12.0) implemented in R 3.6.0 (ref. ^47^). An important assumption of this model is that the N[C>T]pG mutation rate has remained constant over time in the MPN lineages in both twins and that MPN lineages in twin A and B shared a common ancestor in the past. A further limitation of this model is that N[C>T]pG counts estimated from bulk data will not necessarily correspond to those arising on a single-cell lineage; 89 and 114 C>T somatic mutations in an NpCpG context were identified uniquely in twin A and twin B, respectively. Two shared N[C>T]pG somatic mutations were identified from the set of all shared mutations listed in Supplementary Table 3. Mutation counts were based on vafCorrect genotyping data after filtering (‘Mutation clustering’). The prior on the mutation rate was exponential with a mean of 1.71 N[C>T]pG substitutions per genome per year; the mean estimated rate was based on reported N[C>T]pG counts and donor ages in patients with MPN within PCAWG (see Gerstung et al., Extended Data Fig. 8b)^3^. 40 weeks of gestation time was also added to donor ages when generating mutation rate estimates from PCAWG data. An upper bound on the rate parameter of the Poisson process (mutations per genome per year) was set based on the highest reported N[C>T]pG rate in a patient with myeloid-MPN in PCAWG. The prior on *t*_MRCA_ (that is, the span of time after zygote over which shared N[C>T]pG mutations arose) was defined as uniform between the time of fertilization and the age of earliest presentation in the twins. *t*[1] and *t*[2] are time intervals during which subsequent (post-*t*MRCA) N[C>T]pG mutations arose in twin A and twin B, respectively. The prior on these intervals was defined as uniform between *t*_MRCA_ and the time of sampling (approximately 40 years of age).

Mutation counts were modeled using a Poisson likelihood with rates given by the product of the mutation rate and the relevant time interval (that is, the time interval from fertilization up to *t*_MRCA_ for pre-MRCA count data and the time interval between *t*_MRCA_ up to the time of sampling for post-MRCA count data). The No-U-Turn sampler, implemented in Stan using the rstan package (https://CRAN.R-project.org/package=rstan; v2.21.2, GitRev: 2e1f913d3ca3), was used to draw samples from the model’s posterior distribution through Markov chain Monte Carlo sampling using five chains, each with 100,000 iterations, of which the first 20,000 are warmup^48^. Log marginal likelihoods and error measures for marginal likelihood estimates were computed via bridge sampling using the ‘bridgesampling’ package in R^49^. Stan model outputs, likelihood estimates and error measures are reported in Supplementary Table 5. A model using lineage-restricted or clonal N[C>T]pG mutations could not be implemented, as the mutation count data were too sparse.

### Informed consent

All procedures followed in the present study were performed in accordance with the ethical standards of the current revision of the Declaration of Helsinki. All patients provided written informed consent. Regarding twin A, samples were obtained from the center in which he is followed up (Christian Medical College, Vellore, India) for diagnostic purposes. Other patients were enrolled to The INForMeD Study (REC reference 16/LO/1376). All research analyses were conducted according to The INForMeD Study (REC reference 16/LO/1376) and ANNB_NBS_027 study (Public Health England Antenatal and Newborn screening research advisory committee, 13 March 2020).

### Reporting summary

Further information on research design is available in the Nature Research Reporting Summary linked to this article.

## Online content

Any methods, additional references, Nature Research reporting summaries, source data, extended data, supplementary information, acknowledgements, peer review information; details of author contributions and competing interests; and statements of data and code availability are available at 10.1038/s41591-022-01793-4.

## Supplementary information

Supplementary Information Supplementary Figure 1 and Tables 1–12.

Reporting Summary

Supplementary Table 1 Excel workbook with multiple tabs containing Supplementary Tables 1–12 (one table per tab). The tables have also been provided in the Supplementary Information PDF file.

## Data Availability

Sequence data have been deposited at the European Genome-phenome Archive, which is hosted by the European Bioinformatics Institute and the Centre for Genomic Regulation, under accession number EGAS00001005744. Data on all somatic SNVs, indels and structural rearrangements for twin A and twin B are available in Supplementary Tables 1 and 2. Source data for the Bayesian timing model are described in Supplementary Table 5. COSMIC Mutational Signatures v3.1 and Gene Curation data can be accessed at https://cancer.sanger.ac.uk/cosmic/. Source data described in Gerstung et al.^3^ and used in this study can be accessed via the ICGC Data Portal at https://dcc.icgc.org/pcawg. Clinical details of the monozygotic twins are reported in Results (‘Clinical findings’ section). Clinical details of the *JAK2V617F*-mutant MPN cohort are described in Results (‘Neonatal blood spot analysis in *JAK2V617F*-mutant MPN’ section) plus Supplementary Table 7.

## Code availability

Any bespoke code used in this paper can be found online at https://github.com/Wedge-lab/InUtero_MPN.
