# Supplementary material for: In utero origin of myelofibrosis presenting in adult monozygotic twins
Source: Nat Med. 2022 May 30;28(6):1207–11. doi: 10.1038/s41591-022-01793-4 (PMC9205768; doi:10.1038/s41591-022-01793-4)
Supplement: Supplementary file 1 — Supplementary Figure 1 and Tables 1–12. [file 41591_2022_1793_MOESM1_ESM.pdf]

nature portfolio

https://doi.org/10.1038/s41591-022-01793-4

Supplementary information

In utero origin of myelofibrosis presenting in adult monozygotic twins

In the format provided by the authors and unedited

Supplementary Information

Supplement to: Sousos N. et al. *In utero* origin of myelofibrosis presenting in adult monozygotic twins.

Table of Contents

Supplementary Figure 1. Gating strategy for (a) T cell flow cytometry analysis and (b) HSPC index sorting................ i

Supplementary Table 1. Catalogue of somatic substitutions and indels in twin A and twin B................ 1

Supplementary Table 2. Catalogue of somatic structural variants in twin A and twin B................ 38

Supplementary Table 3. Summary of somatic mutations shared between twin A and twin B................ 39

Supplementary Table 4. Summary of non-silent coding variants in twin A and twin B................ 41

Supplementary Table 5. Data for time to the most recent common ancestor (MRCA) estimation................ 42

Supplementary Table 6. Summary of the shared somatic variants and unique somatic variants for each twin, used in whole genome sequencing validation at the single-cell level................ 43

Supplementary Table 7. Overview of patient clinical information for the JAK2V617F-mutant myeloproliferative neoplasm cohort................ 44

Supplementary Table 8. Flow cytometry and cell sorting panels used in granulocyte, T cell and HSPC analysis and sorting................ 45

Supplementary Table 9. Catalogue of oligonucleotides used in the polymerase chain reaction assays................ 46

Supplementary Table 10. Catalogue of the genes targeted in the next-generation sequencing panel................ 47

Supplementary Table 11. Summary of multidimensional mutation clustering.. 49

Supplementary Table 12. Additional candidate cancer genes................ 71

APPENDIX A. Reporting Summary................ 72

Supplementary Figure 1. Gating strategy for (a) T cell flow cytometry analysis and (b) HSPC index sorting

CMP, common myeloid progenitor; Lin, Lineage; MEP, megakaryocyte-erythroid progenitor; GMP, granulocyte macrophage progenitor; HSC, hematopoietic stem cell; MPP, multi-potent progenitor; LMPP, lymphoid-primed multi-potent progenitor.

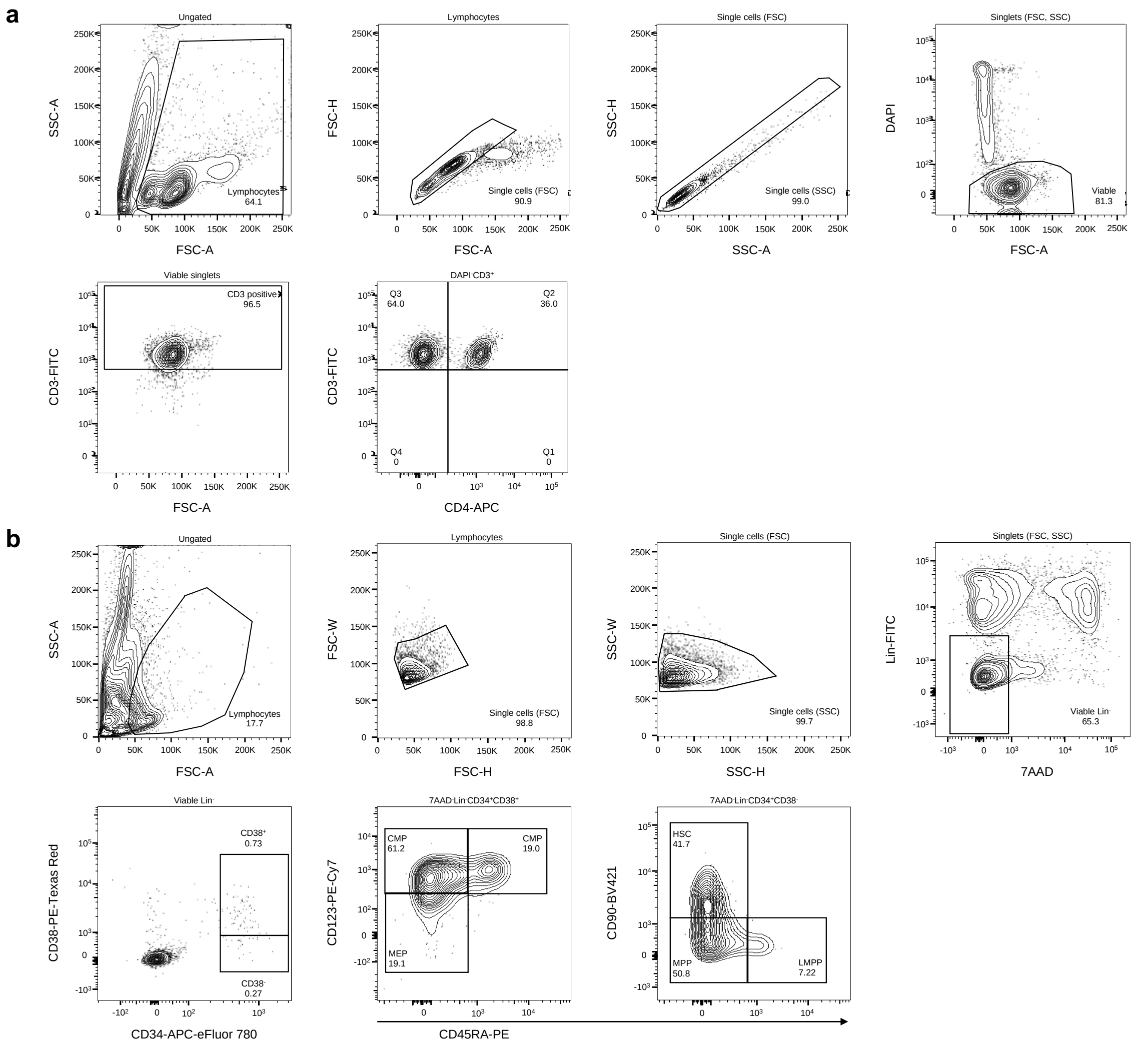

Supplementary Table 1. Catalogue of somatic substitutions and indels in twin A and twin B

Individual	Chr	Pos	Ref	Alt	Mutation_type	Annotation	Gene	Existing_variation
Twin_A	1	7048875	C	T	Substitution	intron_variant	*CAMTA1*	rs190299624
Twin_A	1	7276191	T	TA	Insertion	intron_variant	*CAMTA1*	rs34532883
Twin_A	1	7932734	TAC	T	Deletion	intron_variant	*TNFRSF9*	rs3841802
Twin_A	1	17778871	G	A	Substitution	intron_variant	*ACTL8*	-
Twin_A	1	19011134	G	A	Substitution	regulatory_region_variant	-	-
Twin_A	1	24201443	G	A	Substitution	intron_variant	*LINC02800*	-
Twin_A	1	25634524	A	G	Substitution	intron_variant	*MAN1C1*	-
Twin_A	1	26355029	G	A	Substitution	upstream_gene_variant	*CRYBG2*	-
Twin_A	1	32905284	G	GT	Insertion	upstream_gene_variant	*TMEM54*	rs201477428
Twin_A	1	36908778	GGT	G	Deletion	intron_variant	*GRIK3*	rs56228690
Twin_A	1	53327841	CA	C	Deletion	frameshift_variant	*LRP8*	rs761955852
Twin_A	1	54128162	C	CT	Insertion	downstream_gene_variant	*CDCP2*	rs35156752
Twin_A	1	58795504	G	GA	Insertion	intron_variant	*LINC01135*	rs773735239
Twin_A	1	58846527	T	C	Substitution	intron_variant	*LINC01135*	rs1310251048
Twin_A	1	62922910	C	A	Substitution	intergenic_variant	-	-
Twin_A	1	81244570	C	T	Substitution	intergenic_variant	-	-
Twin_A	1	82141981	T	TA	Insertion	intergenic_variant	-	rs1036875026
Twin_A	1	91064439	G	T	Substitution	intergenic_variant	-	rs556744289
Twin_A	1	91598893	G	A	Substitution	upstream_gene_variant	*AL714022.1*	-
Twin_A	1	93828702	C	CT	Insertion	intron_variant	*BCAR3*	rs969811086
Twin_A	1	106288464	G	T	Substitution	intergenic_variant	-	rs183684643
Twin_A	1	111854251	A	G	Substitution	intron_variant	*KCND3*	-
Twin_A	1	112723366	C	A	Substitution	intron_variant	*TAFA3*	-
Twin_A	1	117227167	C	T	Substitution	intergenic_variant	-	rs1249405708
Twin_A	1	122502993	T	A	Substitution	intergenic_variant	-	rs1271741426
Twin_A	1	123009960	C	A	Substitution	intergenic_variant	-	rs1355262045
Twin_A	1	144518342	G	A	Substitution	intergenic_variant	-	rs1289463903
Twin_A	1	152011365	A	G	Substitution	intron_variant	*AL450992.1*	-
Twin_A	1	152568644	A	G	Substitution	upstream_gene_variant	*LCE3E*	-
Twin_A	1	155136742	C	CA	Insertion	intron_variant	*SLC50A1*	rs879631277
Twin_A	1	155442289	T	TA	Insertion	intron_variant	*ASH1L*	rs1016155747
Twin_A	1	155643901	C	T	Substitution	intron_variant	*AL353807.3*	-
Twin_A	1	164325871	G	A	Substitution	intergenic_variant	-	-
Twin_A	1	167888410	A	AT	Insertion	intron_variant	*ADCY10*	rs930197268
Twin_A	1	172874706	T	C	Substitution	intron_variant	*AL031599.1*	-
Twin_A	1	173897024	T	A	Substitution	3_prime_UTR_variant	*ZBTB37*	rs1000471968
Twin_A	1	178136491	C	T	Substitution	intron_variant	*RASAL2*	-

Twin_A	1	182688357	GCT	G	Deletion	upstream_gene_variant	*RGS8*	rs57313959
Twin_A	1	182732230	TTC	T	Deletion	upstream_gene_variant	*AL353778.1*	rs1191757083
Twin_A	1	185587702	C	A	Substitution	intron_variant	*LINC01350*	-
Twin_A	1	185717034	C	T	Substitution	regulatory_region_variant	-	rs74766303
Twin_A	1	188790583	C	T	Substitution	intergenic_variant	-	-
Twin_A	1	190678569	TGTTG	T	Deletion	intron_variant	*LINC01720*	-
Twin_A	1	191116119	T	TTA	Insertion	intron_variant	*AL138927.1*	rs386354456
Twin_A	1	191963370	T	TC	Insertion	intron_variant	*LINC02770*	-
Twin_A	1	196771008	G	T	Substitution	upstream_gene_variant	*CFHR3*	-
Twin_A	1	199078846	G	GA	Insertion	downstream_gene_variant	*LINC01221*	rs914403440
Twin_A	1	213790163	G	T	Substitution	intron_variant	*AC096639.1*	-
Twin_A	1	218758531	G	A	Substitution	intergenic_variant	-	-
Twin_A	1	237523679	G	A	Substitution	intron_variant	*RYR2*	rs1165384474
Twin_A	1	238480115	T	C	Substitution	downstream_gene_variant	*LINC01139*	rs1384492382
Twin_A	1	240415525	T	TATA	Insertion	intron_variant	*FMN2*	rs202218606
Twin_A	1	243567256	GC	G	Deletion	intron_variant	*AKT3*	-
Twin_A	2	184966	A	T	Substitution	intergenic_variant	-	-
Twin_A	2	2982012	G	A	Substitution	intron_variant	*LINC01250*	rs1038718094
Twin_A	2	5026216	TA	T	Deletion	intergenic_variant	-	rs1002511716
Twin_A	2	11568288	G	A	Substitution	intron_variant	*GREB1*	rs1225502656
Twin_A	2	12436887	T	G	Substitution	intron_variant	*MIR3681HG*	-
Twin_A	2	13111603	C	A	Substitution	intron_variant	*AC093912.1*	-
Twin_A	2	17632861	A	C	Substitution	intron_variant	*VSNL1*	-
Twin_A	2	19486986	C	CT	Insertion	intron_variant	*LINC01808*	rs796988189
Twin_A	2	24266260	C	T	Substitution	intron_variant	*ITSN2*	-
Twin_A	2	25819904	C	CTTAT	Insertion	intron_variant	*ASXL2*	rs879625913
Twin_A	2	29907671	C	G	Substitution	downstream_gene_variant	*AC106870.1*	rs1275974026
Twin_A	2	31671794	C	T	Substitution	intergenic_variant	-	rs936962348
Twin_A	2	32351096	CT	C	Deletion	intergenic_variant	-	rs747843331
Twin_A	2	35209815	A	G	Substitution	intergenic_variant	-	-
Twin_A	2	38570879	C	CA	Insertion	intron_variant	*HNRNPLL*	rs796515789
Twin_A	2	49012790	C	T	Substitution	intron_variant	*FSHR*	rs1051686975
Twin_A	2	54291307	G	A	Substitution	intron_variant	*ACYP2*	-
Twin_A	2	62581105	A	G	Substitution	intron_variant	*AC092155.1*	rs1357993052
Twin_A	2	64592352	T	TA	Insertion	3_prime_UTR_variant	*AFTPH*	rs879897290
Twin_A	2	66088937	C	T	Substitution	downstream_gene_variant	*AC007389.1*	-
Twin_A	2	78204244	C	A	Substitution	intron_variant	*AC012494.1*	-
Twin_A	2	78265814	T	C	Substitution	intron_variant	*AC012494.1*	-
Twin_A	2	79075253	T	TA	Insertion	intergenic_variant	-	rs200270320
Twin_A	2	83704515	C	T	Substitution	intergenic_variant	-	-
Twin_A	2	86143605	A	AT	Insertion	downstream_gene_variant	*PTCD3*	rs952364218
Twin_A	2	86786422	C	T	Substitution	intron_variant	*CD8A*	-

Twin_A	2	87046122	A	C	Substitution	intron_variant	*ANAPC1P2*	rs79742084
Twin_A	2	89100821	C	G	Substitution	upstream_gene_variant	*IGKV1*	-
Twin_A	2	90026936	G	A	Substitution	downstream_gene_variant	*IGKV6D*	rs568946974
Twin_A	2	93865725	G	C	Substitution	intergenic_variant	-	-
Twin_A	2	98022727	T	TTG	Insertion	regulatory_region_variant	-	rs371216469
Twin_A	2	105464070	C	CA	Insertion	intergenic_variant	-	rs1161295349
Twin_A	2	107747999	TAC	T	Deletion	intron_variant	*LINC01885*	rs146173650
Twin_A	2	113030007	C	T	Substitution	intron_variant	*IL36B*	rs771673800
Twin_A	2	115188061	AAG	A	Deletion	intron_variant	*DPP10*	rs59672278
Twin_A	2	118358987	C	T	Substitution	intergenic_variant	-	-
Twin_A	2	119522301	G	GA	Insertion	intron_variant	*SCTR*	rs797004076
Twin_A	2	122548088	A	G	Substitution	intron_variant	*AC011246.1*	rs893268258
Twin_A	2	124139117	G	GT	Insertion	downstream_gene_variant	*CNTNAP5*	rs1052761072
Twin_A	2	126462653	G	A	Substitution	intergenic_variant	-	rs147150064
Twin_A	2	126802154	C	T	Substitution	regulatory_region_variant	-	rs1390212792
Twin_A	2	130631605	G	T	Substitution	intron_variant	*POTEJ*	-
Twin_A	2	135703624	G	GT	Insertion	intron_variant	*R3HDM1*	rs1194873094
Twin_A	2	139236448	C	A	Substitution	intergenic_variant	-	-
Twin_A	2	140606025	AAG	A	Deletion	intron_variant	*LRP1B*	rs541895855
Twin_A	2	142582383	T	C	Substitution	regulatory_region_variant	-	-
Twin_A	2	145569408	G	A	Substitution	non_coding_transcript_ exon_variant	*AC092484.1*	-
Twin_A	2	145603291	G	A	Substitution	non_coding_transcript_ exon_variant	*AC079163.1*	rs373802799
Twin_A	2	148242718	A	T	Substitution	intron_variant	*MBD5*	-
Twin_A	2	153181357	C	CA	Insertion	intron_variant	*AC011901.1*	rs34588886
Twin_A	2	153730111	G	GA	Insertion	intergenic_variant	-	rs929649211
Twin_A	2	154639539	G	A	Substitution	intergenic_variant	-	-
Twin_A	2	162949464	C	G	Substitution	intergenic_variant	-	-
Twin_A	2	163100502	C	A	Substitution	intergenic_variant	-	-
Twin_A	2	167809460	T	G	Substitution	intergenic_variant	-	-
Twin_A	2	174496556	C	A	Substitution	intergenic_variant	-	-
Twin_A	2	175727129	G	T	Substitution	intergenic_variant	-	rs182157918
Twin_A	2	181542157	CAGTG	C	Deletion	intron_variant	*CERKL*	-
Twin_A	2	183736872	T	C	Substitution	intergenic_variant	-	-
Twin_A	2	193562677	CGT	C	Deletion	intergenic_variant	-	rs59053823
Twin_A	2	194108461	C	CT	Insertion	intergenic_variant	-	rs879718049
Twin_A	2	196497946	T	C	Substitution	intron_variant	*HECW2*	-
Twin_A	2	199505098	A	G	Substitution	intergenic_variant	-	-
Twin_A	2	199984230	A	G	Substitution	intron_variant	*MAIP1*	-
Twin_A	2	200975338	A	T	Substitution	downstream_gene_variant	*FAM126B*	-
Twin_A	2	202920157	C	CT	Insertion	intron_variant	*CARF*	rs969806621
Twin_A	2	217649629	G	C	Substitution	intron_variant	*DIRC3*	-
Twin_A	2	218749467	C	CT	Insertion	intron_variant	*TTLL4*	rs368058156

Twin_A	2	221924308	A	G	Substitution	intergenic_variant	-	-
Twin_A	2	227692889	T	C	Substitution	intron_variant	*SLC19A3*	-
Twin_A	2	228021121	C	T	Substitution	intron_variant	*SPHKAP*	rs531054237
Twin_A	2	234919271	G	GT	Insertion	intergenic_variant	-	rs893811865
Twin_A	2	235048421	C	A	Substitution	intron_variant	*SH3BP4*	-
Twin_A	2	235142193	T	C	Substitution	downstream_gene_variant	*CEP19P1*	rs953123210
Twin_A	2	236024598	A	G	Substitution	intron_variant	*AGAP1*	-
Twin_A	2	236165626	A	C	Substitution	3_prime_UTR_variant	*GBX2*	-
Twin_A	2	240285993	G	A	Substitution	upstream_gene_variant	*U3*	rs79159698
Twin_A	2	240487775	C	T	Substitution	intron_variant	*ANKMY1*	-
Twin_A	3	1259968	A	G	Substitution	intron_variant	*CNTN6*	-
Twin_A	3	6904723	G	GT	Insertion	intron_variant	*GRM7*	rs143641576
Twin_A	3	9646697	T	A	Substitution	upstream_gene_variant	*MTMR14*	rs1001727401
Twin_A	3	26440532	A	T	Substitution	intergenic_variant	-	-
Twin_A	3	29763656	A	C	Substitution	intron_variant	*RBMS3*	-
Twin_A	3	32019778	T	C	Substitution	intron_variant	*OSBPL10*	rs545152631
Twin_A	3	32526653	CCA	C	Deletion	3_prime_UTR_variant	*DYNC1LI1*	rs3836420
Twin_A	3	40432373	TGA	T	Deletion	downstream_gene_variant	*ENTPD3*	rs142976010
Twin_A	3	42455599	G	T	Substitution	intergenic_variant	-	-
Twin_A	3	59893891	T	C	Substitution	intron_variant	*FHIT*	-
Twin_A	3	65074311	C	T	Substitution	intergenic_variant	-	-
Twin_A	3	71644657	A	T	Substitution	intron_variant	*AC097634.4*	rs11925350
Twin_A	3	72405852	G	A	Substitution	intron_variant	*RYBP*	-
Twin_A	3	74296285	G	A	Substitution	intron_variant	*CNTN3*	rs74970959
Twin_A	3	74299076	G	A	Substitution	intron_variant	*CNTN3*	rs149006907
Twin_A	3	74980380	T	A	Substitution	regulatory_region_variant	-	-
Twin_A	3	75692610	CTTA	C	Deletion	intron_variant	*ZNF717*	rs140744949
Twin_A	3	76086971	T	C	Substitution	intron_variant	*ROBO2*	-
Twin_A	3	80858231	C	T	Substitution	intergenic_variant	-	rs982669611
Twin_A	3	86921756	A	G	Substitution	intron_variant	*VGLL3*	-
Twin_A	3	90430217	C	G	Substitution	intergenic_variant	-	-
Twin_A	3	90965981	A	G	Substitution	intergenic_variant	-	rs1304555355
Twin_A	3	95430870	C	T	Substitution	intergenic_variant	-	rs562186321
Twin_A	3	103475498	G	A	Substitution	intergenic_variant	-	-
Twin_A	3	107699193	C	T	Substitution	intron_variant	*BBX*	-
Twin_A	3	117666302	C	CT	Insertion	intergenic_variant	-	rs560342962
Twin_A	3	118298534	T	C	Substitution	intron_variant	*AC068633.1*	-
Twin_A	3	124027883	G	A	Substitution	intergenic_variant	-	-
Twin_A	3	125726439	G	A	Substitution	downstream_gene_variant	*OR7E93P*	rs147522636
Twin_A	3	126102405	G	A	Substitution	downstream_gene_variant	*ALDH1L1*	rs1348872806
Twin_A	3	147257030	A	G	Substitution	intron_variant	*AC092957.1*	-
Twin_A	3	148087688	G	GT	Insertion	intron_variant	*LINC02032*	rs35840438

Twin_A	3	152974761	T	C	Substitution	intergenic_variant	-	rs137865769
Twin_A	3	153068733	G	A	Substitution	downstream_gene_variant	*HMGN2P13*	-
Twin_A	3	153706759	T	G	Substitution	intron_variant	*LINC02006*	-
Twin_A	3	156297775	C	T	Substitution	intron_variant	*KCNAB1*	rs370869760
Twin_A	3	160115640	G	A	Substitution	intron_variant	*IL12A-AS1*	rs985125306
Twin_A	3	165572607	C	T	Substitution	intron_variant	*LINC01322*	-
Twin_A	3	168769424	G	A	Substitution	intron_variant	*EGFEM1P*	rs536413401
Twin_A	3	168769557	T	C	Substitution	intron_variant	*EGFEM1P*	rs575522209
Twin_A	3	183534087	TTACTTTT AAAGTAC TTTAAAA GTACTTTA CTTTTAAA GTACTTTG	T	Deletion	intron_variant	*KLHL6*	rs1193720974
Twin_A	3	187366417	G	A	Substitution	upstream_gene_variant	*RTP4*	-
Twin_A	4	3011910	G	A	Substitution	intron_variant	*GRK4*	rs1056281673
Twin_A	4	5718308	T	A	Substitution	intron_variant	*EVC*	-
Twin_A	4	15955763	G	C	Substitution	downstream_gene_variant	*FGFBP2*	-
Twin_A	4	17001308	C	T	Substitution	intron_variant	*AC106894.1*	rs1437750527
Twin_A	4	17415320	G	A	Substitution	intergenic_variant	-	-
Twin_A	4	22103283	A	G	Substitution	intron_variant	*AC096719.1*	-
Twin_A	4	26343555	G	GT	Insertion	intron_variant	*RBPJ*	rs150586776
Twin_A	4	26935882	A	G	Substitution	intron_variant	*STIM2*	rs1392350220
Twin_A	4	31169536	C	A	Substitution	upstream_gene_variant	*LINC02497*	-
Twin_A	4	35852584	CAG	C	Deletion	intergenic_variant	-	rs150417183
Twin_A	4	38452605	G	A	Substitution	non_coding_transcript_ exon_variant	*LINC01258*	rs527412993
Twin_A	4	44413264	T	G	Substitution	intron_variant	*KCTD8*	-
Twin_A	4	52545084	CCTCT	C	Deletion	intron_variant	*AC097522.2*	rs748919064
Twin_A	4	54682436	C	CT	Insertion	intron_variant	*KIT*	rs368264394
Twin_A	4	55961258	A	G	Substitution	intron_variant	*CEP135*	-
Twin_A	4	61895466	AAAT	A	Deletion	upstream_gene_variant	*ADGRL3*	rs10603633
Twin_A	4	63566747	C	T	Substitution	intergenic_variant	-	-
Twin_A	4	66771456	G	C	Substitution	intergenic_variant	-	-
Twin_A	4	69630424	C	T	Substitution	intron_variant	*UGT2A1*	-
Twin_A	4	74248292	T	C	Substitution	intron_variant	*MTHFD2L*	-
Twin_A	4	74655308	C	T	Substitution	regulatory_region_variant	-	rs781899546
Twin_A	4	74668604	C	CT	Insertion	regulatory_region_variant	-	rs1418001328
Twin_A	4	76432023	G	T	Substitution	upstream_gene_variant	*SHROOM3*	-
Twin_A	4	82531664	A	G	Substitution	intron_variant	*TMEM150C*	-
Twin_A	4	105234449	TA	T	Deletion	frameshift_variant	*TET2*	-
Twin_A	4	106394908	AAT	A	Deletion	intergenic_variant	-	rs140334876
Twin_A	4	109876682	A	G	Substitution	downstream_gene_variant	*LRIT3*	-
Twin_A	4	110522189	C	CT	Insertion	intron_variant	*ENPEP*	rs539673220
Twin_A	4	111359412	C	T	Substitution	intergenic_variant	-	-
Twin_A	4	113822564	A	AT	Insertion	regulatory_region_variant	-	rs34730469

Twin_A	4	115289705	A	T	Substitution	intergenic_variant	-	rs201585293
Twin_A	4	119833655	A	T	Substitution	intergenic_variant	-	-
Twin_A	4	122600220	C	T	Substitution	intergenic_variant	-	-
Twin_A	4	124416683	A	AT	Insertion	intergenic_variant	-	rs112541914
Twin_A	4	134850961	G	A	Substitution	intergenic_variant	-	-
Twin_A	4	145648023	AT	A	Deletion	upstream_gene_variant	*MMAA*	rs771723312
Twin_A	4	160621868	A	G	Substitution	intergenic_variant	-	-
Twin_A	4	161252551	T	C	Substitution	regulatory_region_variant	-	-
Twin_A	4	161288198	G	GA	Insertion	intergenic_variant	-	rs969053461
Twin_A	4	162816440	T	C	Substitution	intron_variant	*AC021134.1*	-
Twin_A	4	180265089	G	A	Substitution	intergenic_variant	-	-
Twin_A	4	180667100	G	A	Substitution	regulatory_region_variant	-	rs563546983
Twin_A	4	182387149	G	A	Substitution	intron_variant	*TENM3*	rs983423020
Twin_A	4	183453115	T	C	Substitution	downstream_gene_variant	*CDKN2AIP*	-
Twin_A	4	185461063	T	C	Substitution	missense_variant	*CCDC110*	rs773624975
Twin_A	4	186034311	C	T	Substitution	intergenic_variant	-	rs199807699
Twin_A	4	187661068	G	T	Substitution	downstream_gene_variant	*AC097521.1*	-
Twin_A	4	188628950	T	TA	Insertion	intron_variant	*LINC01060*	-
Twin_A	4	105259633*	G	A	Substitution	missense_variant	*TET2*	rs1456238164
Twin_A	5	2114089	AT	A	Deletion	intron_variant	*AC124852.1*	rs374364105
Twin_A	5	4579966	C	T	Substitution	intron_variant	*AC106799.2*	-
Twin_A	5	6643035	G	GT	Insertion	intron_variant	*SRD5A1*	rs35377021
Twin_A	5	7817145	G	GA	Insertion	intron_variant	*ADCY2*	rs57771304
Twin_A	5	7987372	C	T	Substitution	intergenic_variant	-	rs1320304875
Twin_A	5	10921181	G	GT	Insertion	intergenic_variant	-	rs60156777
Twin_A	5	10937820	A	G	Substitution	intergenic_variant	-	rs561589454
Twin_A	5	12387040	G	A	Substitution	intergenic_variant	-	-
Twin_A	5	18474168	C	T	Substitution	intergenic_variant	-	-
Twin_A	5	24399357	G	A	Substitution	intergenic_variant	-	rs868374455
Twin_A	5	33779372	G	A	Substitution	intron_variant	*ADAMTS12*	-
Twin_A	5	35831884	C	T	Substitution	upstream_gene_variant	*AC137810.1*	-
Twin_A	5	36636178	G	T	Substitution	intron_variant	*SLC1A3*	-
Twin_A	5	41753270	TAC	T	Deletion	intron_variant	*OXCT1*	rs36095905
Twin_A	5	52121218	T	G	Substitution	intergenic_variant	-	rs111904173
Twin_A	5	56387833	C	T	Substitution	intergenic_variant	-	-
Twin_A	5	58833324	TCA	T	Deletion	intron_variant	*RAB3C*	rs571940681
Twin_A	5	58833331	C	T	Substitution	intron_variant	*RAB3C*	rs199895867
Twin_A	5	63548792	G	C	Substitution	intergenic_variant	-	-
Twin_A	5	69055388	A	T	Substitution	intergenic_variant	-	-
Twin_A	5	73740730	C	T	Substitution	intron_variant	*ARHGEF28*	rs934596518
Twin_A	5	75285945	G	T	Substitution	intergenic_variant	-	-
Twin_A	5	78363812	A	AT	Insertion	upstream_gene_variant	*SCAMP1*	rs566623870

Twin_A	5	79269736	A	AT	Insertion	intron_variant	*JMY*	rs905517032
Twin_A	5	80583714	T	TGC	Insertion	intergenic_variant	-	rs113455475
Twin_A	5	81377269	G	A	Substitution	intron_variant	*ACOT12*	-
Twin_A	5	82451636	A	G	Substitution	intergenic_variant	-	-
Twin_A	5	85688616	C	T	Substitution	intergenic_variant	-	-
Twin_A	5	86606508	G	GT	Insertion	intergenic_variant	-	rs1353717748
Twin_A	5	91742536	G	C	Substitution	intron_variant	*AC093298.2*	-
Twin_A	5	93374643	C	T	Substitution	intron_variant	*NR2F1*	rs529601636
Twin_A	5	95231337	GAT	G	Deletion	intron_variant	*MCTP1*	rs138755290
Twin_A	5	95701747	A	G	Substitution	upstream_gene_variant	*SPATA9*	-
Twin_A	5	97765568	G	A	Substitution	intergenic_variant	-	-
Twin_A	5	100034257	G	A	Substitution	intergenic_variant	-	-
Twin_A	5	100071988	T	A	Substitution	upstream_gene_variant	*AC092278.1*	rs78484001
Twin_A	5	100155088	G	GT	Insertion	downstream_gene_variant	*RNU6*	rs5869895
Twin_A	5	100295778	C	T	Substitution	intergenic_variant	-	-
Twin_A	5	105654988	C	T	Substitution	intergenic_variant	-	rs1173103088
Twin_A	5	105941453	T	G	Substitution	intergenic_variant	-	-
Twin_A	5	111449354	A	G	Substitution	intron_variant	*CAMK4*	-
Twin_A	5	115666098	C	T	Substitution	regulatory_region_variant	-	-
Twin_A	5	116208129	T	C	Substitution	intron_variant	*COMMD10*	-
Twin_A	5	116797618	T	TA	Insertion	intergenic_variant	-	rs370022009
Twin_A	5	123223704	C	A	Substitution	intergenic_variant	-	-
Twin_A	5	130388901	G	A	Substitution	upstream_gene_variant	*RNU7*	rs1308696583
Twin_A	5	131362688	C	T	Substitution	intron_variant	*CDC42SE2*	-
Twin_A	5	131622186	T	TA	Insertion	intron_variant	*RAPGEF6*	rs368148920
Twin_A	5	142866780	T	G	Substitution	intron_variant	*ARHGAP26*	-
Twin_A	5	147611565	T	C	Substitution	intron_variant	*JAKMIP2*	-
Twin_A	5	152460283	G	A	Substitution	intron_variant	*AC008571.2*	-
Twin_A	5	155157242	C	T	Substitution	intron_variant	*AC010476.2*	rs950323196
Twin_A	5	162912266	C	A	Substitution	intron_variant	*AC113414.1*	-
Twin_A	5	163087087	G	GA	Insertion	intron_variant	*AC113414.1*	rs979728237
Twin_A	5	166031221	GTAGA	G	Deletion	upstream_gene_variant	*RPL7P20*	-
Twin_A	6	7238595	C	CT	Insertion	intron_variant	*RREB1*	rs796560101
Twin_A	6	8925748	C	T	Substitution	intron_variant	*HULC*	-
Twin_A	6	9330828	G	A	Substitution	intergenic_variant	-	-
Twin_A	6	15203913	A	AT	Insertion	intergenic_variant	-	rs1241556169
Twin_A	6	16644979	C	T	Substitution	intron_variant	*ATXN1*	-
Twin_A	6	19952193	G	A	Substitution	intergenic_variant	-	rs779233914
Twin_A	6	31570888	C	T	Substitution	upstream_gene_variant	*LTA*	rs956044849
Twin_A	6	36883002	G	T	Substitution	upstream_gene_variant	*C6orf89*	-
Twin_A	6	40639879	ACT	A	Deletion	intergenic_variant	-	rs35021402
Twin_A	6	40934220	G	A	Substitution	intergenic_variant	-	-

Twin_A	6	46776061	TA	T	Deletion	intergenic_variant	-	-
Twin_A	6	46995372	G	GT	Insertion	downstream_gene_variant	*ADGRF1*	rs142966227
Twin_A	6	58029106	G	A	Substitution	intron_variant	*AL445250.1*	rs1539609
Twin_A	6	60497665	T	C	Substitution	intron_variant	*AC244258.1*	-
Twin_A	6	62533854	G	A	Substitution	intergenic_variant	-	rs1050425018
Twin_A	6	70161977	C	A	Substitution	intron_variant	*COL19A1*	rs754784425
Twin_A	6	71671466	A	T	Substitution	intergenic_variant	-	-
Twin_A	6	80070589	C	T	Substitution	upstream_gene_variant	*AL591135.1*	rs113123867
Twin_A	6	90318383	C	CT	Insertion	upstream_gene_variant	*AL132996.1*	rs1230991775
Twin_A	6	93048770	T	TA	Insertion	regulatory_region_variant	-	rs1214397059
Twin_A	6	93696846	GTC	G	Deletion	intergenic_variant	-	rs142217537
Twin_A	6	95778674	T	A	Substitution	intergenic_variant	-	-
Twin_A	6	97233067	G	GA	Insertion	intron_variant	*MMS22L*	rs573093148
Twin_A	6	103570525	T	TTA	Insertion	intergenic_variant	-	rs533182054
Twin_A	6	111252036	C	T	Substitution	intron_variant	*AL360227.1*	-
Twin_A	6	117652832	G	A	Substitution	intergenic_variant	-	-
Twin_A	6	119799146	C	CT	Insertion	intergenic_variant	-	rs1218766914
Twin_A	6	119917789	C	G	Substitution	intergenic_variant	-	-
Twin_A	6	123368975	C	G	Substitution	intron_variant	*TRDN*	-
Twin_A	6	124380080	A	T	Substitution	intron_variant	*NKAIN2*	-
Twin_A	6	126389883	T	C	Substitution	intron_variant	*AL356534.1*	-
Twin_A	6	131059163	G	A	Substitution	intron_variant	*EPB41L2*	-
Twin_A	6	131399570	C	G	Substitution	intergenic_variant	-	-
Twin_A	6	134069108	C	T	Substitution	intergenic_variant	-	rs187112667
Twin_A	6	140986451	A	C	Substitution	intergenic_variant	-	-
Twin_A	6	145547033	T	G	Substitution	intron_variant	*EPM2A*	-
Twin_A	6	159660393	C	T	Substitution	intron_variant	*AL078604.4*	rs769079326
Twin_A	6	159881330	C	CAT	Insertion	intergenic_variant	-	rs10672852
Twin_A	6	166093829	G	T	Substitution	upstream_gene_variant	*AL627443.3*	-
Twin_A	6	166187121	G	GA	Insertion	intergenic_variant	-	-
Twin_A	7	4979367	T	C	Substitution	intron_variant	*RNF216P1*	rs868499198
Twin_A	7	5000741	A	G	Substitution	downstream_gene_variant	*RNF216P1*	rs1338617888
Twin_A	7	7056369	G	A	Substitution	intron_variant	*AC079804.3*	-
Twin_A	7	9227657	G	A	Substitution	regulatory_region_variant	-	rs999966239
Twin_A	7	21817695	A	AT	Insertion	intron_variant	*DNAH11*	rs201247500
Twin_A	7	30831221	C	T	Substitution	intron_variant	*MINDY4*	-
Twin_A	7	36071829	A	G	Substitution	intergenic_variant	-	rs757946495
Twin_A	7	38945518	G	GA	Insertion	intergenic_variant	-	rs796386392
Twin_A	7	41732610	A	G	Substitution	intron_variant	*INHBA*	-
Twin_A	7	43378810	C	CA	Insertion	intron_variant	*HECW1*	rs202007736
Twin_A	7	44090851	C	T	Substitution	intergenic_variant	-	-
Twin_A	7	44510030	T	A	Substitution	downstream_gene_variant	*NPC1L1*	-

Twin_A	7	49269510	AT	A	Deletion	intergenic_variant	-	rs1265301921
Twin_A	7	49269512	A	C	Substitution	intergenic_variant	-	rs984581632
Twin_A	7	53068163	G	A	Substitution	intergenic_variant	-	rs935736922
Twin_A	7	53091502	C	T	Substitution	intergenic_variant	-	-
Twin_A	7	53364914	G	A	Substitution	upstream_gene_variant	*RNU1*	rs34714639
Twin_A	7	54587046	C	A	Substitution	intergenic_variant	-	-
Twin_A	7	62529040	C	T	Substitution	intergenic_variant	-	rs555323358
Twin_A	7	64713934	GTATT	G	Deletion	downstream_gene_variant	*ZNF107*	rs369110012
Twin_A	7	67857057	G	A	Substitution	intergenic_variant	-	rs1319269938
Twin_A	7	68076463	CAT	C	Deletion	intergenic_variant	-	rs139100940
Twin_A	7	69630257	A	G	Substitution	intron_variant	*AUTS2*	-
Twin_A	7	73627805	C	T	Substitution	upstream_gene_variant	*MLXIPL*	-
Twin_A	7	81046412	CT	C	Deletion	intergenic_variant	-	rs1381425510
Twin_A	7	88505688	G	A	Substitution	intron_variant	*AC002069.2*	-
Twin_A	7	89031211	GTA	G	Deletion	intron_variant	*ZNF804B*	-
Twin_A	7	90565456	G	GT	Insertion	intron_variant	*CDK14*	rs373870738
Twin_A	7	95205017	CCA	C	Deletion	intron_variant	*PPP1R9A*	rs143843788
Twin_A	7	95298520	G	C	Substitution	3_prime_UTR_variant	*PON1*	-
Twin_A	7	96055652	T	TA	Insertion	intron_variant	*DYNC1I1*	rs11374590
Twin_A	7	98561959	C	A	Substitution	intergenic_variant	-	-
Twin_A	7	102065090	G	GT	Insertion	intron_variant	*CUX1*	rs562493293
Twin_A	7	109185877	G	A	Substitution	intergenic_variant	-	rs1228545900
Twin_A	7	110615318	C	T	Substitution	intergenic_variant	-	-
Twin_A	7	122016458	C	A	Substitution	intron_variant	*PTPRZ1*	-
Twin_A	7	124142825	C	T	Substitution	intron_variant	*AC006148.1*	-
Twin_A	7	128429252	C	CT	Insertion	downstream_gene_variant	*AC010655.1*	rs56056515
Twin_A	7	129776973	A	G	Substitution	upstream_gene_variant	*MIR96*	rs1401666012
Twin_A	7	137296579	A	G	Substitution	intron_variant	*PTN*	-
Twin_A	7	139519359	T	TA	Insertion	upstream_gene_variant	*CLEC2L*	rs1220935636
Twin_A	7	149998259	A	G	Substitution	upstream_gene_variant	*AC092681.2*	rs2969815
Twin_A	7	153528906	T	C	Substitution	intergenic_variant	-	-
Twin_A	7	154611485	A	G	Substitution	intron_variant	*DPP6*	-
Twin_A	8	503037	C	T	Substitution	intron_variant	*TDRP*	rs1166917437
Twin_A	8	13876611	G	GA	Insertion	regulatory_region_variant	-	rs34968549
Twin_A	8	17691704	G	GT	Insertion	intron_variant	*MTUS1*	rs200014137
Twin_A	8	19550828	C	T	Substitution	intron_variant	*CSGALNACT1*	-
Twin_A	8	25892225	T	C	Substitution	intron_variant	*EBF2*	-
Twin_A	8	30978684	T	C	Substitution	downstream_gene_variant	*AC008066.1*	-
Twin_A	8	36379501	CAT	C	Deletion	intron_variant	*AC090809.1*	rs148082032
Twin_A	8	36570758	T	A	Substitution	intron_variant	*AC090809.1*	-
Twin_A	8	39656195	G	GA	Insertion	intron_variant	*ADAM18*	rs201971418
Twin_A	8	48680993	A	G	Substitution	intron_variant	*AC022915.2*	-

Twin_A	8	51711248	T	C	Substitution	intron_variant	*PXDNL*	COSV62492491
Twin_A	8	53511516	G	GA	Insertion	downstream_gene_variant	*AC022034.1*	rs535409810
Twin_A	8	61457436	G	A	Substitution	intron_variant	*CLVS1*	-
Twin_A	8	63967261	A	AT	Insertion	intron_variant	*LINC01414*	rs5891933
Twin_A	8	64128898	G	A	Substitution	intron_variant	*LINC01414*	rs545104948
Twin_A	8	65916408	G	A	Substitution	intergenic_variant	-	-
Twin_A	8	68253460	T	TA	Insertion	regulatory_region_variant	-	rs1000407340
Twin_A	8	72453042	C	T	Substitution	intergenic_variant	-	rs1051744174
Twin_A	8	77781391	C	CT	Insertion	intergenic_variant	-	rs1198479285
Twin_A	8	83358311	GGCTACA GACAAAC CTTCAAT GGA	G	Deletion	intergenic_variant	-	-
Twin_A	8	86318421	G	GA	Insertion	intron_variant	*SLC7A13*	rs34723665
Twin_A	8	88356215	C	A	Substitution	intron_variant	*AC090578.1*	-
Twin_A	8	95037819	G	GA	Insertion	intron_variant	*NDUFAF6*	rs59511473
Twin_A	8	104362862	A	G	Substitution	intron_variant	*DPYS*	rs555618444
Twin_A	8	108043942	A	T	Substitution	intron_variant	*RSPO2*	-
Twin_A	8	115536798	G	GT	Insertion	intron_variant	*TRPS1*	rs900943605
Twin_A	8	117615713	ATCT	A	Deletion	regulatory_region_variant	-	-
Twin_A	8	124975564	C	G	Substitution	intron_variant	*ZNF572*	-
Twin_A	8	129273738	G	GT	Insertion	intron_variant	*CCDC26*	rs891751618
Twin_A	8	129372882	C	CA	Insertion	intron_variant	*CCDC26*	rs560030895
Twin_A	8	129733477	G	A	Substitution	downstream_gene_variant	*MTRF1LP2*	-
Twin_A	8	132428552	CAG	C	Deletion	intron_variant	*KCNQ3*	rs201318711
Twin_A	8	134856654	C	G	Substitution	intron_variant	*AC103764.1*	rs1321197388
Twin_A	8	135261370	G	C	Substitution	intron_variant	*LINC01591*	-
Twin_A	8	137128263	C	T	Substitution	intergenic_variant	-	rs1053069745
Twin_A	8	138139847	C	T	Substitution	intron_variant	*FAM135B*	rs947110030
Twin_A	8	143051626	G	A	Substitution	intron_variant	*C8orf31*	-
Twin_A	9	50071	AT	A	Deletion	intron_variant	*WASHC1*	-
Twin_A	9	395970	T	G	Substitution	intron_variant	*DOCK8*	-
Twin_A	9	1822815	C	T	Substitution	intergenic_variant	-	-
Twin_A	9	4316936	C	T	Substitution	intron_variant	*GLIS3*	-
Twin_A	9	5368234	T	C	Substitution	intron_variant	*PLGRKT*	-
Twin_A	9	14104419	G	GT	Insertion	intron_variant	*NFIB*	rs945961609
Twin_A	9	17180226	GT	G	Deletion	intron_variant	*CNTLN*	rs1264390576
Twin_A	9	19348792	G	GA	Insertion	intron_variant	*DENND4C*	rs367658826
Twin_A	9	23699353	A	G	Substitution	intron_variant	*ELAVL2*	rs568285627
Twin_A	9	24066549	G	A	Substitution	upstream_gene_variant	*AL513317.1*	rs895109680
Twin_A	9	28150104	C	A	Substitution	intron_variant	*LINGO2*	-
Twin_A	9	31556284	G	A	Substitution	intergenic_variant	-	rs12347583
Twin_A	9	39080916	C	T	Substitution	intron_variant	*CNTNAP3*	rs369395719
Twin_A	9	41655135	C	T	Substitution	upstream_gene_variant	*AL591926.2*	rs1226839471

Twin_A	9	45155466	G	C	Substitution	intergenic_variant	-	rs1476645657
Twin_A	9	61566797	T	C	Substitution	intergenic_variant	-	-
Twin_A	9	73087756	A	G	Substitution	intergenic_variant	-	-
Twin_A	9	77004114	C	A	Substitution	intergenic_variant	-	-
Twin_A	9	81050746	G	A	Substitution	intergenic_variant	-	rs764741538
Twin_A	9	82371224	C	T	Substitution	intron_variant	*AL162726.3*	-
Twin_A	9	83484424	C	CT	Insertion	intron_variant	*FRMD3*	-
Twin_A	9	84858874	G	GA	Insertion	intron_variant	*NTRK2*	rs965433677
Twin_A	9	87748283	T	TA	Insertion	regulatory_region_variant	-	rs143725130
Twin_A	9	88768716	G	A	Substitution	intron_variant	*AL390791.1*	rs548341913
Twin_A	9	96382496	C	CTA	Insertion	intron_variant	*SLC35D2*	rs142809659
Twin_A	9	101191229	G	A	Substitution	intron_variant	*PLPPR1*	-
Twin_A	9	101784275	G	A	Substitution	intergenic_variant	-	-
Twin_A	9	102487319	C	T	Substitution	intergenic_variant	-	rs1189198082
Twin_A	9	111554985	G	T	Substitution	3_prime_UTR_variant	*ZNF483*	rs1039810650
Twin_A	9	118247951	T	C	Substitution	intergenic_variant	-	-
Twin_A	9	120700338	T	A	Substitution	intron_variant	*MEGF9*	-
Twin_A	9	122872477	T	C	Substitution	downstream_gene_variant	*RC3H2*	rs996687988
Twin_A	9	125539853	T	TA	Insertion	intron_variant	*MAPKAP1*	rs201724260
Twin_A	9	127663224	C	G	Substitution	missense_variant	*STXBP1*	-
Twin_A	9	131190221	A	T	Substitution	intron_variant	*NUP214*	-
Twin_A	9	134828811	CCA	C	Deletion	intron_variant	*COL5A1*	rs906680873
Twin_A	9	136468856	C	CT	Insertion	intron_variant	*SEC16A*	-
Twin_A	10	4183898	A	G	Substitution	regulatory_region_variant	-	-
Twin_A	10	9239651	C	CT	Insertion	intergenic_variant	-	rs963757968
Twin_A	10	10849842	T	A	Substitution	intron_variant	*CELF2*	rs1365457744
Twin_A	10	11168124	TTC	T	Deletion	intron_variant	*CELF2*	-
Twin_A	10	15299120	C	T	Substitution	intron_variant	*FAM171A1*	rs558867054
Twin_A	10	18976904	C	T	Substitution	intergenic_variant	-	rs181225799
Twin_A	10	23617158	C	CAT	Insertion	intergenic_variant	-	rs144728449
Twin_A	10	23958376	G	A	Substitution	intron_variant	*KIAA1217*	-
Twin_A	10	27864409	G	GA	Insertion	intron_variant	*ARMC4*	rs998440262
Twin_A	10	34892061	G	T	Substitution	intergenic_variant	-	-
Twin_A	10	47221434	C	T	Substitution	intergenic_variant	-	-
Twin_A	10	54174376	C	CA	Insertion	intron_variant	*PCDH15*	rs112450699
Twin_A	10	56140794	C	CAT	Insertion	intergenic_variant	-	rs999676086
Twin_A	10	56950504	A	T	Substitution	intergenic_variant	-	-
Twin_A	10	60147699	C	G	Substitution	intron_variant	*ANK3*	-
Twin_A	10	62705771	AAT	A	Deletion	intron_variant	*AC067751.1*	rs10573238
Twin_A	10	64210270	G	T	Substitution	intron_variant	*AC013287.1*	rs75029261
Twin_A	10	67571470	TA	T	Deletion	intron_variant	*CTNNA3*	-
Twin_A	10	70021310	G	GA	Insertion	upstream_gene_variant	*LINC02636*	rs944814046

Twin_A	10	75905930	G	GT	Insertion	intron_variant	*LRMDA*	rs5786198
Twin_A	10	78393818	G	A	Substitution	intron_variant	*AC012560.1*	-
Twin_A	10	83188578	G	A	Substitution	intergenic_variant	-	rs778523013
Twin_A	10	83492892	C	T	Substitution	intergenic_variant	-	-
Twin_A	10	93187731	G	A	Substitution	intergenic_variant	-	-
Twin_A	10	94576898	A	AT	Insertion	intron_variant	*HELLS*	rs540746312
Twin_A	10	94950682	A	G	Substitution	intron_variant	*CYP2C9*	rs907386662
Twin_A	10	103273122	G	A	Substitution	upstream_gene_variant	*INA*	rs1286474689
Twin_A	10	109091857	G	A	Substitution	intergenic_variant	-	rs954171718
Twin_A	10	111830308	A	G	Substitution	intron_variant	*AL136119.1*	rs1430607514
Twin_A	10	113814958	A	G	Substitution	intergenic_variant	-	-
Twin_A	10	115169017	CTT	C	Deletion	intron_variant	*ATRNL1*	rs71010011
Twin_A	10	115409272	G	A	Substitution	intron_variant	*ATRNL1*	-
Twin_A	10	116526803	T	C	Substitution	intergenic_variant	-	-
Twin_A	10	120224348	T	TA	Insertion	intergenic_variant	-	-
Twin_A	10	121613197	T	C	Substitution	downstream_gene_variant	*AC009988.1*	-
Twin_A	10	122518538	G	A	Substitution	downstream_gene_variant	*HTRA1*	rs568255913
Twin_A	10	124140841	T	C	Substitution	intergenic_variant	-	rs1455378017
Twin_A	10	124588678	T	G	Substitution	intron_variant	*LHPP*	-
Twin_A	11	6802600	C	T	Substitution	upstream_gene_variant	*OR6A2*	rs745932107
Twin_A	11	12635917	G	A	Substitution	intron_variant	*AC107881.1*	rs1470450176
Twin_A	11	17634218	C	T	Substitution	stop_gained	*OTOG*	rs866476223
Twin_A	11	18214790	G	A	Substitution	downstream_gene_variant	*SLC25A51P4*	-
Twin_A	11	25629284	G	T	Substitution	upstream_gene_variant	*AC100770.1*	-
Twin_A	11	27431935	C	T	Substitution	intron_variant	*LGR4*	rs187542892
Twin_A	11	29268317	G	GTA	Insertion	downstream_gene_variant	*AC090791.1*	rs57529313
Twin_A	11	30414572	A	AT	Insertion	intron_variant	*MPPED2*	rs574457624
Twin_A	11	34947336	TAC	T	Deletion	intron_variant	*PDHX*	rs201136678
Twin_A	11	40970112	A	T	Substitution	intron_variant	*LRRC4C*	-
Twin_A	11	42181489	A	AT	Insertion	downstream_gene_variant	*LINC02740*	rs1268779757
Twin_A	11	42992114	T	A	Substitution	intergenic_variant	-	-
Twin_A	11	49443145	TATC	T	Deletion	intergenic_variant	-	rs1327126227
Twin_A	11	59547928	A	G	Substitution	intron_variant	*AP003778.1*	-
Twin_A	11	62907973	C	T	Substitution	downstream_gene_variant	*M1*	-
Twin_A	11	69424352	G	A	Substitution	upstream_gene_variant	*AP000439.2*	-
Twin_A	11	70172132	A	G	Substitution	intron_variant	*ANO1*	rs11235475
Twin_A	11	71917009	GTTA	G	Deletion	downstream_gene_variant	*AP002495.1*	rs550657033
Twin_A	11	72630002	T	A	Substitution	intron_variant	*PDE2A*	rs956005801
Twin_A	11	78991748	C	T	Substitution	intron_variant	*TENM4*	-
Twin_A	11	83859358	A	G	Substitution	intron_variant	*DLG2*	-
Twin_A	11	83984865	G	A	Substitution	intron_variant	*DLG2*	rs2514172
Twin_A	11	87084313	C	CT	Insertion	intron_variant	*TMEM135*	rs5793234

Twin_A	11	89364256	TTC	T	Deletion	intron_variant	*NOX4*	-
Twin_A	11	91531418	GTC	G	Deletion	intergenic_variant	-	rs57259378
Twin_A	11	92625315	G	A	Substitution	intron_variant	*FAT3*	rs1044839433
Twin_A	11	93542010	C	T	Substitution	intron_variant	*SMCO4*	rs1238441458
Twin_A	11	93572399	A	T	Substitution	regulatory_region_variant	-	-
Twin_A	11	96395662	G	GA	Insertion	downstream_gene_variant	*JRKL*	rs1197147759
Twin_A	11	98979001	G	GCA	Insertion	intergenic_variant	-	rs5793962
Twin_A	11	99123208	G	GT	Insertion	downstream_gene_variant	*RN7SKP53*	rs370959291
Twin_A	11	105917523	A	G	Substitution	intron_variant	*GRIA4*	-
Twin_A	11	112108171	G	T	Substitution	intron_variant	*AP002884.2*	-
Twin_A	11	118973448	G	T	Substitution	intron_variant	*FOXR1*	-
Twin_A	11	121731425	T	C	Substitution	intron_variant	*AP001977.1*	-
Twin_A	11	130155838	CA	C	Deletion	upstream_gene_variant	*ST14*	rs71061368
Twin_A	11	133629356	A	T	Substitution	intergenic_variant	-	-
Twin_A	12	8992708	A	AT	Insertion	intron_variant	*KLRG1*	rs1011939413
Twin_A	12	14002642	GTA	G	Deletion	intergenic_variant	-	rs767637242
Twin_A	12	27135862	C	A	Substitution	intron_variant	*AC092747.1*	-
Twin_A	12	33793906	A	G	Substitution	intergenic_variant	-	rs1160899620
Twin_A	12	37544249	GGT	G	Deletion	intergenic_variant	-	rs139856566
Twin_A	12	39556427	T	A	Substitution	intron_variant	*ABCD2*	-
Twin_A	12	45801804	CAG	C	Deletion	intron_variant	*ARID2*	rs200185178
Twin_A	12	47093368	A	AT	Insertion	intron_variant	*PCED1B*	rs142397807
Twin_A	12	54193118	A	G	Substitution	upstream_gene_variant	*SMUG1*	-
Twin_A	12	55987166	G	GT	Insertion	intron_variant	*RAB5B*	rs34223637
Twin_A	12	61094238	T	C	Substitution	intergenic_variant	-	rs795318
Twin_A	12	61096059	C	T	Substitution	intergenic_variant	-	-
Twin_A	12	63499491	A	T	Substitution	intergenic_variant	-	rs367633111
Twin_A	12	67241412	G	GA	Insertion	intergenic_variant	-	rs570864048
Twin_A	12	80465550	CA	C	Deletion	intron_variant	*PTPRQ*	rs1565723030
Twin_A	12	96021004	T	A	Substitution	intron_variant	*LTA4H*	-
Twin_A	12	99195605	T	C	Substitution	intron_variant	*ANKS1B*	-
Twin_A	12	99570645	C	CA	Insertion	intron_variant	*ANKS1B*	rs34453262
Twin_A	12	100385137	A	T	Substitution	intron_variant	*SLC17A8*	rs564312355
Twin_A	12	101478301	G	A	Substitution	intron_variant	*SPIC*	-
Twin_A	12	111703138	A	G	Substitution	intron_variant	*ACAD10*	-
Twin_A	12	112201473	G	GA	Insertion	intron_variant	*HECTD4*	rs59104653
Twin_A	12	117866839	T	TA	Insertion	intron_variant	*KSR2*	rs34463256
Twin_A	12	120238552	T	A	Substitution	intron_variant	*PXN*	-
Twin_A	12	129194036	A	G	Substitution	intron_variant	*TMEM132D*	-
Twin_A	12	129699976	C	T	Substitution	missense_variant	*TMEM132D*	COSV70606773
Twin_A	12	130201343	C	T	Substitution	intergenic_variant	-	rs752475595
Twin_A	12	131372998	C	T	Substitution	downstream_gene_variant	*AC140118.1*	rs1044089851

Twin_A	12	131449745	T	TTATAT ATATAT A	Insertion	intron_variant	*AC073578.2*	rs60625980
Twin_A	12	131913217	G	T	Substitution	synonymous_variant	*ULK1*	COSV58854885
Twin_A	12	132658754	CG	C	Deletion	intron_variant	*POLE*	-
Twin_A	13	16312671	T	G	Substitution	intergenic_variant	-	rs1452678559
Twin_A	13	19458900	T	C	Substitution	intron_variant	*TPTE2*	-
Twin_A	13	21198801	T	A	Substitution	regulatory_region_variant	-	-
Twin_A	13	21198803	G	T	Substitution	regulatory_region_variant	-	-
Twin_A	13	22486341	G	A	Substitution	intergenic_variant	-	-
Twin_A	13	24407230	C	T	Substitution	upstream_gene_variant	*TPTE2P6*	-
Twin_A	13	27747684	T	C	Substitution	downstream_gene_variant	*POLR1D*	rs530116991
Twin_A	13	33789922	C	T	Substitution	regulatory_region_variant	-	-
Twin_A	13	35458160	C	G	Substitution	intron_variant	*NBEA*	-
Twin_A	13	40248330	G	A	Substitution	intron_variant	*LINC00598*	rs570712758
Twin_A	13	44807574	G	A	Substitution	intron_variant	*LINC00330*	-
Twin_A	13	51185115	A	T	Substitution	intron_variant	*C13orf42*	rs982603544
Twin_A	13	54356322	T	A	Substitution	regulatory_region_variant	-	-
Twin_A	13	57950093	C	T	Substitution	intergenic_variant	-	-
Twin_A	13	60127004	G	A	Substitution	intron_variant	*DIAPH3*	-
Twin_A	13	61979712	AT	A	Deletion	intergenic_variant	-	-
Twin_A	13	86387551	T	TA	Insertion	intergenic_variant	-	rs578195336
Twin_A	13	95408931	G	T	Substitution	intergenic_variant	-	-
Twin_A	13	97673189	G	A	Substitution	downstream_gene_variant	*AL359502.1*	rs1006927199
Twin_A	13	100032713	C	CA	Insertion	upstream_gene_variant	*NDUFA12P1*	rs914603334
Twin_A	13	110038016	G	GCA	Insertion	intron_variant	*AL390755.1*	rs34470615
Twin_A	13	112177050	A	T	Substitution	intergenic_variant	-	-
Twin_A	13	112393758	C	T	Substitution	intron_variant	*SPACA7*	-
Twin_A	14	27812525	AAG	A	Deletion	intron_variant	*AL390334.1*	rs368944583
Twin_A	14	27980040	G	A	Substitution	intergenic_variant	-	-
Twin_A	14	29729980	G	A	Substitution	intron_variant	*PRKD1*	rs536812458
Twin_A	14	30110595	C	CT	Insertion	intron_variant	*PRKD1*	rs1412349193
Twin_A	14	37141508	T	TA	Insertion	intron_variant	*SLC25A21*	rs533050099
Twin_A	14	39514017	ATATC	A	Deletion	downstream_gene_variant	*AL049828.1*	rs78432958
Twin_A	14	58577605	T	A	Substitution	intergenic_variant	-	-
Twin_A	14	59907903	T	C	Substitution	intergenic_variant	-	-
Twin_A	14	59942776	C	CT	Insertion	intron_variant	*LRRC9*	rs1440781193
Twin_A	14	61869166	A	G	Substitution	intron_variant	*SYT16*	-
Twin_A	14	81424362	TG	T	Deletion	intron_variant	*STON2*	-
Twin_A	14	87211954	G	GA	Insertion	regulatory_region_variant	-	-
Twin_A	15	17735761	T	G	Substitution	intergenic_variant	-	rs1425932497
Twin_A	15	24574554	C	A	Substitution	non_coding_transcript_ exon_variant	*PWRN1*	-
Twin_A	15	25170693	A	G	Substitution	upstream_gene_variant	*SNORD115*	-

Twin_A	15	27699074	C	T	Substitution	intergenic_variant	-	rs979345355
Twin_A	15	27798227	G	A	Substitution	intron_variant	*OCA2*	rs575593376
Twin_A	15	56330772	G	GA	Insertion	intron_variant	*TEX9*	rs202192979
Twin_A	15	58918915	C	CT	Insertion	intron_variant	*SLTM*	rs566389959
Twin_A	15	60956234	G	GT	Insertion	intron_variant	*RORA*	rs796500717
Twin_A	15	63420915	CAT	C	Deletion	intron_variant	*LINC02568*	rs142303229
Twin_A	15	69253960	G	A	Substitution	intron_variant	*GLCE*	-
Twin_A	15	80950475	T	A	Substitution	3_prime_UTR_variant	*CEMIP*	-
Twin_A	15	83158851	C	CT	Insertion	intron_variant	*HDGFL3*	rs554737989
Twin_A	15	96972773	A	G	Substitution	intergenic_variant	-	-
Twin_A	15	99025033	T	A	Substitution	intron_variant	*AC036108.1*	-
Twin_A	16	3192939	A	AT	Insertion	intron_variant	*AJ003147.2*	-
Twin_A	16	7113874	T	C	Substitution	intron_variant	*RBFOX1*	-
Twin_A	16	7450654	G	A	Substitution	intron_variant	*RBFOX1*	rs146577787
Twin_A	16	7806761	G	A	Substitution	intergenic_variant	-	rs752635066
Twin_A	16	8247620	G	A	Substitution	intergenic_variant	-	rs942245726
Twin_A	16	8372617	C	T	Substitution	intron_variant	*AC018767.1*	rs909464601
Twin_A	16	11338624	C	CT	Insertion	intron_variant	*RMI2*	rs377660326
Twin_A	16	13671261	G	A	Substitution	intergenic_variant	-	rs544425045
Twin_A	16	22306291	C	T	Substitution	intron_variant	*POLR3E*	-
Twin_A	16	25681378	C	T	Substitution	intergenic_variant	-	-
Twin_A	16	34085953	C	T	Substitution	regulatory_region_variant	-	rs759751179
Twin_A	16	35232723	C	A	Substitution	intron_variant	*AC023824.3*	-
Twin_A	16	35736091	G	A	Substitution	downstream_gene_variant	*RNA5SP407*	rs1168416095
Twin_A	16	47099927	C	T	Substitution	intron_variant	*NETO2*	-
Twin_A	16	47823047	C	A	Substitution	intron_variant	*LINC02133*	-
Twin_A	16	48066801	G	T	Substitution	intergenic_variant	-	-
Twin_A	16	49303993	C	T	Substitution	intron_variant	*AC007614.1*	-
Twin_A	16	49413774	A	C	Substitution	regulatory_region_variant	-	-
Twin_A	16	51823583	C	T	Substitution	intergenic_variant	-	-
Twin_A	16	52276870	G	A	Substitution	intron_variant	*CASC22*	-
Twin_A	16	52638073	C	T	Substitution	intron_variant	*CASC16*	rs995891657
Twin_A	16	61886296	T	TA	Insertion	intron_variant	*CDH8*	rs535315018
Twin_A	16	64432341	G	GT	Insertion	intron_variant	*AC092131.1*	rs901796829
Twin_A	16	64614439	G	A	Substitution	intergenic_variant	-	-
Twin_A	16	64919655	T	A	Substitution	intergenic_variant	-	-
Twin_A	16	66602481	T	TA	Insertion	upstream_gene_variant	*CMTM3*	rs145668319
Twin_A	16	67608941	A	G	Substitution	intron_variant	*CTCF*	rs892105462
Twin_A	16	71030715	G	C	Substitution	intron_variant	*HYDIN*	rs12102892
Twin_A	16	72362589	G	T	Substitution	intron_variant	*LINC01572*	-
Twin_A	16	74358477	G	GTGTGT GTGTA	Insertion	intron_variant	*AC009053.1*	rs1355695440
Twin_A	16	77781037	CAGG	C	Deletion	regulatory_region_variant	-	rs145741815

Twin_A	16	83070380	C	T	Substitution	intron_variant	*CDH13*	-
Twin_A	16	85313584	C	A	Substitution	intron_variant	*GSE1*	rs1483404740
Twin_A	16	86284090	G	A	Substitution	intron_variant	*LINC01081*	-
Twin_A	17	578020	G	C	Substitution	intron_variant	*VPS53*	-
Twin_A	17	2793153	AT	A	Deletion	upstream_gene_variant	*RAP1GAP2*	rs200252910
Twin_A	17	8040550	GA	G	Deletion	intron_variant	*ALOX15B*	rs57248868
Twin_A	17	8080028	C	T	Substitution	non_coding_transcript_ exon_variant	*AC129492.1*	-
Twin_A	17	9019997	A	G	Substitution	upstream_gene_variant	*NTN1*	-
Twin_A	17	9546387	TA	T	Deletion	intron_variant	*STX8*	rs56901611
Twin_A	17	14728731	G	A	Substitution	intron_variant	*AC013248.1*	-
Twin_A	17	15719138	C	CA	Insertion	3_prime_UTR_variant	*ZNF286A*	rs372793188
Twin_A	17	32918315	T	G	Substitution	regulatory_region_variant	-	-
Twin_A	17	34590397	T	C	Substitution	intron_variant	*TMEM132E*	rs1390883369
Twin_A	17	34849723	CACA	C	Deletion	intron_variant	*AC022903.1*	rs952016458
Twin_A	17	41287213	A	G	Substitution	regulatory_region_variant	-	-
Twin_A	17	48780087	G	GA	Insertion	intron_variant	*TTLL6*	rs371091415
Twin_A	17	63184014	C	CA	Insertion	intron_variant	*TANC2*	rs570141267
Twin_A	17	64353316	C	T	Substitution	intron_variant	*PECAM1*	rs879198491
Twin_A	17	66569297	G	A	Substitution	intron_variant	*PRKCA*	rs540966408
Twin_A	17	72065475	G	A	Substitution	intron_variant	*AC007461.1*	-
Twin_A	17	72109757	C	T	Substitution	intron_variant, non_coding_transcript_variant	*SOX9-AS1*	-
Twin_A	17	73729002	C	G	Substitution	intergenic_variant	-	-
Twin_A	17	78164605	G	A	Substitution	upstream_gene_variant	*SYNGR2*	-
Twin_A	17	79118078	G	A	Substitution	intron_variant	*RBFOX3*	rs560513487
Twin_A	17	82853402	C	CT	Insertion	intron_variant	*TBCD*	-
Twin_A	18	9601220	A	G	Substitution	intron_variant	*PPP4R1*	rs982592192
Twin_A	18	14227264	CAA	C	Deletion	intron_variant	*ANKRD20A5P*	rs200661335
Twin_A	18	20189631	G	A	Substitution	intergenic_variant	-	rs1337042912
Twin_A	18	20830406	G	A	Substitution	intergenic_variant	-	-
Twin_A	18	27867692	T	C	Substitution	intergenic_variant	-	-
Twin_A	18	29896712	C	T	Substitution	intergenic_variant	-	-
Twin_A	18	32161626	G	T	Substitution	intron_variant	*GAREM1*	-
Twin_A	18	32191812	T	C	Substitution	intron_variant	*MEP1B*	-
Twin_A	18	33837694	C	A	Substitution	regulatory_region_variant	-	-
Twin_A	18	34514791	G	A	Substitution	intron_variant	*DTNA*	-
Twin_A	18	41122265	A	G	Substitution	intergenic_variant	-	-
Twin_A	18	50652311	G	A	Substitution	intron_variant	*MAPK4*	-
Twin_A	18	51531692	G	A	Substitution	intron_variant	*LINC01630*	-
Twin_A	18	52758063	C	T	Substitution	intron_variant	*DCC*	rs75634824
Twin_A	18	53231840	T	C	Substitution	intron_variant	*DCC*	rs1236652956
Twin_A	18	56463814	T	TA	Insertion	intergenic_variant	-	rs139733893
Twin_A	18	57470379	T	C	Substitution	intron_variant	*ONECUT2*	-

Twin_A	18	63454630	A	AT	Insertion	regulatory_region_variant	-	rs900204433
Twin_A	18	68909930	A	T	Substitution	intron_variant	*CCDC102B*	-
Twin_A	18	70340966	C	G	Substitution	upstream_gene_variant	*LIVAR*	-
Twin_A	18	75784106	G	A	Substitution	intergenic_variant	-	-
Twin_A	18	76850613	G	GT	Insertion	intron_variant	*ZNF236*	-
Twin_A	19	3661315	C	A	Substitution	intron_variant	*PIP5K1C*	-
Twin_A	19	7346680	T	C	Substitution	upstream_gene_variant	*AC119396.1*	-
Twin_A	19	10065329	CAA	C	Deletion	intron_variant	*C3P1*	-
Twin_A	19	12943750	AGCAGAG GCTTAAG GAGGAGG AAGAAGA CAAGAAA CGCAAAG AGGAGGA GGAG	A	Deletion	frameshift_variant	*CALR*	rs1555760738
Twin_A	19	14455965	T	TA	Insertion	intron_variant	*PKN1*	rs540413655
Twin_A	19	15471572	G	C	Substitution	intron_variant	*PGLYRP2*	-
Twin_A	19	16234332	C	T	Substitution	intron_variant	*AP1M1*	rs1266106157
Twin_A	19	22883084	C	T	Substitution	regulatory_region_variant	-	-
Twin_A	19	22892036	CT	C	Deletion	intergenic_variant	-	rs59650600
Twin_A	19	27404653	C	G	Substitution	intergenic_variant	-	-
Twin_A	19	27753864	G	T	Substitution	downstream_gene_variant	*AC006504.1*	-
Twin_A	19	28326785	C	T	Substitution	intron_variant	*AC005580.1*	rs1436772262
Twin_A	19	28434046	C	T	Substitution	upstream_gene_variant	*AC005307.1*	rs1029855214
Twin_A	19	40522348	A	T	Substitution	intron_variant	*SPTBN4*	rs1434639441
Twin_A	19	44493750	T	TCTCTC TTG	Insertion	intron_variant	*ZNF180*	rs1258057463
Twin_A	19	48040435	C	A	Substitution	intron_variant	*CABP5*	-
Twin_A	19	54956200	CAAGG	C	Deletion	intron_variant	*NLRP7*	rs752034667
Twin_A	19	55462076	G	GGA	Insertion	upstream_gene_variant	*ISOC2*	-
Twin_A	20	4322280	G	GT	Insertion	regulatory_region_variant	-	rs921737367
Twin_A	20	13915926	C	G	Substitution	intron_variant	*SEL1L2*	rs1392345509
Twin_A	20	24530811	G	GT	Insertion	intron_variant	*SYNDIG1*	rs374193810
Twin_A	20	28628245	A	G	Substitution	intergenic_variant	-	rs879967418
Twin_A	20	39766967	C	T	Substitution	intron_variant	*AL118523.1*	rs1468797562
Twin_A	20	42620323	G	C	Substitution	intron_variant	*PTPRT*	-
Twin_A	20	44696207	C	A	Substitution	upstream_gene_variant	*AL139352.1*	-
Twin_A	20	45737606	A	T	Substitution	intergenic_variant	-	-
Twin_A	20	51168664	C	A	Substitution	intergenic_variant	-	-
Twin_A	20	53076039	C	T	Substitution	intron_variant	*TSHZ2*	rs559693937
Twin_A	20	55145960	T	C	Substitution	intergenic_variant	-	-
Twin_A	20	55476594	G	A	Substitution	intron_variant	*LINC01440*	rs910371781
Twin_A	20	57038323	C	T	Substitution	intergenic_variant	-	-
Twin_A	21	10536357	C	T	Substitution	intron_variant	*TPTE*	-
Twin_A	21	18807398	GCA	G	Deletion	intergenic_variant	-	rs56088107
Twin_A	21	24361606	G	GT	Insertion	intron_variant	*LINC01684*	rs564109768

Twin_A	21	25056551	G	A	Substitution	intron_variant	*AP000235.1*	rs529745996
Twin_A	21	25235168	GTTT	G	Deletion	intron_variant	*AP001341.1*	-
Twin_A	21	26121394	C	CT	Insertion	intron_variant	*APP*	rs147930861
Twin_A	21	27842645	T	C	Substitution	intergenic_variant	-	-
Twin_A	21	28932587	T	C	Substitution	synonymous_variant	*LTN1*	-
Twin_A	21	31309695	A	G	Substitution	intron_variant	*TIAM1*	-
Twin_A	21	32999596	A	T	Substitution	intron_variant	*AP000282.1*	rs1265368759
Twin_A	21	35313715	G	A	Substitution	intron_variant	*RUNX1*	rs1050527217
Twin_A	21	35666262	C	T	Substitution	intron_variant	*RUNX1*	-
Twin_A	21	37443516	T	G	Substitution	intron_variant	*DYRK1A*	rs575428175
Twin_A	21	41859981	G	T	Substitution	intron_variant	*PRDM15*	-
Twin_A	21	42078747	A	G	Substitution	intron_variant	*UMODL1*	-
Twin_A	22	12521506	A	T	Substitution	intergenic_variant	-	rs1476432335
Twin_A	22	15748369	C	T	Substitution	downstream_gene_variant	*GRAMD4P2*	rs529661114
Twin_A	22	16418819	G	A	Substitution	intergenic_variant	-	rs73387994
Twin_A	22	20582589	G	A	Substitution	intron_variant	*MED15*	rs765884898
Twin_A	22	30118881	G	A	Substitution	intron_variant	*HORMAD2*	-
Twin_A	22	33353441	A	G	Substitution	intron_variant	*LARGE1*	rs1007665809
Twin_A	22	34641670	G	A	Substitution	non_coding_transcript_ exon_variant	*AL021877.2*	rs765266284
Twin_A	22	37322215	G	A	Substitution	intergenic_variant	-	rs898967364
Twin_A	22	44313831	C	T	Substitution	upstream_gene_variant	*SHISAL1*	-
Twin_A	22	45732457	C	CA	Insertion	intron_variant	*ATXN10*	rs971974596
Twin_A	22	50478755	C	G	Substitution	upstream_gene_variant	*SBF1*	-
Twin_A	X	1688073	G	A	Substitution	intergenic_variant	-	rs1402629241
Twin_A	X	3810086	TAC	T	Deletion	intergenic_variant	-	rs768564761
Twin_A	X	9793750	C	CT	Insertion	intron_variant	*SHROOM2*	rs368389307
Twin_A	X	10753565	T	TG	Insertion	intron_variant	*MID1*	rs745425008
Twin_A	X	20062481	T	TA	Insertion	intron_variant	*MAP7D2*	rs141341947
Twin_A	X	21515041	T	TA	Insertion	intron_variant	*CNKSR2*	rs111880925
Twin_A	X	26568200	C	T	Substitution	intron_variant	*VENTXP1*	rs760685569
Twin_A	X	35432699	T	C	Substitution	intergenic_variant	-	-
Twin_A	X	41283486	G	T	Substitution	intergenic_variant	-	-
Twin_A	X	43355581	AAG	A	Deletion	intron_variant	*AL023574.1*	rs747346757
Twin_A	X	48123449	G	A	Substitution	downstream_gene_variant	*SSX6P*	rs1311655959
Twin_A	X	53838712	T	TTTTTT C	Insertion	intergenic_variant	-	rs1491090843
Twin_A	X	56963616	T	TCA	Insertion	intron_variant	*SPIN3*	rs367803469
Twin_A	X	64210261	C	CA	Insertion	upstream_gene_variant	*AMER1*	rs1361525685
Twin_A	X	66930421	A	T	Substitution	intergenic_variant	-	-
Twin_A	X	69456838	G	GT	Insertion	intergenic_variant	-	rs367604349
Twin_A	X	73938408	AAAAT	A	Deletion	downstream_gene_variant	*FTX*	rs1467359046
Twin_A	X	74173149	C	CA	Insertion	upstream_gene_variant	*ATP5MDP1*	rs1215712048
Twin_A	X	80107643	A	T	Substitution	intergenic_variant	-	-

Twin_A	X	102759593	AAC	A	Deletion	intergenic_variant	-	rs4009667
Twin_A	X	105950823	CAG	C	Deletion	intron_variant	*NRK*	rs746466025
Twin_A	X	113523343	C	G	Substitution	downstream_gene_variant	*AC233289.1*	-
Twin_A	X	114062259	G	GT	Insertion	upstream_gene_variant	*XACT*	rs1357977704
Twin_A	X	115465393	T	C	Substitution	intergenic_variant	-	-
Twin_A	X	119227169	C	CA	Insertion	upstream_gene_variant	*AC004835.2*	rs11420910
Twin_A	X	130938304	T	C	Substitution	intergenic_variant	-	-
Twin_A	X	142708225	C	A	Substitution	intron_variant	*AL031073.2*	-
Twin_A	X	144608023	C	T	Substitution	intergenic_variant	-	-
Twin_A	Y	12123479	G	GA	Insertion	intron_variant	*GYG2P1*	rs113188804
Twin_A	Y	20863824	C	T	Substitution	downstream_gene_variant	*GAPDHP17*	-
Twin_B	1	1948293	G	A	Substitution	intron_variant	*CFAP74*	-
Twin_B	1	5360980	A	G	Substitution	intergenic_variant	-	-
Twin_B	1	7752725	A	G	Substitution	intron_variant	*CAMTA1*	rs1558304741
Twin_B	1	8034377	T	C	Substitution	intron_variant	*AL034417.2*	-
Twin_B	1	12823937	C	T	Substitution	downstream_gene_variant	*PRAMEF11*	-
Twin_B	1	16874999	G	T	Substitution	downstream_gene_variant	*BX284668.2*	rs6586499
Twin_B	1	21195179	C	T	Substitution	TF_binding_site_variant	*AL023574.1*	rs1040263005
Twin_B	1	28903853	G	GT	Insertion	intron_variant	*EPB41*	rs776964978
Twin_B	1	30198944	G	A	Substitution	intergenic_variant	-	rs959100631
Twin_B	1	46567799	T	A	Substitution	downstream_gene_variant	*MKNK1*	-
Twin_B	1	46733432	G	A	Substitution	intergenic_variant	-	rs910046833
Twin_B	1	46925759	G	C	Substitution	downstream_gene_variant	*CYP4A11*	-
Twin_B	1	47112037	C	T	Substitution	intron_variant	*CYP4Z1*	rs764020558
Twin_B	1	49651607	C	T	Substitution	intron_variant	*AGBL4*	-
Twin_B	1	52446089	C	T	Substitution	downstream_gene_variant	*TUT4*	-
Twin_B	1	55508033	CA	C	Deletion	intron_variant	*AL603840.1*	-
Twin_B	1	57203495	C	T	Substitution	intron_variant	*DAB1*	-
Twin_B	1	68690432	C	T	Substitution	intron_variant	*AL033530.1*	rs1263200065
Twin_B	1	90494264	G	A	Substitution	intergenic_variant	-	-
Twin_B	1	104033383	A	G	Substitution	intergenic_variant	-	-
Twin_B	1	110190300	G	C	Substitution	intron_variant	*SLC6A17*	-
Twin_B	1	110738023	A	G	Substitution	intergenic_variant	-	-
Twin_B	1	114307529	G	C	Substitution	intergenic_variant	-	-
Twin_B	1	115892616	C	T	Substitution	intergenic_variant	-	rs1302223823
Twin_B	1	143898748	G	A	Substitution	downstream_gene_variant	*H3*	-
Twin_B	1	147277076	C	A	Substitution	downstream_gene_variant	*CHD1L*	-
Twin_B	1	157062690	T	C	Substitution	upstream_gene_variant	*SMU1P1*	-
Twin_B	1	158329018	G	A	Substitution	splice_region_variant	*CD1B*	-
Twin_B	1	159742120	G	T	Substitution	intergenic_variant	-	-
Twin_B	1	165976313	A	G	Substitution	intergenic_variant	-	-
Twin_B	1	166242296	C	T	Substitution	intron_variant	*AL596087.2*	-

Twin_B	1	166972081	G	A	Substitution	intron_variant	*ILDR2*	rs747200728
Twin_B	1	170852137	G	C	Substitution	intron_variant	*BX284613.2*	-
Twin_B	1	175165067	G	T	Substitution	intron_variant	*KIAA0040*	-
Twin_B	1	176214397	G	A	Substitution	intron_variant	*AL591043.2*	-
Twin_B	1	177128801	C	A	Substitution	intron_variant	*ASTN1*	-
Twin_B	1	187326523	G	A	Substitution	intron_variant	*LINC01036*	-
Twin_B	1	190010459	C	T	Substitution	intergenic_variant	-	rs574701171
Twin_B	1	192061801	C	A	Substitution	intergenic_variant	-	-
Twin_B	1	194370180	G	A	Substitution	intergenic_variant	-	rs568526830
Twin_B	1	194805882	A	T	Substitution	intron_variant	*AL353072.2*	rs529354380
Twin_B	1	197898293	C	A	Substitution	upstream_gene_variant	*C1orf53*	-
Twin_B	1	211364253	C	T	Substitution	downstream_gene_variant	*TRAF5*	-
Twin_B	1	213739818	C	T	Substitution	intron_variant	*AC096639.1*	rs751851426
Twin_B	1	216808720	G	A	Substitution	intron_variant	*ESRRG*	-
Twin_B	1	226114304	T	TTA	Insertion	intergenic_variant	-	rs1263797763
Twin_B	1	228759153	C	T	Substitution	intergenic_variant	-	-
Twin_B	1	231661954	G	T	Substitution	intron_variant	*DISC1*	-
Twin_B	1	237523679	G	A	Substitution	intron_variant	*RYR2*	rs1165384474
Twin_B	1	239287292	C	T	Substitution	intergenic_variant	-	-
Twin_B	1	239790569	T	G	Substitution	intron_variant	*CHRM3*	-
Twin_B	2	3059701	C	T	Substitution	intron_variant	*LINC01250*	rs902349418
Twin_B	2	5809965	G	A	Substitution	downstream_gene_variant	*LINC01810*	-
Twin_B	2	7937210	A	T	Substitution	intron_variant	*LINC00298*	-
Twin_B	2	14870316	G	A	Substitution	intron_variant	*AC068286.2*	-
Twin_B	2	17702798	C	A	Substitution	downstream_gene_variant	*SMC6*	rs956379181
Twin_B	2	17816780	A	G	Substitution	missense_variant	*MSGN1*	-
Twin_B	2	21701231	C	T	Substitution	intron_variant	*AC018742.1*	-
Twin_B	2	22021980	T	C	Substitution	intron_variant	*AC096570.1*	-
Twin_B	2	22585137	GT	G	Deletion	intergenic_variant	-	-
Twin_B	2	22585139	T	A	Substitution	intergenic_variant	-	-
Twin_B	2	22619775	C	T	Substitution	intergenic_variant	-	rs540314768
Twin_B	2	22994571	A	G	Substitution	intergenic_variant	-	rs1455047191
Twin_B	2	24019295	T	C	Substitution	downstream_gene_variant	*MFSD2B*	-
Twin_B	2	27314362	G	A	Substitution	downstream_gene_variant	*MPV17*	-
Twin_B	2	28287298	T	A	Substitution	intron_variant	*BABAM2*	-
Twin_B	2	29298533	A	G	Substitution	intron_variant	*ALK*	-
Twin_B	2	30708062	G	A	Substitution	intergenic_variant	-	rs1306752997
Twin_B	2	33204005	TG	T	Deletion	intron_variant	*LTBP1*	-
Twin_B	2	34609950	A	G	Substitution	intron_variant	*LINC01320*	-
Twin_B	2	35621513	C	T	Substitution	intergenic_variant	-	rs185192135
Twin_B	2	39543295	C	T	Substitution	intron_variant	*MAP4K3*	-
Twin_B	2	41851787	C	T	Substitution	regulatory_region_variant	-	-

Twin_B	2	52199227	T	C	Substitution	intron_variant	*AC007402.1*	-
Twin_B	2	55637956	C	T	Substitution	intron_variant	*PNPT1*	rs931753562
Twin_B	2	55778474	G	A	Substitution	intergenic_variant	-	rs1269019834
Twin_B	2	59742006	A	T	Substitution	intron_variant	*AC007100.1*	-
Twin_B	2	65862748	C	T	Substitution	intron_variant	*AC007389.1*	-
Twin_B	2	67673061	AGC	A	Deletion	intron_variant	*AC007422.1*	rs142178881
Twin_B	2	72017331	C	T	Substitution	intergenic_variant	-	-
Twin_B	2	76343534	G	A	Substitution	intron_variant	*AC073091.1*	-
Twin_B	2	77138659	C	T	Substitution	intron_variant	*LRRTM4*	-
Twin_B	2	77305966	T	C	Substitution	intron_variant	*LRRTM4*	rs1354282051
Twin_B	2	79741961	T	A	Substitution	intron_variant	*CTNNA2*	-
Twin_B	2	84976628	T	A	Substitution	intron_variant	*KCMF1*	rs1459851336
Twin_B	2	89011497	G	A	Substitution	upstream_gene_variant	*IGKV1*	rs1327184549
Twin_B	2	92889972	CAT	C	Deletion	intergenic_variant	-	rs1381310181
Twin_B	2	94087365	T	C	Substitution	intergenic_variant	-	rs1304472866
Twin_B	2	100572501	T	C	Substitution	downstream_gene_variant	*PDCL3*	-
Twin_B	2	103642003	G	A	Substitution	intergenic_variant	-	-
Twin_B	2	104572208	AC	A	Deletion	intergenic_variant	-	rs1234997111
Twin_B	2	110152812	G	A	Substitution	intron_variant	*NPHP1*	-
Twin_B	2	112972342	G	A	Substitution	regulatory_region_variant	-	-
Twin_B	2	113360949	C	G	Substitution	intron_variant	*AC016745.1*	-
Twin_B	2	114347202	C	G	Substitution	intergenic_variant	-	-
Twin_B	2	122468457	C	T	Substitution	intron_variant	*AC011246.1*	-
Twin_B	2	124707645	G	T	Substitution	intron_variant	*CNTNAP5*	-
Twin_B	2	125662877	G	T	Substitution	upstream_gene_variant	*AC097499.2*	-
Twin_B	2	130970780	G	C	Substitution	intron_variant	*ARHGEF4*	-
Twin_B	2	136809746	AG	A	Deletion	intron_variant	*THSD7B*	-
Twin_B	2	139199861	C	T	Substitution	intergenic_variant	-	rs9646664
Twin_B	2	139215973	T	A	Substitution	intergenic_variant	-	-
Twin_B	2	139897503	C	CTT	Insertion	intergenic_variant	-	-
Twin_B	2	140219327	G	T	Substitution	downstream_gene_variant	*MTND1P27*	-
Twin_B	2	142622241	G	A	Substitution	intergenic_variant	-	-
Twin_B	2	147580489	T	C	Substitution	intergenic_variant	-	-
Twin_B	2	156625930	G	A	Substitution	intergenic_variant	-	rs919742480
Twin_B	2	156749565	A	G	Substitution	intergenic_variant	-	-
Twin_B	2	157345619	GTGGAAAAGAAGCTGCTGGGATCTACTTACC	G	Deletion	intergenic_variant	-	-
Twin_B	2	158735231	G	A	Substitution	intron_variant	*AC005042.1*	-
Twin_B	2	166646939	T	TC	Insertion	intergenic_variant	-	rs397801435
Twin_B	2	168435246	A	T	Substitution	intergenic_variant	-	rs13393085
Twin_B	2	181754253	G	A	Substitution	intergenic_variant	-	rs928244754
Twin_B	2	186809029	C	A	Substitution	intergenic_variant	-	-

Twin_B	2	187978655	C	T	Substitution	intron_variant	*LINC01090*	-
Twin_B	2	190310023	C	T	Substitution	intron_variant	*HIBCH*	rs1380735746
Twin_B	2	191398351	T	G	Substitution	intron_variant	*MYO1B*	-
Twin_B	2	197588962	G	A	Substitution	intron_variant	*RFTN2*	-
Twin_B	2	202594928	C	T	Substitution	intergenic_variant	-	rs987671953
Twin_B	2	202941148	T	C	Substitution	intron_variant	*CARF*	-
Twin_B	2	214886582	T	G	Substitution	intron_variant	*SNHG31*	-
Twin_B	2	215762883	A	C	Substitution	intron_variant	*LINC00607*	-
Twin_B	2	220248897	TTCTC	T	Deletion	intron_variant	*AC019211.1*	-
Twin_B	2	222991555	T	C	Substitution	intergenic_variant	-	-
Twin_B	2	226321782	C	T	Substitution	intergenic_variant	-	-
Twin_B	2	228241318	G	A	Substitution	intergenic_variant	-	rs746719263
Twin_B	2	236024598	A	G	Substitution	intron_variant	*AGAP1*	-
Twin_B	2	238340846	T	G	Substitution	intron_variant	*TRAF3IP1*	rs200969651
Twin_B	2	238788248	G	A	Substitution	downstream_gene_variant	*AC145625.1*	-
Twin_B	3	4096954	C	T	Substitution	intron_variant	*SUMF1*	rs993902730
Twin_B	3	6268058	G	C	Substitution	intron_variant	*AC026167.1*	-
Twin_B	3	8179865	C	T	Substitution	intron_variant	*LMCD1*	-
Twin_B	3	8330619	T	A	Substitution	intron_variant	*LMCD1*	-
Twin_B	3	8749340	G	C	Substitution	downstream_gene_variant	*CAV3*	-
Twin_B	3	8766880	C	G	Substitution	downstream_gene_variant	*OXTR*	-
Twin_B	3	8800977	A	G	Substitution	intron_variant	*RAD18*	-
Twin_B	3	9646697	T	A	Substitution	regulatory_region_variant	-	rs1001727401
Twin_B	3	39756857	A	T	Substitution	intergenic_variant	-	rs950684554
Twin_B	3	50879771	C	G	Substitution	intron_variant	*DOCK3*	-
Twin_B	3	52975648	C	A	Substitution	intron_variant	*SFMBT1*	-
Twin_B	3	54092594	G	A	Substitution	intron_variant	*AC115282.1*	-
Twin_B	3	57095586	C	T	Substitution	3_prime_UTR_variant	*IL17RD*	rs1428340204
Twin_B	3	59401686	C	T	Substitution	intron_variant	*AC126121.3*	-
Twin_B	3	62217141	G	T	Substitution	downstream_gene_variant	*PTPRG*	-
Twin_B	3	66880474	C	T	Substitution	intergenic_variant	-	rs1357891267
Twin_B	3	74463202	G	A	Substitution	intron_variant	*CNTN3*	-
Twin_B	3	80315771	G	C	Substitution	intergenic_variant	-	-
Twin_B	3	84248079	T	A	Substitution	intergenic_variant	-	rs543628716
Twin_B	3	84322937	C	A	Substitution	intergenic_variant	-	rs912367414
Twin_B	3	89748810	A	C	Substitution	intergenic_variant	-	-
Twin_B	3	95223077	C	T	Substitution	intergenic_variant	-	-
Twin_B	3	96820605	G	A	Substitution	intron_variant	*EPHA6*	-
Twin_B	3	99124717	G	C	Substitution	intergenic_variant	-	rs868051607
Twin_B	3	101873401	C	A	Substitution	regulatory_region_variant	-	-
Twin_B	3	107880323	C	T	Substitution	intron_variant	*LINC00635*	-
Twin_B	3	111393544	T	G	Substitution	intron_variant	*CD96*	-

Twin_B	3	120551312	G	T	Substitution	intergenic_variant	-	-
Twin_B	3	124318634	C	T	Substitution	intron_variant	*KALRN*	-
Twin_B	3	127184194	T	A	Substitution	intron_variant	*AC112482.2*	-
Twin_B	3	145553273	C	T	Substitution	intergenic_variant	-	rs181820859
Twin_B	3	146968300	C	A	Substitution	intron_variant	*AC092957.1*	-
Twin_B	3	148123392	C	T	Substitution	intron_variant	*AC092958.2*	rs893487280
Twin_B	3	150872501	C	T	Substitution	intron_variant	*MINDY4B*	-
Twin_B	3	152516100	G	A	Substitution	intergenic_variant	-	-
Twin_B	3	157178549	C	G	Substitution	downstream_gene_variant	*AC092944.1*	-
Twin_B	3	169022850	C	A	Substitution	intron_variant	*LINC01997*	-
Twin_B	3	179003062	C	T	Substitution	intron_variant	*ZMAT3*	-
Twin_B	3	182077482	T	A	Substitution	intergenic_variant	-	-
Twin_B	3	187366417	G	A	Substitution	upstream_gene_variant	*RTP4*	-
Twin_B	3	192580552	T	A	Substitution	intron_variant	*FGF12*	-
Twin_B	3	197965767	A	G	Substitution	intron_variant	*LMLN*	-
Twin_B	4	1811861	C	T	Substitution	3_prime_UTR_variant	*LETM1*	-
Twin_B	4	6451943	C	A	Substitution	intron_variant	*PPP2R2C*	-
Twin_B	4	12745680	C	T	Substitution	intergenic_variant	-	rs1006183911
Twin_B	4	16908350	G	T	Substitution	intergenic_variant	-	rs750655685
Twin_B	4	26875683	C	G	Substitution	intron_variant	*STIM2*	-
Twin_B	4	27652841	G	A	Substitution	intergenic_variant	-	rs1037907678
Twin_B	4	33615886	T	A	Substitution	intron_variant	*AC079772.1*	-
Twin_B	4	37418639	A	T	Substitution	intron_variant	*NWD2*	rs543621535
Twin_B	4	39429436	G	A	Substitution	intron_variant	*KLB*	-
Twin_B	4	46668719	G	C	Substitution	intergenic_variant	-	-
Twin_B	4	47220483	T	C	Substitution	intron_variant	*GABRB1*	-
Twin_B	4	49731501	T	C	Substitution	intergenic_variant	-	rs1458775311
Twin_B	4	52190355	A	G	Substitution	intergenic_variant	-	-
Twin_B	4	57634212	TG	T	Deletion	intron_variant	*AC093725.2*	-
Twin_B	4	57634214	TG	T	Deletion	intron_variant	*AC093725.2*	-
Twin_B	4	60517882	C	T	Substitution	intergenic_variant	-	-
Twin_B	4	64216296	A	G	Substitution	intergenic_variant	-	rs562121918
Twin_B	4	64665477	T	A	Substitution	intergenic_variant	-	-
Twin_B	4	67380380	G	A	Substitution	intergenic_variant	-	rs551895523
Twin_B	4	70520668	A	T	Substitution	intron_variant	*AMTN*	-
Twin_B	4	71648848	A	G	Substitution	intergenic_variant	-	rs1040824596
Twin_B	4	76047967	G	A	Substitution	intron_variant	*ART3*	-
Twin_B	4	86440642	A	G	Substitution	intron_variant	*MAPK10*	-
Twin_B	4	88387262	T	C	Substitution	upstream_gene_variant	*HERC6*	-
Twin_B	4	108394132	G	A	Substitution	intergenic_variant	-	-
Twin_B	4	115259241	A	T	Substitution	intergenic_variant	-	-
Twin_B	4	117969239	T	C	Substitution	intergenic_variant	-	rs71627746

Twin_B	4	119161162	A	C	Substitution	intron_variant	*MYOZ2*	-
Twin_B	4	120599980	G	A	Substitution	intergenic_variant	-	-
Twin_B	4	125617334	A	T	Substitution	intergenic_variant	-	-
Twin_B	4	126647636	A	G	Substitution	intron_variant	*AC097528.1*	-
Twin_B	4	127771889	A	G	Substitution	intron_variant	*SLC25A31*	rs1201508233
Twin_B	4	140154007	T	G	Substitution	5_prime_UTR_variant	*MAML3*	-
Twin_B	4	144879760	C	T	Substitution	downstream_gene_variant	*AC109811.1*	-
Twin_B	4	147616580	G	C	Substitution	intron_variant	*AC093835.1*	-
Twin_B	4	152098840	G	A	Substitution	intron_variant	*LINC02273*	-
Twin_B	4	161716379	T	C	Substitution	intron_variant	*FSTL5*	-
Twin_B	4	166613505	T	C	Substitution	intergenic_variant	-	-
Twin_B	4	168686621	G	A	Substitution	intron_variant	*PALLD*	rs1030969924
Twin_B	4	174170616	G	A	Substitution	intron_variant	*LINC02268*	rs1241611683
Twin_B	4	179639868	C	T	Substitution	intergenic_variant	-	-
Twin_B	4	180135661	C	A	Substitution	intergenic_variant	-	-
Twin_B	4	184638048	T	C	Substitution	intron_variant	*CASP3*	-
Twin_B	4	187716917	C	T	Substitution	downstream_gene_variant	*AC097521.1*	rs1306869395
Twin_B	5	4311163	C	T	Substitution	intergenic_variant	-	rs755225209
Twin_B	5	4490226	G	A	Substitution	intron_variant	*AC106799.2*	-
Twin_B	5	5721155	C	A	Substitution	intergenic_variant	-	rs1404060316
Twin_B	5	7425190	G	A	Substitution	intron_variant	*ADCY2*	-
Twin_B	5	9500811	T	C	Substitution	intron_variant	*SEMA5A*	-
Twin_B	5	11361065	G	A	Substitution	downstream_gene_variant	*CTNND2*	-
Twin_B	5	18474168	C	T	Substitution	intergenic_variant	-	-
Twin_B	5	19703201	C	T	Substitution	intron_variant	*CDH18*	-
Twin_B	5	29927998	G	A	Substitution	intergenic_variant	-	rs910971117
Twin_B	5	30228903	G	A	Substitution	intergenic_variant	-	-
Twin_B	5	32183802	G	A	Substitution	intergenic_variant	-	rs1321151704
Twin_B	5	35163531	G	A	Substitution	intron_variant	*PRLR*	-
Twin_B	5	41232152	T	G	Substitution	intron_variant	*C6*	-
Twin_B	5	41663190	G	A	Substitution	intergenic_variant	-	-
Twin_B	5	42222760	G	A	Substitution	intergenic_variant	-	-
Twin_B	5	45194560	C	T	Substitution	intergenic_variant	-	rs550304760
Twin_B	5	47101412	G	A	Substitution	intergenic_variant	-	rs1255895881
Twin_B	5	50007872	T	C	Substitution	intergenic_variant	-	-
Twin_B	5	53160317	A	G	Substitution	intergenic_variant	-	-
Twin_B	5	59291742	T	G	Substitution	intron_variant	*PDE4D*	rs57801368
Twin_B	5	63614795	GAC	G	Deletion	intergenic_variant	-	rs1396639575
Twin_B	5	72404389	A	G	Substitution	intergenic_variant	-	-
Twin_B	5	84595108	T	C	Substitution	intergenic_variant	-	rs1003756160
Twin_B	5	85129366	T	C	Substitution	intergenic_variant	-	-
Twin_B	5	93483038	G	A	Substitution	intron_variant	*NR2F1*	rs918359530

Twin_B	5	105561137	C	T	Substitution	intergenic_variant	-	-
Twin_B	5	106685060	G	A	Substitution	intergenic_variant	-	-
Twin_B	5	110479443	C	T	Substitution	intron_variant	*TMEM232*	-
Twin_B	5	119572129	C	T	Substitution	intron_variant	*HSD17B4*	-
Twin_B	5	122619069	G	A	Substitution	intergenic_variant	-	-
Twin_B	5	130265666	C	T	Substitution	intergenic_variant	-	rs62391477
Twin_B	5	134868896	C	T	Substitution	intergenic_variant	-	rs910097048
Twin_B	5	137416371	G	C	Substitution	intron_variant	*SPOCK1*	-
Twin_B	5	140836906	A	G	Substitution	3_prime_UTR_variant	*PCDHA7*	-
Twin_B	5	147046232	G	T	Substitution	intron_variant	*PPP2R2B*	-
Twin_B	5	155317926	G	T	Substitution	intergenic_variant	-	-
Twin_B	5	157005759	C	A	Substitution	intergenic_variant	-	-
Twin_B	5	158293742	G	A	Substitution	intergenic_variant	-	-
Twin_B	5	159980907	T	C	Substitution	intergenic_variant	-	-
Twin_B	5	161395724	G	A	Substitution	intron_variant	*GABRB2*	-
Twin_B	5	161830310	G	T	Substitution	intergenic_variant	-	-
Twin_B	5	166256348	G	A	Substitution	intergenic_variant	-	-
Twin_B	5	175182485	G	A	Substitution	intergenic_variant	-	-
Twin_B	5	178484296	T	G	Substitution	intron_variant	*COL23A1*	-
Twin_B	6	1850433	CT	C	Deletion	intron_variant	*GMDS*	-
Twin_B	6	18553216	G	T	Substitution	intron_variant	*MIR548A1HG*	-
Twin_B	6	20182475	T	G	Substitution	intron_variant	*MBOAT1*	-
Twin_B	6	23725753	C	T	Substitution	intergenic_variant	-	-
Twin_B	6	23820823	AG	A	Deletion	intergenic_variant	-	-
Twin_B	6	32530993	T	C	Substitution	regulatory_region_variant	-	rs74714255
Twin_B	6	33355096	C	T	Substitution	intergenic_variant	-	-
Twin_B	6	40322738	T	C	Substitution	intergenic_variant	-	-
Twin_B	6	45795560	T	G	Substitution	intergenic_variant	-	-
Twin_B	6	49245413	G	A	Substitution	intergenic_variant	-	-
Twin_B	6	49866189	C	T	Substitution	intron_variant	*CRISP1*	-
Twin_B	6	50314470	C	T	Substitution	intergenic_variant	-	rs192304872
Twin_B	6	68164502	G	A	Substitution	intergenic_variant	-	rs539171573
Twin_B	6	73054903	C	A	Substitution	intron_variant	*KCNQ5*	-
Twin_B	6	77608472	C	G	Substitution	intergenic_variant	-	rs145374562
Twin_B	6	78332113	A	G	Substitution	intergenic_variant	-	-
Twin_B	6	80558206	T	C	Substitution	upstream_gene_variant	*AL590824.1*	-
Twin_B	6	95406340	C	T	Substitution	intergenic_variant	-	rs548629996
Twin_B	6	101437584	G	A	Substitution	intron_variant	*GRIK2*	rs1368442237
Twin_B	6	102787939	T	A	Substitution	intergenic_variant	-	-
Twin_B	6	105574693	A	C	Substitution	intron_variant	*AL096794.1*	-
Twin_B	6	110560428	G	A	Substitution	downstream_gene_variant	*AL512430.3*	-
Twin_B	6	115939145	A	G	Substitution	3_prime_UTR_variant	*FRK*	-

Twin_B	6	132482937	C	T	Substitution	intron_variant	*STX7*	-
Twin_B	6	133738256	C	A	Substitution	intron_variant	*TARID*	-
Twin_B	6	153868755	C	A	Substitution	intergenic_variant	-	-
Twin_B	6	155942473	C	T	Substitution	intron_variant	*AL589693.1*	rs1029874863
Twin_B	6	157796066	T	G	Substitution	intron_variant	*SNX9*	-
Twin_B	6	160228462	G	T	Substitution	intron_variant	*SLC22A2*	-
Twin_B	6	160512492	C	T	Substitution	intron_variant	*LPAL2*	rs573849624
Twin_B	6	163903570	G	A	Substitution	intergenic_variant	-	rs138646256
Twin_B	6	164337205	G	A	Substitution	intergenic_variant	-	rs1395517073
Twin_B	6	170147589	C	A	Substitution	downstream_gene_variant	*AL596442.4*	-
Twin_B	7	4358841	C	T	Substitution	intergenic_variant	-	rs746784235
Twin_B	7	7383076	A	T	Substitution	intron_variant	*COL28A1*	-
Twin_B	7	9012014	G	A	Substitution	intergenic_variant	-	-
Twin_B	7	10181486	C	T	Substitution	intergenic_variant	-	-
Twin_B	7	20905885	C	G	Substitution	intron_variant	*LINC01162*	-
Twin_B	7	26002719	G	A	Substitution	intergenic_variant	-	-
Twin_B	7	27125974	G	A	Substitution	downstream_gene_variant	*AC004080.6*	-
Twin_B	7	30124532	G	T	Substitution	downstream_gene_variant	*AC007036.5*	-
Twin_B	7	33443171	A	T	Substitution	intron_variant	*BBS9*	-
Twin_B	7	33920177	G	A	Substitution	intron_variant	*BMPER*	-
Twin_B	7	34052098	C	T	Substitution	intron_variant	*BMPER*	-
Twin_B	7	43433707	A	C	Substitution	intron_variant	*HECW1*	-
Twin_B	7	44053423	G	A	Substitution	downstream_gene_variant	*DBNL*	rs1254859423
Twin_B	7	45053757	A	G	Substitution	intron_variant	*CCM2*	-
Twin_B	7	45393430	C	G	Substitution	upstream_gene_variant	*ELK1P1*	-
Twin_B	7	48916465	T	C	Substitution	upstream_gene_variant	*CDC14C*	rs1245635730
Twin_B	7	54587046	C	A	Substitution	intergenic_variant	-	-
Twin_B	7	57036330	G	A	Substitution	intergenic_variant	-	rs368641980
Twin_B	7	60485855	A	C	Substitution	intergenic_variant	-	rs1335884497
Twin_B	7	60938781	CA	C	Deletion	intergenic_variant	-	rs1250869720
Twin_B	7	61436516	T	A	Substitution	intergenic_variant	-	rs1169588355
Twin_B	7	61970818	T	C	Substitution	intergenic_variant	-	rs1283251044
Twin_B	7	62882059	A	C	Substitution	intergenic_variant	-	rs112905112
Twin_B	7	62882071	C	A	Substitution	intergenic_variant	-	rs1395947656
Twin_B	7	65034946	G	A	Substitution	upstream_gene_variant	*AC073210.2*	rs954732530
Twin_B	7	65590326	T	C	Substitution	intergenic_variant	-	-
Twin_B	7	67521209	C	T	Substitution	intergenic_variant	-	rs183903994
Twin_B	7	71219697	C	T	Substitution	intron_variant	*GALNT17*	-
Twin_B	7	71503989	G	A	Substitution	intron_variant	*GALNT17*	-
Twin_B	7	73980208	CAA	C	Deletion	intergenic_variant	-	rs60681274
Twin_B	7	75784737	G	A	Substitution	intron_variant	*CCL26*	-
Twin_B	7	86130259	T	G	Substitution	intergenic_variant	-	-

Twin_B	7	102788991	TATG	T	Deletion	downstream_gene_variant	*FAM185A*	rs1163997473
Twin_B	7	108865841	G	A	Substitution	intergenic_variant	-	-
Twin_B	7	111235913	G	A	Substitution	intron_variant	*IMMP2L*	-
Twin_B	7	116155279	G	C	Substitution	intron_variant	*TFEC*	-
Twin_B	7	134178896	G	A	Substitution	intron_variant	*LRGUK*	-
Twin_B	7	137739877	G	T	Substitution	intron_variant	*DGKI*	rs190071670
Twin_B	7	142637901	C	A	Substitution	downstream_gene_variant	*TRBV21*	-
Twin_B	7	146668441	T	TGG	Insertion	intron_variant	*CNTNAP2*	rs35693921
Twin_B	7	150085019	G	A	Substitution	intergenic_variant	-	rs1461424202
Twin_B	7	152410761	T	C	Substitution	intron_variant	*KMT2C*	rs80138121
Twin_B	7	156931430	C	T	Substitution	intergenic_variant	-	-
Twin_B	8	924933	C	T	Substitution	intron_variant	*DLGAP2*	-
Twin_B	8	3391860	G	T	Substitution	intron_variant	*CSMD1*	-
Twin_B	8	4997791	G	A	Substitution	regulatory_region_variant	-	-
Twin_B	8	5826762	C	T	Substitution	intergenic_variant	-	-
Twin_B	8	19798556	G	T	Substitution	intergenic_variant	-	-
Twin_B	8	20863668	T	A	Substitution	intergenic_variant	-	-
Twin_B	8	25151260	C	A	Substitution	intergenic_variant	-	-
Twin_B	8	26940513	G	A	Substitution	intergenic_variant	-	-
Twin_B	8	32400294	G	T	Substitution	intron_variant	*NRG1*	-
Twin_B	8	32524703	G	A	Substitution	intron_variant	*NRG1*	-
Twin_B	8	34194743	G	C	Substitution	intron_variant	*AC087855.2*	-
Twin_B	8	41188472	A	T	Substitution	intergenic_variant	-	-
Twin_B	8	43877252	G	GT	Insertion	intergenic_variant	-	-
Twin_B	8	44701946	T	G	Substitution	intergenic_variant	-	rs1325351437
Twin_B	8	46315152	G	A	Substitution	intergenic_variant	-	-
Twin_B	8	49102414	A	C	Substitution	intron_variant	*AC044893.1*	-
Twin_B	8	50264084	A	G	Substitution	intron_variant	*SNTG1*	-
Twin_B	8	51178687	G	A	Substitution	intergenic_variant	-	rs1207366775
Twin_B	8	53427578	TAC	T	Deletion	intron_variant	*AC022034.3*	-
Twin_B	8	57557945	G	A	Substitution	intron_variant	*AC090796.1*	-
Twin_B	8	58506466	G	T	Substitution	downstream_gene_variant	*PPIAP85*	rs973571307
Twin_B	8	59792693	T	A	Substitution	intergenic_variant	-	-
Twin_B	8	64469993	C	A	Substitution	intergenic_variant	-	-
Twin_B	8	69896186	C	A	Substitution	intergenic_variant	-	rs1204844083
Twin_B	8	70358021	AGCCTG	A	Deletion	downstream_gene_variant	*RNY3P14*	-
Twin_B	8	71477385	T	C	Substitution	intron_variant	*EYA1*	-
Twin_B	8	78483436	T	C	Substitution	intron_variant	*PKIA*	-
Twin_B	8	81785469	A	G	Substitution	intergenic_variant	-	rs1439987215
Twin_B	8	82359769	T	C	Substitution	intron_variant	*AC060765.2*	-
Twin_B	8	84130068	C	T	Substitution	intron_variant	*AC015522.1*	rs114417960
Twin_B	8	87159687	A	T	Substitution	intron_variant	*CNBD1*	-

Twin_B	8	87159688	G	A	Substitution	intron_variant	*CNBD1*	-
Twin_B	8	98496864	T	C	Substitution	intron_variant	*STK3*	rs1015493993
Twin_B	8	98657310	C	T	Substitution	intron_variant	*STK3*	-
Twin_B	8	100119116	G	A	Substitution	intron_variant	*RGS22*	-
Twin_B	8	109438926	A	G	Substitution	missense_variant	*PKHD1L1*	-
Twin_B	8	109534244	C	T	Substitution	3_prime_UTR_variant	*PKHD1L1*	rs539684370
Twin_B	8	111647834	G	C	Substitution	intron_variant	*LINC02237*	-
Twin_B	8	111905799	G	A	Substitution	intergenic_variant	-	rs1453724199
Twin_B	8	113516282	C	A	Substitution	intergenic_variant	-	-
Twin_B	8	114563854	C	T	Substitution	intergenic_variant	-	-
Twin_B	8	116551195	C	T	Substitution	intergenic_variant	-	-
Twin_B	8	122234933	G	A	Substitution	intergenic_variant	-	rs1423584842
Twin_B	8	126381321	C	T	Substitution	intergenic_variant	-	rs1433139292
Twin_B	8	131552936	T	A	Substitution	intergenic_variant	-	-
Twin_B	8	133562727	G	A	Substitution	downstream_gene_variant	*ST3GAL1*	-
Twin_B	8	136497219	A	G	Substitution	intron_variant	*LINC02055*	-
Twin_B	8	139066773	G	A	Substitution	intergenic_variant	-	-
Twin_B	9	595757	G	A	Substitution	intron_variant	*KANK1*	rs1028227944
Twin_B	9	5583708	G	A	Substitution	intron_variant	*AL162253.2*	-
Twin_B	9	9208774	T	C	Substitution	intron_variant	*PTPRD*	-
Twin_B	9	10229559	G	A	Substitution	intron_variant	*PTPRD*	-
Twin_B	9	12626664	C	T	Substitution	intergenic_variant	-	-
Twin_B	9	12964481	A	T	Substitution	intron_variant	*AL161449.2*	rs1563767337
Twin_B	9	21096138	A	G	Substitution	intergenic_variant	-	rs896126975
Twin_B	9	23036773	C	T	Substitution	intron_variant	*AL391117.1*	-
Twin_B	9	24723875	G	T	Substitution	intergenic_variant	-	-
Twin_B	9	24924904	G	A	Substitution	intergenic_variant	-	rs193241464
Twin_B	9	38292964	C	T	Substitution	intergenic_variant	-	-
Twin_B	9	38995507	T	C	Substitution	intron_variant	*AL953883.1*	rs1422022710
Twin_B	9	42290039	T	A	Substitution	intron_variant	*AL445584.2*	rs1211321020
Twin_B	9	42760705	C	T	Substitution	intron_variant	*BX664718.2*	rs56025544
Twin_B	9	42945900	G	A	Substitution	intron_variant	*ANKRD20A7P*	-
Twin_B	9	65672213	T	C	Substitution	intron_variant	*CBWD5*	rs796503817
Twin_B	9	66779265	C	T	Substitution	intergenic_variant	-	rs1271105675
Twin_B	9	67205531	TG	T	Deletion	intron_variant	*AL162233.1*	rs368975415
Twin_B	9	73452437	GTTTC	G	Deletion	intergenic_variant	-	-
Twin_B	9	74317541	T	C	Substitution	intergenic_variant	-	-
Twin_B	9	77105186	C	T	Substitution	intergenic_variant	-	rs538603299
Twin_B	9	87267784	G	A	Substitution	intergenic_variant	-	rs1328259639
Twin_B	9	88848472	C	A	Substitution	intergenic_variant	-	-
Twin_B	9	95722226	G	T	Substitution	intergenic_variant	-	-
Twin_B	9	98676266	A	T	Substitution	intron_variant	*GABBR2*	-

Twin_B	9	102339974	A	G	Substitution	intergenic_variant	-	-
Twin_B	9	102993672	C	A	Substitution	upstream_gene_variant	*CYLC2*	-
Twin_B	9	103368304	G	A	Substitution	intron_variant	*AL390962.1*	-
Twin_B	9	104257777	G	T	Substitution	intergenic_variant	-	-
Twin_B	9	104331598	T	G	Substitution	upstream_gene_variant	*AL512646.1*	-
Twin_B	9	105298502	A	G	Substitution	intron_variant	*SLC44A1*	-
Twin_B	9	118261593	G	A	Substitution	intergenic_variant	-	rs1353495123
Twin_B	9	124851965	C	T	Substitution	upstream_gene_variant	*WDR38*	rs962035631
Twin_B	9	127439569	C	T	Substitution	intron_variant	*ZNF79*	-
Twin_B	9	130429019	C	T	Substitution	intron_variant	*HMCN2*	rs554518691
Twin_B	9	131860557	T	A	Substitution	missense_variant	*MED27*	-
Twin_B	9	134007274	C	T	Substitution	intergenic_variant	-	-
Twin_B	9	135090386	T	G	Substitution	intron_variant	*OLFM1*	rs56337838
Twin_B	10	1176537	C	T	Substitution	downstream_gene_variant	*ADARB2*	rs757910551
Twin_B	10	9492286	A	C	Substitution	intergenic_variant	-	rs148903298
Twin_B	10	10933457	T	A	Substitution	downstream_gene_variant	*LINC00710*	-
Twin_B	10	12067169	A	G	Substitution	upstream_gene_variant	*DHTKD1*	rs1055077923
Twin_B	10	14501740	C	A	Substitution	intergenic_variant	-	-
Twin_B	10	19776842	A	T	Substitution	intron_variant	*MALRD1*	-
Twin_B	10	24984824	G	A	Substitution	missense_variant	*ENKUR*	rs200773323
Twin_B	10	30158617	C	A	Substitution	intergenic_variant	-	-
Twin_B	10	33849532	T	C	Substitution	intergenic_variant	-	-
Twin_B	10	37826153	C	T	Substitution	downstream_gene_variant	*ZNF248*	-
Twin_B	10	42358812	A	G	Substitution	intron_variant	*BX322639.1*	-
Twin_B	10	46311814	C	A	Substitution	downstream_gene_variant	*ANTXRL*	-
Twin_B	10	54568022	G	T	Substitution	intron_variant	*PCDH15*	-
Twin_B	10	65525504	T	C	Substitution	intergenic_variant	-	-
Twin_B	10	73104873	G	A	Substitution	upstream_gene_variant	*AL731563.3*	-
Twin_B	10	75129152	A	T	Substitution	downstream_gene_variant	*RPS26P42*	rs1477868012
Twin_B	10	82739562	G	A	Substitution	intron_variant	*NRG3*	-
Twin_B	10	86750662	C	A	Substitution	non_coding_transcript_ exon_variant	*AC067750.1*	-
Twin_B	10	88654107	TA	T	Deletion	intergenic_variant	-	rs200426640
Twin_B	10	90700494	C	T	Substitution	intergenic_variant	-	-
Twin_B	10	92770872	T	A	Substitution	intergenic_variant	-	-
Twin_B	10	93234495	G	T	Substitution	intergenic_variant	-	rs184258432
Twin_B	10	102661031	C	T	Substitution	downstream_gene_variant	*TRIM8*	-
Twin_B	10	103273122	G	A	Substitution	TF_binding_site_variant	*INA*	rs1286474689
Twin_B	10	110609195	T	A	Substitution	downstream_gene_variant	*SMC3*	-
Twin_B	10	115355253	A	G	Substitution	intron_variant	*ATRNL1*	-
Twin_B	10	116513252	G	A	Substitution	intergenic_variant	-	-
Twin_B	10	118845653	C	T	Substitution	intron_variant	*AL157388.1*	-
Twin_B	10	121236516	G	A	Substitution	intergenic_variant	-	rs75201857

Twin_B	10	125904313	C	T	Substitution	intron_variant	*FANK1*	rs571397776
Twin_B	10	131096200	G	A	Substitution	downstream_gene_variant	*TCERG1L*	-
Twin_B	10	132055519	G	A	Substitution	intron_variant	*JAKMIP3*	rs11813033
Twin_B	11	2172588	G	A	Substitution	upstream_gene_variant	*MIR4686*	rs970284747
Twin_B	11	4222159	G	A	Substitution	intergenic_variant	-	rs78524524
Twin_B	11	4861410	G	A	Substitution	intron_variant	*MMP26*	-
Twin_B	11	5952018	A	G	Substitution	downstream_gene_variant	*OR56A3*	-
Twin_B	11	8836985	G	A	Substitution	intron_variant	*DENND2B*	-
Twin_B	11	11456484	C	T	Substitution	intron_variant	*GALNT18*	-
Twin_B	11	19063121	G	A	Substitution	upstream_gene_variant	*MRGPRX2*	-
Twin_B	11	20315541	A	G	Substitution	intergenic_variant	-	-
Twin_B	11	20325176	G	T	Substitution	intergenic_variant	-	-
Twin_B	11	36738318	A	G	Substitution	intergenic_variant	-	rs1297968115
Twin_B	11	36973840	G	A	Substitution	intergenic_variant	-	rs983687999
Twin_B	11	42300900	G	A	Substitution	intergenic_variant	-	rs915445215
Twin_B	11	42391773	A	G	Substitution	intergenic_variant	-	-
Twin_B	11	44879657	AG	A	Deletion	intron_variant	*TSPAN18*	-
Twin_B	11	54940328	C	A	Substitution	intergenic_variant	-	-
Twin_B	11	55783372	G	A	Substitution	upstream_gene_variant	*OR5D15P*	rs566667825
Twin_B	11	81625515	T	C	Substitution	intergenic_variant	-	rs1018593916
Twin_B	11	82290505	C	T	Substitution	intron_variant	*MIR4300HG*	rs181088482
Twin_B	11	82375060	A	T	Substitution	intron_variant	*MIR4300HG*	-
Twin_B	11	83760647	C	A	Substitution	intron_variant	*DLG2*	-
Twin_B	11	88442713	G	T	Substitution	intergenic_variant	-	-
Twin_B	11	91384045	G	T	Substitution	downstream_gene_variant	*AP002791.1*	-
Twin_B	11	93116767	C	A	Substitution	intergenic_variant	-	-
Twin_B	11	93907782	C	T	Substitution	intergenic_variant	-	rs1382214357
Twin_B	11	103587114	C	G	Substitution	intergenic_variant	-	rs1026010882
Twin_B	11	107071946	A	G	Substitution	intergenic_variant	-	-
Twin_B	11	112616847	C	T	Substitution	intron_variant	*LINC02763*	rs920020081
Twin_B	11	114708783	G	A	Substitution	downstream_gene_variant	*NXPE2*	-
Twin_B	11	114958122	G	T	Substitution	intergenic_variant	-	-
Twin_B	11	117410900	C	A	Substitution	downstream_gene_variant	*CEP164*	-
Twin_B	11	122025753	C	T	Substitution	downstream_gene_variant	*MIR100HG*	-
Twin_B	11	122979808	C	A	Substitution	intron_variant	*BSX*	-
Twin_B	11	124291010	G	A	Substitution	intergenic_variant	-	-
Twin_B	11	128554716	C	T	Substitution	intron_variant	*ETS1*	rs78814353
Twin_B	11	132785635	G	A	Substitution	intron_variant	*OPCML*	-
Twin_B	11	133338345	C	T	Substitution	intron_variant	*OPCML*	rs907145809
Twin_B	11	133761282	T	A	Substitution	intergenic_variant	-	-
Twin_B	12	6117683	C	T	Substitution	downstream_gene_variant	*VWF*	rs546633664
Twin_B	12	6307820	C	T	Substitution	regulatory_region_variant	-	rs1256264888

Twin_B	12	6520513	G	A	Substitution	downstream_gene_variant	*NCAPD2*	-
Twin_B	12	6789295	C	T	Substitution	intron_variant	*CD4*	-
Twin_B	12	8205043	GC	G	Deletion	downstream_gene_variant	*FAM66C*	rs144267844
Twin_B	12	9683004	G	A	Substitution	downstream_gene_variant	*CLEC2D*	rs188982524
Twin_B	12	11785599	A	G	Substitution	intron_variant	*ETV6*	rs778627490
Twin_B	12	18037838	C	T	Substitution	intergenic_variant	-	-
Twin_B	12	20532321	G	A	Substitution	intron_variant	*PDE3A*	-
Twin_B	12	24620263	G	A	Substitution	intergenic_variant	-	-
Twin_B	12	25350278	C	T	Substitution	intergenic_variant	-	rs944670622
Twin_B	12	26030102	G	A	Substitution	intron_variant	*RASSF8*	rs771217533
Twin_B	12	32986809	G	A	Substitution	non_coding_transcript_ exon_variant	*AC087311.1*	-
Twin_B	12	34148556	C	T	Substitution	upstream_gene_variant	*AC140847.1*	rs1252261441
Twin_B	12	35988466	T	C	Substitution	intergenic_variant	-	rs1164988538
Twin_B	12	38746830	T	C	Substitution	intron_variant	*CPNE8*	-
Twin_B	12	43641305	G	A	Substitution	intergenic_variant	-	rs568390024
Twin_B	12	46321777	C	T	Substitution	intergenic_variant	-	rs150303645
Twin_B	12	50558428	A	T	Substitution	intron_variant	*DIP2B*	-
Twin_B	12	61470230	G	C	Substitution	intergenic_variant	-	rs1421114192
Twin_B	12	68411586	C	A	Substitution	intron_variant	*LINC02384*	-
Twin_B	12	77504158	G	A	Substitution	intron_variant	*NAV3*	rs991324887
Twin_B	12	78382902	C	T	Substitution	intron_variant	*AC128707.1*	-
Twin_B	12	79065377	C	T	Substitution	intron_variant	*SYT1*	rs993033305
Twin_B	12	80780558	A	T	Substitution	intergenic_variant	-	-
Twin_B	12	83300749	C	T	Substitution	intergenic_variant	-	rs538003003
Twin_B	12	84484910	G	A	Substitution	intergenic_variant	-	-
Twin_B	12	86386833	C	A	Substitution	intron_variant	*MGAT4C*	-
Twin_B	12	87232193	G	A	Substitution	intergenic_variant	-	-
Twin_B	12	87810848	T	G	Substitution	intergenic_variant	-	-
Twin_B	12	88071791	G	A	Substitution	missense_variant	*CEP290*	rs761572735
Twin_B	12	90780665	C	T	Substitution	intron_variant	*AC112481.2*	-
Twin_B	12	95719552	T	C	Substitution	intron_variant	*NTN4*	COSV59220221
Twin_B	12	96233025	A	G	Substitution	intron_variant	*ELK3*	-
Twin_B	12	100396583	T	A	Substitution	intron_variant	*SLC17A8*	rs1220972791
Twin_B	12	105124962	C	T	Substitution	intron_variant	*WASHC4*	-
Twin_B	12	109600533	C	G	Substitution	downstream_gene_variant	*MVK*	-
Twin_B	12	113992218	G	A	Substitution	intergenic_variant	-	-
Twin_B	12	114026760	G	A	Substitution	intergenic_variant	-	rs142779521
Twin_B	12	115283072	C	A	Substitution	intergenic_variant	-	-
Twin_B	12	116627058	G	C	Substitution	intergenic_variant	-	-
Twin_B	12	121968151	A	T	Substitution	missense_variant	*CFAP251*	-
Twin_B	12	126210035	C	T	Substitution	intron_variant	*AC007368.1*	-
Twin_B	12	128451756	C	T	Substitution	intron_variant	*TMEM132C*	-

Twin_B	12	129158414	A	G	Substitution	intron_variant	*TMEM132D*	-
Twin_B	12	129545572	G	A	Substitution	intron_variant	*TMEM132D*	rs548980444
Twin_B	12	132821132	C	T	Substitution	downstream_gene_variant	*CHFR*	rs981825385
Twin_B	13	16017183	C	T	Substitution	intergenic_variant	-	rs1346805079
Twin_B	13	16661736	C	A	Substitution	intergenic_variant	-	rs1306983302
Twin_B	13	17304667	T	A	Substitution	intergenic_variant	-	rs1456450575
Twin_B	13	17466593	C	T	Substitution	intergenic_variant	-	rs1444264382
Twin_B	13	34712768	G	A	Substitution	intergenic_variant	-	rs956638263
Twin_B	13	37492154	T	A	Substitution	intergenic_variant	-	rs5028161
Twin_B	13	51338069	C	T	Substitution	intron_variant	*SERPINE3*	rs943522065
Twin_B	13	57247644	C	T	Substitution	intergenic_variant	-	rs1015436925
Twin_B	13	64005202	A	C	Substitution	intron_variant	*LINC00355*	-
Twin_B	13	66744717	G	A	Substitution	intron_variant	*PCDH9*	rs1436271609
Twin_B	13	68771491	A	G	Substitution	intergenic_variant	-	-
Twin_B	13	81289794	T	C	Substitution	intron_variant	*AL353633.1*	rs1481888041
Twin_B	13	92237367	G	A	Substitution	downstream_gene_variant	*AL157815.1*	rs1265338638
Twin_B	13	95505939	C	G	Substitution	intron_variant	*CLDN10*	-
Twin_B	13	106932614	C	T	Substitution	intergenic_variant	-	-
Twin_B	13	109688927	C	T	Substitution	intron_variant	*AL163541.1*	-
Twin_B	13	112351281	G	A	Substitution	intergenic_variant	-	rs115643630
Twin_B	13	112451541	C	T	Substitution	intergenic_variant	-	rs1471601068
Twin_B	14	25383375	A	AT	Insertion	intergenic_variant	-	rs1443265250
Twin_B	14	28045753	A	G	Substitution	intergenic_variant	-	rs1373127559
Twin_B	14	28160448	C	G	Substitution	intergenic_variant	-	-
Twin_B	14	28481339	C	T	Substitution	intergenic_variant	-	rs987212689
Twin_B	14	28933071	A	T	Substitution	downstream_gene_variant	*LINC02327*	-
Twin_B	14	34755189	T	C	Substitution	downstream_gene_variant	*BAZ1A*	-
Twin_B	14	39719161	C	T	Substitution	intron_variant	*AL049828.1*	-
Twin_B	14	39760915	GC	G	Deletion	intron_variant	*AL049828.1*	-
Twin_B	14	40646136	G	C	Substitution	intron_variant	*AL390800.1*	-
Twin_B	14	42163357	C	T	Substitution	intergenic_variant	-	-
Twin_B	14	44806590	C	A	Substitution	intergenic_variant	-	-
Twin_B	14	45687961	C	A	Substitution	intergenic_variant	-	-
Twin_B	14	49208635	C	T	Substitution	intergenic_variant	-	-
Twin_B	14	52546562	G	A	Substitution	intron_variant	*TXNDC16*	-
Twin_B	14	55273174	G	GTT	Insertion	intron_variant	*FBXO34*	-
Twin_B	14	62950522	C	T	Substitution	missense_variant	*KCNH5*	rs587777164, CM1310653, COSV59758318, COSV59758899
Twin_B	14	76286201	T	G	Substitution	downstream_gene_variant	*AC016526.3*	-
Twin_B	14	87098315	C	T	Substitution	intergenic_variant	-	-
Twin_B	14	94965692	G	C	Substitution	upstream_gene_variant	*LINC02279*	-
Twin_B	14	96647102	C	T	Substitution	intergenic_variant	-	-

Twin_B	14	98254213	A	T	Substitution	intergenic_variant	-	rs200791071
Twin_B	14	99920248	A	G	Substitution	downstream_gene_variant	*EML1*	-
Twin_B	15	17031425	C	T	Substitution	intergenic_variant	-	rs988560630
Twin_B	15	24913380	C	T	Substitution	downstream_gene_variant	*AC090983.2*	rs995136908
Twin_B	15	25046210	A	G	Substitution	downstream_gene_variant	*SNHG14*	-
Twin_B	15	26017195	A	G	Substitution	intron_variant	*LINC02346*	-
Twin_B	15	40140978	A	G	Substitution	intergenic_variant	-	-
Twin_B	15	45848202	CAAA	C	Deletion	intron_variant	*AC068722.1*	rs1232293898
Twin_B	15	48080087	A	G	Substitution	intergenic_variant	-	-
Twin_B	15	48928081	AG	A	Deletion	intron_variant	*SHC4*	-
Twin_B	15	60120205	G	A	Substitution	regulatory_region_variant	-	-
Twin_B	15	60352764	T	C	Substitution	downstream_gene_variant	*ANXA2*	rs1242885715
Twin_B	15	71202412	A	G	Substitution	intron_variant	*THSD4*	-
Twin_B	15	72502854	C	T	Substitution	intron_variant	*ARIH1*	rs1435288541
Twin_B	15	77267388	A	G	Substitution	intron_variant	*PEAK1*	-
Twin_B	15	79562906	G	A	Substitution	upstream_gene_variant	*AC023968.2*	-
Twin_B	15	80681168	T	A	Substitution	intron_variant	*ABHD17C*	-
Twin_B	15	83132180	G	C	Substitution	3_prime_UTR_variant	*HDGFL3*	-
Twin_B	15	87983261	T	A	Substitution	intron_variant	*NTRK3*	-
Twin_B	15	95004101	T	C	Substitution	intron_variant	*AC087633.2*	-
Twin_B	16	737082	C	G	Substitution	downstream_gene_variant	*CIAO3*	-
Twin_B	16	1550273	G	A	Substitution	downstream_gene_variant	*AL031719.2*	-
Twin_B	16	5534803	T	C	Substitution	intron_variant	*RBFOX1*	-
Twin_B	16	9532674	G	T	Substitution	intergenic_variant	-	rs1341344135
Twin_B	16	18537225	T	TAG	Insertion	downstream_gene_variant	*AC136618.2*	-
Twin_B	16	19362955	T	G	Substitution	intron_variant	*AC130456.2*	rs140765053
Twin_B	16	20424714	G	A	Substitution	TF_binding_site_variant	*ACSM5*	-
Twin_B	16	22677228	G	A	Substitution	intergenic_variant	-	-
Twin_B	16	32008073	G	A	Substitution	downstream_gene_variant	*AC142381.3*	-
Twin_B	16	35787574	T	C	Substitution	upstream_gene_variant	*AC106785.2*	rs1243395959
Twin_B	16	36133454	G	A	Substitution	intergenic_variant	-	-
Twin_B	16	50321074	G	A	Substitution	downstream_gene_variant	*ADCY7*	rs372755442
Twin_B	16	51336582	G	A	Substitution	intron_variant	*AC087564.1*	rs1461056097
Twin_B	16	54256124	G	C	Substitution	intron_variant	*LINC02169*	-
Twin_B	16	57821035	G	A	Substitution	downstream_gene_variant	*AC092118.1*	-
Twin_B	16	64699930	G	A	Substitution	intergenic_variant	-	-
Twin_B	16	65902588	C	T	Substitution	intergenic_variant	-	rs115844235
Twin_B	16	67105628	T	A	Substitution	downstream_gene_variant	*CBFB*	rs1349050758
Twin_B	16	67608941	A	G	Substitution	intron_variant	*CTCF*	rs892105462
Twin_B	16	68548732	C	G	Substitution	intron_variant	*ZFP90*	rs564497116
Twin_B	16	69148663	G	A	Substitution	intron_variant	*UTP4*	rs1469117705
Twin_B	16	70802883	G	T	Substitution	3_prime_UTR_variant	*HYDIN*	-

Twin_B	16	73552032	A	T	Substitution	intron_variant	*ZFHX3*	rs562962066
Twin_B	16	77256710	G	T	Substitution	intron_variant	*ADAMTS18*	-
Twin_B	16	77493285	T	C	Substitution	intergenic_variant	-	-
Twin_B	16	82066056	G	T	Substitution	intron_variant	*HSD17B2*	-
Twin_B	16	82874338	C	T	Substitution	intron_variant	*CDH13*	-
Twin_B	16	85313584	C	A	Substitution	downstream_gene_variant	*AC092275.1*	rs1483404740
Twin_B	17	4511744	T	G	Substitution	intron_variant	*SPNS2*	-
Twin_B	17	4976602	G	T	Substitution	downstream_gene_variant	*AC004771.1*	-
Twin_B	17	9021059	G	T	Substitution	regulatory_region_variant	-	-
Twin_B	17	10363095	A	T	Substitution	intron_variant	*MYH13*	-
Twin_B	17	10978272	C	T	Substitution	intergenic_variant	-	-
Twin_B	17	14670774	C	A	Substitution	intergenic_variant	-	-
Twin_B	17	18385438	G	A	Substitution	downstream_gene_variant	*AL353997.5*	rs1682277
Twin_B	17	27983490	G	A	Substitution	intron_variant	*LINC01992*	rs778201958
Twin_B	17	30755275	A	C	Substitution	intron_variant	*SUZ12P1*	-
Twin_B	17	31863521	T	A	Substitution	splice_region_variant	*UTP6*	rs78181996
Twin_B	17	34811921	G	A	Substitution	intergenic_variant	-	rs1011328968
Twin_B	17	40381174	C	T	Substitution	non_coding_transcript_ exon_variant	*AC080112.2*	-
Twin_B	17	41094327	C	T	Substitution	downstream_gene_variant	*AC100808.1*	-
Twin_B	17	41488518	C	T	Substitution	intron_variant	*KRT36*	-
Twin_B	17	41662731	C	G	Substitution	TF_binding_site_variant	*AC130686.1*	-
Twin_B	17	52148573	C	T	Substitution	intron_variant	*CA10*	-
Twin_B	17	52202053	T	G	Substitution	intergenic_variant	-	-
Twin_B	17	56525449	C	T	Substitution	intergenic_variant	-	-
Twin_B	17	59529075	G	A	Substitution	upstream_gene_variant	*LINC01476*	-
Twin_B	17	63728030	A	G	Substitution	intron_variant	*STRADA*	-
Twin_B	17	64448232	C	A	Substitution	upstream_gene_variant	*MILR1*	-
Twin_B	17	74236263	A	G	Substitution	intron_variant	*TTYH2*	rs1373649197
Twin_B	17	74482732	T	C	Substitution	intron_variant	*CD300A*	rs9900660
Twin_B	17	79561251	G	A	Substitution	intergenic_variant	-	rs924318212
Twin_B	18	524695	A	T	Substitution	intergenic_variant	-	-
Twin_B	18	924915	C	G	Substitution	downstream_gene_variant	*LINC01904*	-
Twin_B	18	8101586	A	C	Substitution	intron_variant	*PTPRM*	-
Twin_B	18	14702966	G	A	Substitution	intron_variant	*MODIFIER*	rs1201652554
Twin_B	18	14791710	G	A	Substitution	intron_variant	*ANKRD30B*	-
Twin_B	18	15293364	T	C	Substitution	intergenic_variant	-	-
Twin_B	18	20715870	G	A	Substitution	intergenic_variant	-	rs1457573477
Twin_B	18	27402273	G	A	Substitution	downstream_gene_variant	*AC068408.1*	rs1054519887
Twin_B	18	29688544	T	C	Substitution	intergenic_variant	-	rs147589821
Twin_B	18	29768107	G	A	Substitution	intergenic_variant	-	rs1280023452
Twin_B	18	30770852	G	A	Substitution	intergenic_variant	-	rs554280292
Twin_B	18	32293060	C	T	Substitution	downstream_gene_variant	*AC015563.2*	-

Twin_B	18	33117524	G	A	Substitution	intron_variant	*CCDC178*	rs138071520
Twin_B	18	33505461	T	C	Substitution	intergenic_variant	-	-
Twin_B	18	43720938	CA	C	Deletion	intergenic_variant	-	-
Twin_B	18	52406485	T	C	Substitution	intron_variant	*DCC*	-
Twin_B	18	56544081	T	C	Substitution	intergenic_variant	-	rs1341586653
Twin_B	18	58043724	G	A	Substitution	regulatory_region_variant	-	-
Twin_B	18	61155354	C	T	Substitution	intergenic_variant	-	-
Twin_B	18	61706959	G	A	Substitution	intron_variant	*AC105094.2*	-
Twin_B	18	62562373	C	A	Substitution	downstream_gene_variant	*ZCCHC2*	-
Twin_B	18	63887009	AG	A	Deletion	intron_variant	*SERPINB2*	-
Twin_B	18	67455918	G	A	Substitution	intergenic_variant	-	-
Twin_B	18	74237595	C	T	Substitution	intron_variant	*AC090398.1*	-
Twin_B	18	75984267	C	G	Substitution	intergenic_variant	-	-
Twin_B	19	8086471	T	A	Substitution	downstream_gene_variant	*FBN3*	rs113759974
Twin_B	19	8183739	C	A	Substitution	intergenic_variant	-	rs946588717
Twin_B	19	12943750	AGCAGAG GCTTAAG GAGGAGG AAGAAGA CAAGAAA CGCAAAG AGGAGGA GGAG	A	Deletion	frameshift_variant	*CALR*	rs1555760738
Twin_B	19	16774565	G	A	Substitution	intron_variant	*NWD1*	rs577537244
Twin_B	19	17247444	C	A	Substitution	downstream_gene_variant	*USHBP1*	-
Twin_B	19	19549515	C	T	Substitution	downstream_gene_variant	*CILP2*	-
Twin_B	19	22564447	G	A	Substitution	intergenic_variant	-	rs146882932
Twin_B	19	22624713	TG	T	Deletion	downstream_gene_variant	*AC011467.2*	rs1285085532
Twin_B	19	27496424	T	C	Substitution	intergenic_variant	-	rs1568343244
Twin_B	19	28110861	A	C	Substitution	intron_variant	*AC010511.1*	-
Twin_B	19	31656627	G	A	Substitution	upstream_gene_variant	*RNA5SP471*	-
Twin_B	19	31931918	G	C	Substitution	intron_variant	*LINC01837*	-
Twin_B	19	38958665	C	G	Substitution	intron_variant	*FBXO17*	-
Twin_B	19	41492820	G	A	Substitution	downstream_gene_variant	*PCAT19*	-
Twin_B	19	47920579	G	A	Substitution	TF_binding_site_variant	-	rs367708441
Twin_B	19	53377680	A	G	Substitution	downstream_gene_variant	*ZNF525*	-
Twin_B	19	55277604	C	G	Substitution	downstream_gene_variant	*HSPBP1*	-
Twin_B	20	173236	G	A	Substitution	intergenic_variant	-	rs1034386228
Twin_B	20	1061518	G	A	Substitution	intron_variant	*AL110114.1*	-
Twin_B	20	12995529	T	C	Substitution	intron_variant	*LINC01723*	rs1438512520
Twin_B	20	14336990	T	C	Substitution	intron_variant	*FLRT3*	-
Twin_B	20	21629260	C	T	Substitution	intron_variant	*LINC01726*	rs540343176
Twin_B	20	22689135	A	T	Substitution	intron_variant	*AL158175.1*	-
Twin_B	20	28497717	AG	A	Deletion	intergenic_variant	-	rs1434446279
Twin_B	20	29525288	A	AC	Insertion	intergenic_variant	-	rs1188254666
Twin_B	20	30212896	T	G	Substitution	downstream_gene_variant	*CFTRP3*	-

Twin_B	20	30755746	C	T	Substitution	intergenic_variant	-	-
Twin_B	20	39854921	G	A	Substitution	intergenic_variant	-	rs1346211420
Twin_B	20	48547263	G	A	Substitution	intergenic_variant	-	-
Twin_B	20	50715766	T	A	Substitution	intergenic_variant	-	-
Twin_B	20	50895064	C	T	Substitution	intron_variant	*ADNP*	-
Twin_B	20	52629042	C	G	Substitution	intron_variant	*LINC01524*	-
Twin_B	20	64283610	C	G	Substitution	intron_variant	*PCMTD2*	rs1428268334
Twin_B	21	8020747	T	A	Substitution	intergenic_variant	-	rs1443418402
Twin_B	21	9928024	G	A	Substitution	intergenic_variant	-	rs1342179447
Twin_B	21	14455201	C	A	Substitution	intergenic_variant	-	-
Twin_B	21	24993236	A	G	Substitution	intron_variant	*LINC01692*	-
Twin_B	21	36182754	A	G	Substitution	intron_variant	*DOP1B*	-
Twin_B	21	40180743	A	T	Substitution	intron_variant	*DSCAM*	-
Twin_B	21	40558302	G	T	Substitution	intron_variant	*DSCAM*	-
Twin_B	21	46037210	G	A	Substitution	non_coding_transcript_ exon_variant	*AP001476.1*	-
Twin_B	22	11356944	CT	C	Deletion	intergenic_variant	-	rs1322963034
Twin_B	22	16847213	CTTAA	C	Deletion	downstream_gene_variant	*AC007064.4*	-
Twin_B	22	28259506	G	A	Substitution	intron_variant	*TTC28*	-
Twin_B	22	30635421	T	C	Substitution	TF_binding_site_variant	-	-
Twin_B	22	32657960	T	G	Substitution	intron_variant	*SYN3*	-
Twin_B	22	34618472	G	A	Substitution	intergenic_variant	-	rs867876389
Twin_B	22	34793591	T	C	Substitution	intron_variant	*LINC02885*	-
Twin_B	22	36709756	C	T	Substitution	intron_variant	*AL049749.1*	rs567131721
Twin_B	22	37961231	A	G	Substitution	intron_variant	*POLR2F*	-
Twin_B	22	42966759	C	A	Substitution	intron_variant	*PACSIN2*	-
Twin_B	22	43588922	C	T	Substitution	intron_variant	*EFCAB6*	rs567321760
Twin_B	22	48568894	G	C	Substitution	intron_variant	*TAFA5*	-
Twin_B	X	1604020	G	T	Substitution	downstream_gene_variant	*AKAP17A*	rs560737268
Twin_B	X	12284898	G	A	Substitution	intron_variant	*FRMPD4*	COSV66220435
Twin_B	X	17501537	C	A	Substitution	intron_variant	*NHS*	-
Twin_B	X	19248964	C	A	Substitution	intergenic_variant	-	-
Twin_B	X	24411967	C	T	Substitution	intergenic_variant	-	-
Twin_B	X	35556794	T	C	Substitution	intergenic_variant	-	-
Twin_B	X	35907670	T	C	Substitution	intergenic_variant	-	-
Twin_B	X	62714059	C	T	Substitution	intergenic_variant	-	rs143334744
Twin_B	X	70098264	T	A	Substitution	intergenic_variant	-	-
Twin_B	X	73335262	T	C	Substitution	intergenic_variant	-	-
Twin_B	X	78738428	T	A	Substitution	intergenic_variant	-	-
Twin_B	X	80869548	G	C	Substitution	intergenic_variant	-	-
Twin_B	X	87003014	A	G	Substitution	intergenic_variant	-	-
Twin_B	X	92949595	G	A	Substitution	intergenic_variant	-	-
Twin_B	X	107445958	C	T	Substitution	intergenic_variant	-	rs1441643081

Twin_B	X	108369773	T	A	Substitution	intron_variant	*COL4A6*	-
Twin_B	X	113433231	T	G	Substitution	intergenic_variant	-	-
Twin_B	X	115105220	C	T	Substitution	intergenic_variant	-	-
Twin_B	X	117525429	C	A	Substitution	intergenic_variant	-	-

Note: Gene annotation not provided for intergenic variants, regulatory region variants and TF binding site variants. Catalogue corresponds to the high-confidence call set prior to vafCorrect genotyping and filtering (see Methods - *Variant calling*). Gene names are provided in italics in accordance with the current HUGO Gene Nomenclature Committee (HGNC) guidelines.[50]
*An additional variant from the myeloid-gene panel is also included for Twin A (marked with an asterisk). This is a variant detected in the targeted myeloid-gene panel sequencing but with no high-confidence support from whole genome sequencing data; therefore, it was excluded from the downstream analysis.

Supplementary Table 2. Catalogue of somatic structural variants in twin A and twin B

Individual	SV_class	SV_length	Chr1	Pos1	Chr2	Pos2
Twin_A	tandem_duplication	165127	11	54384289	11	54549416
Twin_A	tandem_duplication	71117	12	11054568	12	11125685
Twin_A	tandem_duplication	75902	20	23727447	20	23803349
Twin_A	translocation	.	6	[chr6:50867003[G	12	[chr12:93869517[C
Twin_A	translocation	.	3	]chr3:11573465]A	15	T[chr15:78040845[
Twin_B	tandem_duplication	272076	11	26184499	11	26456575
Twin_B	deletion	397816	11	48727000	11	55124816
Twin_B	translocation	.	6	[chr6:43581762[C	19	[chr19:41643986[C
Twin_B	deletion	604488	2	92998101	1	93602589
Twin_B	translocation	.	5	G]chr5:248313]	5	T]chr5:1579393]

Supplementary Table 3. Summary of somatic mutations shared between twin A and twin B

#	Chr	Pos	Ref	Alt	Mutation_ type	Annotation	Shared_ annotation	Gene	Existing_ variation	CCF_in_ Twin_A	CCF_in_ Twin_B	Used_in_ Validation
1	1	237523679	G	A	Substitution	intron_ variant	High-confidence call set; vafCorrect genotyping	*RYR2*	rs1165384474	0.93	0.54	Yes
2	2	236024598	A	G	Substitution	intron_ variant	High-confidence call set; vafCorrect genotyping	*AGAP1*	-	1.05	0.99	Yes
3	3	9646697	T	A	Substitution	upstream_gene_ variant	High-confidence call set; vafCorrect genotyping	*MTMR14*	rs1001727401	0.93	1.06	No
4	3	187366417	G	A	Substitution	upstream_gene_ variant	High-confidence call set; vafCorrect genotyping	*RTP4*	-	1.01	1.16	Yes
5	5	18474168	C	T	Substitution	intergenic_ variant	High-confidence call set; vafCorrect genotyping	-	-	1.17	0.92	No
6	7	54587046	C	A	Substitution	intergenic_ variant	High-confidence call set; vafCorrect genotyping	-	-	0.89	0.37	No
7	10	103273122	G	A	Substitution	upstream_gene_ variant	High-confidence call set	*INA*	rs1286474689	0.39	0.57	No
8	16	67608941	A	G	Substitution	intron_ variant	High-confidence call set	*CTCF*	rs892105462	0.97	0.25	No
9	16	85313584	C	A	Substitution	downstream_gene_ variant	High-confidence call set; vafCorrect genotyping	*AC092275.1*	rs1483404740	1.03	1.01	Yes
10	19	12943750	AGCAG AGGCT TAAGG AGGAG GAAGA AGACA AGAAA CGCAA AGAGG AGGAG GAG	A	Deletion	frameshift_ variant	High-confidence call set	*CALR*	rs1555760738	0.69*	1.07	Yes
11	18	9601220	A	G	Substitution	intron_ variant	vafCorrect genotyping	*PPP4R1*	rs982592192, COSV53280449	0.49	0.33	No
12	2	93865725	G	C	Substitution	intergenic_ variant	vafCorrect genotyping	-	-	2	1.4	No
13	4	119161162	A	C	Substitution	intron_ variant	vafCorrect genotyping	*MYOZ2*	-	0.75	0.2	No
14	4	16908350	G	T	Substitution	intergenic_ variant; regulatory_region_ variant	vafCorrect genotyping	-	rs750655685	0.79	1.06	No
15	5	130265666	C	T	Substitution	intergenic_ variant	vafCorrect genotyping	-	rs62391477	0.61	0.68	No
16	7	71219697	C	T	Substitution	intron_ variant	vafCorrect genotyping	*GALNT17*	-	0.77	0.82	No
17	1	93828702	C	CT	Insertion	intron_ variant; regulatory_region_ variant	vafCorrect genotyping	*BCAR3*	rs969811086	0.654	0.31	No
18	2	193562677	CGT	C	Deletion	intergenic_ variant	vafCorrect genotyping	-	rs59053823	0.53	0.26	No
19	4	26343555	G	GT	Insertion	upstream_gene_ variant	vafCorrect genotyping	*RBPJ*	rs969811086	0.43	0.26	No
20	4	61895466	AAAT	A	Deletion	upstream_gene_ variant	vafCorrect genotyping	*ADGRL3*	rs969811086	0.62	0.26	No
21	6	7238595	C	CT	Insertion	intron_ variant	vafCorrect genotyping	*RREB1*	rs796560101	0.41	0.28	No
22	7	43378810	C	CA	Insertion	intron_ variant	vafCorrect genotyping	*HECW1*	rs202007736	0.5	0.23	No
23	8	17691704	G	GT	Insertion	regulatory_region_ variant	vafCorrect genotyping	-	rs200014137	0.3	0.32	No
24	9	67205531	TG	T	Deletion	intron_ variant	vafCorrect genotyping	*CNTNAP3P2*	rs368975415	0.3	0.5	No

Note: Gene annotation not provided for intergenic variants. CCF, cancer cell fraction, i.e., estimate of the fraction of tumor cells that carry the mutation. Somatic shared variants from the 'High-confidence call set' are reported in both Twin A and Twin B in Supplementary Table 1. Somatic shared variants identified after vafCorrect genotyping are reported in either Twin A or Twin B in Supplementary Table 1 (see Methods - *Variant calling* and *Mutation clustering*). Gene names are provided in italics

in accordance with the current HUGO Gene Nomenclature Committee (HGNC) guidelines.[50] *95% confidence interval for *CALR* CCF: 0.42-1.

Supplementary Table 4. Summary of non-silent coding variants in twin A and twin B

**Individual**	**Chr**	**Pos**	**Ref**	**Alt**	**Mutation_ type**	**Annotation**	**Impact**	**Gene**	**Existing_ variation**	**Clinical_ significance**	**CADD_ PHRED**
Twin_A	1	53327841	CA	C	Deletion	frameshift_ variant	HIGH	*LRP8*	rs761955852, COSV60099910	-	22
Twin_A	4	105234449	TA	T	Deletion	frameshift_ variant	HIGH	*TET2*	-	-	-
Twin_A	4	105259633*	G	A	Substitution	missense_ variant	MODERATE	*TET2*	COSV54404036	pathogenic	27.2
Twin_A	11	17634218	C	T	Substitution	stop_gained	HIGH	*OTOG*	rs866476223	pathogenic	40
Twin_A	19	12943750	AGCAG AGGCT TAAGG AGGAG GAAGA AGACA AGAAA CGCAA AGAGG AGGAG GAG	A	Deletion	frameshift_ variant	HIGH	*CALR*	rs1555760738	pathogenic	35
Twin_B	9	131860557	T	A	Substitution	missense_ variant	MODERATE	*MED27*			31
Twin_B	10	24984824	G	A	Substitution	missense_ variant	MODERATE	*ENKUR*	rs200773323, COSV58633510	-	25.9
Twin_B	12	88071791	G	A	Substitution	missense_ variant	MODERATE	*CEP290*	rs761572735	-	25.1
Twin_B	14	62950522	C	T	Substitution	missense_ variant	MODERATE	*KCNH5*	rs587777164, CM1310653, COSV59758318, COSV59758899	uncertain_ significance, pathogenic	28
Twin_B	19	12943750	AGCAG AGGCT TAAGG AGGAG GAAGA AGACA AGAAA CGCAA AGAGG AGGAG GAG	A	Deletion	frameshift_ variant	HIGH	*CALR*	rs1555760738	pathogenic	35

Note: Gene names are provided in italics in accordance with the current HUGO Gene Nomenclature Committee (HGNC) guidelines.[50] *An additional variant from the myeloid-gene panel is also included for Twin A (marked with an asterisk). This is a variant detected in the targeted myeloid-gene panel sequencing but with no high-confidence support from whole genome sequencing data; therefore, it was excluded from the downstream analysis.

Supplementary Table 5. Data for time to the most recent common ancestor (MRCA) estimation

(a) Using N[C>T]pG mutation count data

	mean	se_mean	SD	95%_CI_lower_ confidence _limit	95%_CI_upper_ confidence _limit	n_eff	Rhat
t_{MRCA}	0.95	0.00	0.54	0.20	2.25	287761.60	1.0000
t[1]	2.80	0.00	0.15	2.453	2.99	211004.80	1.0000
t[2]	31.87	0.01	3.37	25.329	38.16	188785.40	1.0000
rate	36.69	0.00	1.93	32.035	39.30	294264.00	1.0000
lp__	732.06	0.01	1.64	727.945	734.12	106813.70	1.0000
Bridge sampling estimate of the log marginal likelihood: -10.93478; Relative Mean-Squared Error: 1.756122e-06.							

(b) Using lineage-restricted SNV count data

	mean	se_mean	SD	95%_CI_lower_ confidence _limit	95%_CI_upper_ confidence _limit	n_eff	Rhat
t_{MRCA}	2.16	0.00	0.98	0.61	4.37	78069.74	1.0000
t[1]	4.12	0.01	2.06	2.427	9.95	44277.66	1.0001
t[2]	25.22	0.03	7.53	9.059	36.49	51312.44	1.0000
rate	24.97	0.03	7.50	8.952	36.41	51542.02	1.0000
lp__	644.68	0.01	1.65	640.584	646.79	77872.47	1.0001
Bridge sampling estimate of the log marginal likelihood: -9.01966; Relative Mean-Squared Error: 9.335533e-06.							

Note: t_{MRCA} is an estimate of time (years) between fertilization and the branch point of MPN lineages in twin A and B. t[1] and t[2] are estimates of the time (years) to the MRCA of cells in twin A and twin B. lp__ corresponds to the logarithm of the (unnormalized) posterior density as calculated by Stan. n_eff is a crude measure of effective sample size and 'Rhat' is the potential scale reduction factor on split chains (Rhat=1 at convergence). se_mean is the estimated standard error of the mean of the simulations. rate is the (a) N[C>T]pG (b) clonal mutation rate per genome per year. SNV, single-nucleotide variant.

Supplementary Table 6. Summary of the shared somatic variants and unique somatic variants for each twin, used in whole genome sequencing validation at the single-cell level

Variant studied	Chr	Pos	Ref	Alt	Mutation_ type	Annotation	Nearest_ gene	Existing_ variation	VAF_ Twin_A	VAF_ Twin_B
somatic_ shared	19	12943750	AGCAGAGGC TTAAGGAGG AGGAAGAAG ACAAGAAAC GCAAAGAGG AGGAGGAG	A	Deletion	frameshift_ variant	*CALR*	rs1555760738	_	_
somatic_ shared_1	1	237523679	G	A	Substitution	intron_ variant	*RYR2*	rs1165384474	0.32	
somatic_ shared_2	2	236024598	A	G	Substitution	intron_ variant	*AGAP1*	-	0.41	
somatic_ shared_3	3	187366417	G	A	Substitution	upstream_ gene_ variant		-	0.39	
somatic_ shared_4	16	85313584	C	A	Substitution	downstream_ gene_ variant	*AC092275.1*	rs1483404740	0.36	
somatic_ unique_ Twin_A_1	10	18976904	C	T	Substitution	Intergenic_ variant		rs181225799	0.31	NA
somatic_ unique_ Twin_A_2	11	78991748	C	T	Substitution	intron_ variant	*TENM4*	.	0.29	NA
somatic_ unique_ Twin_B_1	7	116155279	G	C	Substitution	intergenic_ variant		rs1461424202	NA	0.19
somatic_ unique_ Twin_B_2	8	20863668	T	A	Substitution	intergenic_ variant		-	NA	0.23

Note: Gene names are provided in italics in accordance with the current HUGO Gene Nomenclature Committee (HGNC) guidelines.[50] VAF, variant allele frequency.

Supplementary Table 7. Overview of patient clinical information for the JAK2V617F-mutant myeloproliferative neoplasm cohort

Sample	Gender	MPN Dx	Age at Dx (years)	Driver mutation	Driver mutation VAF at Dx (%)	BMAT	Cytogenetics	NGS myeloid-gene panel	*JAK2V617F* detection on DBS (Y/N)
DBS_1	Female	SM	31.9	*KITD816V*	NK	SM	NA	Nil else	N
DBS_2	Male	PV	28.9	*JAK2V617F*	16.0	NA	NA	NA	N
DBS_3	Female	PV	32.7	*JAK2V617F*	65.0	PV	Normal; 46,XX[20]	Nil else	N
DBS_4	Male	PV	34.1	*JAK2V617F*	28.0	NA	NA	Nil else	Y

Note: Gene names are provided in italics in accordance with the current HUGO Gene Nomenclature Committee (HGNC) guidelines.[50] BMAT, bone marrow aspirate and trephine; DBS, neonatal dried blood spot; Dx, diagnosis; MPN, myeloproliferative neoplasm; NGS, next-generation sequencing; NK, not known; PV, polycythemia vera; SM, systemic mastocytosis; VAF, variant allele frequency.

Supplementary Table 8. Flow cytometry and cell sorting panels used in granulocyte, T cell and HSPC analysis and sorting

**(a) Granulocyte panel**					
**Antigen**	**Clone**	**Conjugate**	**Dilution**	**Company**	**Catalogue_number**
CD3	SK7	FITC	5:100	BD Biosciences[§]	345763
CD34	4HI1	APC-eFluor® 780	2:100	eBioscience™[#]	47-0349-42
CD71	M-A712	Alexa Fluor® 700	5:100	BD Biosciences[§]	563769
CD19	HIB19	V450	5:100	BD Biosciences[§]	560354
7AAD (7-Aminoactinomycin D)			1:100*	Cayman Chemical[†]	11397
CD11b	ICRF44	APC	5:100	eBioscience™[#]	17-0118-42
CD14	61D3	APC	5:100	eBioscience™[#]	17-0149-42
CD33	WM53	PE	5:100	BioLegend[‡]	303404
**(b) T cell panel**					
**Antigen**	**Clone**	**Conjugate**	**Dilution**	**Company**	**Catalogue_number**
CD3	SK7	FITC	5:100	BD Biosciences[§]	345763
CD4	SK3	APC	5:100	BD Pharmingen™[§]	565994
DAPI (4',6-Diamidino-2-Phenylindole, Dilactate)			1:100*	Invitrogen™[#]	D3571
CD19	HIB19	APC-Cyanine7	5:100	BioLegend[‡]	302218
CD11b	ICRF44	PE-Cyanine5	5:100	BioLegend[‡]	301308
CD14	61D3	PE-Cyanine5	5:100	eBioscience™[#]	15-0149-42
**(c) HSPC panel**					
**Antigen**	**Clone**	**Conjugate**	**Dilution**	**Company**	**Catalogue_number**
CD34	4HI1	APC-eFluor® 780	0.66:100	eBioscience™[#]	47-0349-42
CD38	HIT2	PE-Texas Red®	4.66:100	Invitrogen™[#]	MHCD3817
CD45RA	HI100	PE	0.66:100	BioLegend[‡]	304108
CD90	5E10	Brilliant Violet 421™	3.33:100	BioLegend[‡]	328122
CD123	6H6	PE-Cyanine7	1.66:100	BioLegend[‡]	306010
7AAD (7-Aminoactinomycin D)			1:100*	Cayman Chemical[†]	11397
Lineage mix			21.66:100		
CD8a^	RPA-T8	FITC	1:100 of Lineage mix	BioLegend[‡]	301006
CD10^	HI10a	FITC	3.33:100 of Lineage mix	BioLegend[‡]	312208
CD20^	2H7	FITC	0.66:100 of Lineage mix	BioLegend[‡]	302304
CD66b^	G10F5	FITC	6.66:100 of Lineage mix	BioLegend[‡]	305104
CD127^	eBioRDR5	FITC	3.33:100 of Lineage mix	eBioscience™[#]	11-1278-52
Human Hematopoietic Lineage Cocktail (CD2, CD3, CD14, CD16, CD56, CD235a) ^	RPA-2.10, OKT3, 61D3, CB16, HIB19, TULY56, HIR2	FITC	6.66:100 of Lineage mix	eBioscience™[#]	22-7778-72

*Viability stains were previously diluted 1:200 for 7-AAD and 1:100 for DAPI from their 5mg/ml stock solutions. ^Lineage mix. [§]Becton, Dickinson and Company, Franklin Lakes, US-NJ; [#]Thermo Fisher Scientific Inc., Waltham, US-MA; [†]Cayman Chemical Company, Ann Arbor, US-MI; [‡]BioLegend, San Diego, US-CA. HSPC, hematopoietic stem and progenitor cell.

Supplementary Table 9. Catalogue of oligonucleotides used in the polymerase chain reaction assays

Target nucleotide(s)	Nearest gene	Forward_primer_sequence (5' → 3')	Reverse_primer_sequence (5' → 3')	Probe_sequence	Product size (bp)	Company
chr19:12943750-1294801	*CALR*	GCAGCAGAGAAACAAATG	GCCTCTCTACAGCTCGTCCTT		207	Invitrogen™
chr4:105234449	*TET2*	ACGTAACTTCGGGGTAAGCC	TCCACGGAAGAGGCAGAAAC		291	Invitrogen™
chr4:105259633	*TET2*	tgtgtggttatgccacagctt	acatcaggaagtaaacaaacCTCT		312	Invitrogen™
chr1:237523679	*RYR2*	ccagctgggagagaatatttgtga	ggagctgctaaaggttattctgc		1300	Invitrogen™
chr2:236024598	*AGAP1*	ttgtccttttccccagaccc	catgaatccgggctgtcaaac		259	Invitrogen™
chr3:187366417	_	gaatttaagccccaggaggaca	ccagtgtcagaggaaccgtag		268	Invitrogen™
chr16:85313584	*AC092275.1*	tccagcgtgattccgtgttt	ttctgaccgcccagactttg		346	Invitrogen™
chr10:18976904	_	aactcaacccttgtccccatc	cttttccctcccactgacaca		213	Invitrogen™
chr11:78991748	*TENM4*	gcacttgttgaatgcttgtgc	ggtctgggcccacatttctt		390	Invitrogen™
chr7:116155279	_	ttctagcccctgctatgcac	ggcccaatggcacagtaaga		452	Invitrogen™
chr8:20863668	_	gccttcgattggggctgttta	aaggggttctcttccagcaaa		441	Invitrogen™
chr9:5073770	*JAK2*	aggcttacacaggggtttcc	ttgtttgggcattgtaaccttc		505	Life Technologies™
chr9:5073770	*JAK2*	GCTTTCTCACAAGCATTTG	gcattagaaagcctgtagtttta		84	Thermo Fisher Scientific
chr9:5073770(G)	*JAK2*			[HEX]TCGTCTCCACAGACACA TACTCCATGAGACGA[BHQ1]		Merck
chr9:5073770(T)	*JAK2*			[6FAM]TCGTCTCCACAGAAAC ATACTCCATGAGACGA[BHQ1]		Merck

Note: All target coordinates according to the Genome Reference Consortium Human Build 38 (GRCh38). All primers were checked against RefSeq and human genome assembly databases using NCBI Primer-BLAST® (National Library of Medicine, Bethesda, US-MD) as well as SnapGene® Viewer, v5.3 (GSL Biotech LLC, Chicago, US-IL). The ddPCR assay probes were labelled with HEX and FAM for wild-type *JAK2* and the NM_004972.3[JAK2]:c.1849G>T p.V617F, respectively. Gene names are provided in italics in accordance with the current HUGO Gene Nomenclature Committee guidelines.[50] Uppercase letters are used for exonic regions, while lowercase letters represent intronic regions. Invitrogen™, Life Technologies™, Thermo Fisher Scientific: Thermo Fisher Scientific, Waltham, US-MA; Merck: Merck Group, Darmstadt, DE. ddPCR, droplet digital polymerase chain reaction.

Supplementary Table 10. Catalogue of the genes targeted in the next-generation sequencing panel

Gene	RefSeq_accession_number
ABL1	NM_005157; NM_007313
ANKRD26	NM_001256053; NM_014915
ASXL1	NM_015338
ATRX	NM_000489
BCOR	NM_001123383; NM_017745
BCORL1	NM_021946; NM_001184772
BRAF	NM_004333
BRCC3	NM_024332
CALR	NM_004343
CBL	NM_005188
CBLB	NM_170662
CBLC	NM_012116
CDKN2A	NM_001195132; NM_000077; NM_058195
CEBPA	NM_004364
CHEK2	NM_001005735; NM_007194
CSF3R	NM_156039
CUX1	NM_001202543; NM_181552
DDX41	NM_016222
DNMT3A	NM_022552; NM_175629
ETV6	NM_001987
EZH2	NM_004456
FBXW7	NM_033632; NM_018315
FLT3	NM_004119
GATA1	NM_002049
GATA2	NM_032638; NM_001145661
GNAS	NM_000516; NM_001077490
HRAS	NM_005343; NM_001130442
IDH1	NM_005896; NM_001282386; NM_001282387
IDH2	NM_002168
IKZF1	NM_001220765; NM_006060
JAK2	NM_004972; NM_001322194; NM_001322195; NM_001322196
JAK3	NM_000215
KDM6A	NM_021140
KIT	NM_000222
KRAS	NM_033360
KMT2A	NM_001197104; NM_005933
MPL	NM_005373
MYD88	NM_001172567

NF1	NM_000267
NFE2	NM_001136023; NM_001261461; NM_006163
NOTCH1	NM_017617
NPM1	NM_002520; NM_001355006
NRAS	NM_002524
PDGFRA	NM_006206
PHF6	NM_032458; NM_001015877
PPM1D	NM_003620
PTEN	NM_000314
PTPN11	NM_002834
RAD21	NM_006265
RUNX1	NM_001754
SETBP1	NM_015559
SETD2	NM_014159
SETDB1	NM_001145415
SF3B1	NM_012433
SH2B3	NM_005475
SMC1A	NM_006306
SMC3	NM_005445
SRSF2	NM_001195427; NM_003016
STAG1	NM_005862
STAG2	NM_001042749; NM_006603; NM_001282418
STAT3	NM_139276
STAT5B	NM_012448
TET2	NM_001127208
TP53	NM_000546; NM_001126112
U2AF1	NM_001025203
WT1	NM_024426
ZRSR2	NM_005089

Note: Gene names are provided in italics in accordance with the current HUGO Gene Nomenclature Committee (HGNC) guidelines.[50]

Supplementary Table 11. Summary of multidimensional mutation clustering

(a) Summary of mutation assignments

Cluster_no	CCF_in_Twin_A	CCF_in_Twin_B	Estimated_no_of_mutations	No_of_mutations_assigned
1	0.000	0.315	452.348	452
2	0.359	0.000	425.794	426
3	0.898	1.003	9.859	10

(b) Individual mutation assignments

Most_likely_cluster	Chr	Mutation_start	Twin_A_Subclonal_Fraction	Twin_B_Subclonal_Fraction
1	1	104033383	0.000	0.300
2	1	106288464	0.254	0.000
1	1	110190300	0.000	0.674
1	1	110738023	0.000	0.434
2	1	111854251	0.167	0.000
2	1	112723366	0.444	0.000
1	1	114307529	0.000	0.271
1	1	12823937	0.000	0.271
1	1	143898748	0.000	0.643
1	1	147277076	0.000	0.362
2	1	152011365	0.291	0.000
2	1	152568644	0.444	0.000
2	1	155442289	0.342	0.044
2	1	155643901	0.838	0.000
1	1	157062690	0.000	0.251
1	1	158329018	0.000	0.643
1	1	159742120	0.000	0.334
2	1	164325871	0.392	0.000
1	1	165976313	0.000	0.181
1	1	166242296	0.000	0.322
2	1	172874706	0.296	0.000
2	1	173897024	0.272	0.000
1	1	175165067	0.000	0.241
1	1	176214397	0.000	0.470
1	1	177128801	0.000	0.478
2	1	17778871	0.190	0.000
2	1	178136491	0.747	0.000
2	1	185587702	0.644	0.000
2	1	185717034	0.533	0.000
1	1	187326523	0.000	0.217
2	1	188790583	0.491	0.000
1	1	190010459	0.000	0.507

2	1	19011134	0.508	0.000
2	1	190678569	0.503	0.000
2	1	191963370	0.471	0.000
1	1	194805882	0.000	0.329
1	1	1948293	0.000	0.592
2	1	196771008	0.667	0.000
2	1	199078846	0.368	0.101
1	1	211364253	0.000	0.247
1	1	21195179	0.000	0.931
1	1	216808720	0.000	0.310
2	1	218758531	0.410	0.000
1	1	228759153	0.000	0.724
1	1	231661954	0.000	0.310
1	1	239287292	0.000	0.140
1	1	239790569	0.000	0.354
3	1	54128162	0.966	0.909
1	1	57203495	0.000	0.322
2	1	58846527	0.500	0.000
1	1	68690432	0.000	0.869
2	1	81244570	0.339	0.000
2	1	82141981	0.267	0.043
1	1	90494264	0.000	0.661
2	1	91064439	1.191	0.000
2	1	91598893	0.410	0.000
2	1	93828702	0.654	0.310
1	2	103642003	0.000	0.194
1	2	104572208	0.000	0.078
2	2	105464070	0.291	0.000
1	2	110152812	0.000	0.350
1	2	112972342	0.000	0.212
2	2	113030007	0.632	0.000
1	2	113360949	0.000	0.000
1	2	114347202	0.000	0.000
2	2	11568288	0.182	0.263
1	2	122468457	0.000	0.000
2	2	122548088	0.333	0.496
2	2	124139117	0.288	0.000
2	2	12436887	0.381	0.207
1	2	124707645	0.000	0.000
1	2	125662877	0.000	0.217
2	2	126802154	0.453	0.000
2	2	13111603	0.427	0.000

2	2	135703624	0.308	0.217
1	2	136809746	0.000	0.601
1	2	139215973	0.000	0.651
2	2	139236448	0.356	0.290
1	2	139897503	0.000	0.000
2	2	140606025	0.522	0.000
2	2	142582383	0.140	0.362
1	2	142622241	0.000	0.142
2	2	145569408	0.381	0.217
2	2	145603291	0.200	0.371
1	2	147580489	0.000	0.000
2	2	148242718	0.444	0.496
1	2	14870316	0.000	0.000
2	2	153181357	0.533	0.383
1	2	156749565	0.000	0.231
1	2	158735231	0.000	0.000
2	2	163100502	0.395	0.054
2	2	167809460	0.281	0.416
2	2	174496556	0.471	0.000
2	2	175727129	0.556	0.000
2	2	17632861	0.054	0.000
1	2	17816780	0.000	0.189
2	2	181542157	0.222	0.000
1	2	181754253	0.000	0.290
2	2	184966	0.519	0.000
1	2	186809029	0.000	0.000
1	2	187978655	0.000	0.434
1	2	190310023	0.000	0.290
2	2	193562677	0.533	0.117
2	2	19486986	0.494	0.000
2	2	196497946	0.344	0.000
1	2	197588962	0.000	0.354
2	2	199505098	0.296	0.449
2	2	199984230	0.317	0.000
2	2	200975338	0.333	0.434
1	2	202941148	0.000	0.000
1	2	214886582	0.000	0.695
1	2	215762883	0.000	0.062
1	2	21701231	0.000	0.000
2	2	217649629	0.533	0.620
1	2	22021980	0.000	0.000
1	2	220248897	0.000	0.455

2	2	221924308	0.406	0.248
1	2	222991555	0.000	0.197
1	2	22585137	0.000	0.465
1	2	22585139	0.000	0.571
1	2	226321782	0.000	0.000
2	2	227692889	0.325	0.000
1	2	22994571	0.000	0.462
2	2	235048421	0.421	0.395
2	2	235142193	0.133	0.347
3	2	236024598	1.259	0.597
2	2	236165626	0.417	0.494
1	2	238340846	0.000	0.265
1	2	238788248	0.000	0.000
1	2	24019295	0.000	0.000
2	2	24266260	0.533	0.176
2	2	25819904	0.351	0.698
1	2	27314362	0.000	0.300
1	2	28287298	0.000	0.000
1	2	29298533	0.000	0.000
2	2	2982012	0.444	0.000
2	2	29907671	0.184	0.400
2	2	31671794	0.320	0.000
1	2	33204005	0.000	0.000
1	2	34609950	0.000	0.229
2	2	35209815	0.222	0.261
1	2	39543295	0.000	0.000
1	2	41851787	0.000	0.338
2	2	5026216	0.454	0.000
1	2	5809965	0.000	0.000
1	2	76343534	0.000	0.000
1	2	77138659	0.000	0.000
1	2	77305966	0.000	0.000
2	2	78265814	0.356	0.000
1	2	7937210	0.000	0.106
1	2	79741961	0.000	0.000
1	2	84976628	0.000	0.606
2	2	90026936	0.866	0.362
2	2	98022727	0.364	0.302
1	3	101873401	0.000	0.000
2	3	103475498	0.808	0.253
2	3	107699193	0.571	0.511
1	3	107880323	0.000	0.000

1	3	111393544	0.000	0.371
2	3	118298534	0.296	0.000
1	3	120551312	0.000	0.494
1	3	124318634	0.000	0.000
2	3	1259968	0.390	0.308
2	3	126102405	0.356	0.368
1	3	127184194	0.000	0.064
1	3	146968300	0.000	0.000
1	3	148123392	0.000	0.465
1	3	150872501	0.000	0.352
1	3	152516100	0.000	0.000
2	3	152974761	0.392	0.111
2	3	153068733	0.491	0.339
2	3	153706759	0.340	0.217
1	3	157178549	0.000	0.390
2	3	160115640	0.578	0.310
1	3	169022850	0.000	0.225
1	3	182077482	0.000	0.334
3	3	187366417	1.333	0.000
1	3	192580552	0.000	0.000
1	3	197965767	0.000	0.000
2	3	26440532	0.267	0.000
2	3	29763656	0.216	0.339
2	3	32019778	0.342	0.420
2	3	32526653	0.610	0.000
1	3	39756857	0.000	0.229
1	3	50879771	0.000	0.000
1	3	52975648	0.000	0.140
1	3	54092594	0.000	0.000
1	3	57095586	0.000	0.140
1	3	59401686	0.000	0.000
2	3	59893891	0.242	0.000
1	3	62217141	0.000	0.000
1	3	6268058	0.000	0.310
2	3	65074311	0.702	0.310
2	3	72405852	0.566	0.434
1	3	74463202	0.000	0.000
2	3	74980380	0.229	0.376
2	3	76086971	0.305	0.597
2	3	80858231	0.508	0.326
1	3	8179865	0.000	0.661
1	3	8330619	0.000	0.000

1	3	84248079	0.000	0.241
2	3	86921756	0.218	0.133
1	3	8749340	0.000	0.170
1	3	8766880	0.000	0.724
1	3	8800977	0.000	0.181
1	3	89748810	0.000	0.449
2	3	90430217	0.821	0.496
1	3	95223077	0.000	0.526
3	3	9646697	0.948	0.000
1	3	96820605	0.000	0.207
2	4	105234449	0.190	0.000
2	4	106394908	0.444	0.414
1	4	108394132	0.000	0.424
2	4	109876682	0.533	0.000
2	4	111359412	0.600	0.300
1	4	115259241	0.000	0.000
2	4	119161162	0.747	0.000
2	4	119833655	0.389	0.000
1	4	120599980	0.000	0.000
2	4	122600220	0.519	0.000
1	4	125617334	0.000	0.000
1	4	126647636	0.000	0.000
1	4	127771889	0.000	0.000
2	4	134850961	0.381	0.434
1	4	140154007	0.000	0.000
1	4	144879760	0.000	0.000
1	4	147616580	0.000	0.000
1	4	152098840	0.000	0.000
2	4	160621868	0.410	0.554
2	4	161252551	0.163	0.521
2	4	161288198	0.397	0.362
1	4	161716379	0.000	0.059
2	4	162816440	0.549	0.280
1	4	166613505	0.000	0.000
3	4	16908350	0.789	0.651
1	4	174170616	0.000	0.167
1	4	179639868	0.000	0.171
1	4	180135661	0.000	0.261
2	4	180265089	0.455	0.056
2	4	183453115	0.089	0.390
1	4	184638048	0.000	0.000
2	4	187661068	0.099	0.155

2	4	188628950	0.516	0.334
2	4	22103283	0.284	0.150
2	4	26343555	0.432	0.404
1	4	26875683	0.000	0.070
2	4	26935882	0.144	0.217
2	4	3011910	0.381	0.271
2	4	31169536	0.323	0.163
1	4	33615886	0.000	0.170
2	4	35852584	0.316	0.383
1	4	37418639	0.000	0.271
1	4	39429436	0.000	0.434
2	4	44413264	0.296	0.000
1	4	46668719	0.000	0.402
1	4	47220483	0.000	0.000
1	4	52190355	0.000	0.235
2	4	52545084	0.491	0.630
2	4	5718308	0.310	0.000
2	4	61895466	0.615	0.000
1	4	64216296	0.000	0.352
1	4	6451943	0.000	0.339
1	4	64665477	0.000	0.668
2	4	69630424	0.373	0.000
1	4	70520668	0.000	0.258
1	4	71648848	0.000	0.000
2	4	74668604	0.505	0.034
1	4	76047967	0.000	0.000
2	4	82531664	0.237	0.000
1	4	86440642	0.000	0.000
1	4	88387262	0.000	0.000
2	5	100034257	0.381	0.000
2	5	100071988	0.274	0.265
2	5	100155088	0.471	0.000
2	5	100295778	0.649	0.000
1	5	105561137	0.000	0.323
2	5	105941453	0.296	0.286
1	5	106685060	0.116	0.189
2	5	10937820	0.360	0.053
1	5	110479443	0.000	0.000
2	5	111449354	0.323	0.679
1	5	11361065	0.000	0.000
2	5	115666098	0.308	0.177
2	5	116208129	0.276	0.300

2	5	116797618	0.340	0.000
1	5	122619069	0.000	0.592
2	5	123223704	0.278	0.217
2	5	12387040	0.471	0.000
2	5	131362688	0.310	0.000
2	5	131622186	0.437	0.000
1	5	137416371	0.000	0.000
1	5	140836906	0.000	0.407
1	5	147046232	0.000	0.814
2	5	147611565	0.058	0.000
2	5	152460283	0.260	0.395
2	5	155157242	0.547	0.973
1	5	155317926	0.000	0.000
1	5	157005759	0.000	1.025
1	5	159980907	0.000	0.000
1	5	161395724	0.000	0.532
1	5	161830310	0.000	0.323
2	5	162912266	0.267	0.461
2	5	163087087	0.333	0.362
2	5	166031221	0.284	0.000
1	5	175182485	0.000	0.217
1	5	178484296	0.000	0.334
3	5	18474168	0.889	0.000
1	5	19703201	0.000	0.000
2	5	2114089	0.561	0.223
1	5	30228903	0.000	0.159
1	5	32183802	0.000	0.362
2	5	33779372	0.342	0.189
1	5	35163531	0.000	0.000
2	5	35831884	0.909	0.370
2	5	36636178	0.516	0.543
1	5	41232152	0.000	0.322
1	5	41663190	0.000	0.000
1	5	42222760	0.000	0.197
1	5	45194560	0.000	0.296
2	5	4579966	0.545	0.000
1	5	53160317	0.000	0.310
2	5	56387833	0.368	0.271
1	5	5721155	0.000	0.407
2	5	63548792	0.577	0.000
2	5	6643035	0.421	0.000
2	5	69055388	0.372	0.263

1	5	72404389	0.000	0.000
1	5	7425190	0.000	0.000
2	5	75285945	0.513	0.078
2	5	7817145	0.327	0.000
2	5	79269736	0.533	0.271
2	5	81377269	0.424	0.310
1	5	85129366	0.000	0.235
2	5	85688616	0.741	0.000
2	5	91742536	0.593	0.000
1	5	9500811	0.000	0.000
2	5	95231337	0.392	0.000
2	5	95701747	0.715	0.280
2	5	97765568	0.201	0.000
1	6	101437584	0.000	0.094
1	6	102787939	0.000	0.000
2	6	103570525	0.381	0.362
1	6	105574693	0.000	0.629
1	6	110560428	0.000	0.362
2	6	111252036	0.485	0.000
1	6	115939145	0.000	0.620
2	6	117652832	0.181	0.255
2	6	123368975	0.092	0.000
2	6	124380080	0.800	0.000
2	6	126389883	0.086	0.519
2	6	131059163	0.351	0.000
2	6	131399570	0.406	0.372
1	6	132482937	0.000	0.064
2	6	145547033	0.410	0.062
1	6	153868755	0.000	0.329
1	6	155942473	0.000	0.000
1	6	1850433	0.000	1.067
1	6	18553216	0.000	0.000
1	6	20182475	0.000	0.223
1	6	23725753	0.000	0.643
1	6	23820823	0.000	0.000
1	6	33355096	0.000	0.197
1	6	40322738	0.000	0.103
2	6	40934220	0.638	0.000
1	6	45795560	0.000	0.362
2	6	46776061	0.232	0.000
2	6	46995372	0.406	0.827
1	6	49245413	0.000	0.000

1	6	49866189	0.000	0.280
2	6	60497665	0.208	0.000
2	6	62533854	0.585	0.445
2	6	70161977	0.558	0.000
2	6	71671466	0.067	0.322
2	6	7238595	0.415	0.000
1	6	73054903	0.000	0.362
1	6	78332113	0.000	0.526
1	6	80558206	0.000	0.543
2	6	9330828	0.267	0.109
2	6	93696846	0.310	0.290
2	6	95778674	0.274	0.326
1	7	10181486	0.000	0.000
2	7	102065090	0.301	0.452
1	7	108865841	0.000	0.255
2	7	110615318	0.464	0.000
1	7	111235913	0.000	0.318
1	7	116155279	0.000	0.000
2	7	122016458	0.381	0.043
2	7	124142825	0.323	0.000
1	7	134178896	0.000	0.322
2	7	137296579	0.417	0.386
1	7	137739877	0.000	0.000
2	7	139519359	0.211	0.000
1	7	142637901	0.000	0.099
2	7	153528906	0.424	0.758
2	7	154611485	0.305	0.383
2	7	156931430	0.184	0.000
1	7	20905885	0.000	0.266
1	7	26002719	0.000	0.161
1	7	27125974	0.000	0.000
1	7	30124532	0.000	0.192
2	7	30831221	0.261	0.000
1	7	33443171	0.000	0.000
1	7	33920177	0.000	0.661
2	7	41732610	0.434	0.000
2	7	43378810	0.503	0.362
1	7	43433707	0.000	0.000
1	7	4358841	0.000	0.558
1	7	44053423	0.000	0.164
2	7	44090851	0.261	0.000
1	7	45053757	0.000	0.000

1	7	48916465	0.000	0.000
2	7	49269510	0.296	0.000
2	7	49269512	0.296	0.000
2	7	4979367	0.308	0.255
2	7	53091502	0.627	0.000
2	7	54587046	0.978	0.494
2	7	60485855	0.229	0.000
1	7	65590326	0.000	0.300
1	7	67521209	0.000	0.371
2	7	69630257	0.305	0.472
2	7	7056369	0.352	0.259
3	7	71219697	0.770	0.000
1	7	71503989	0.000	0.000
1	7	7383076	0.000	0.496
1	7	75784737	0.000	0.000
1	7	86130259	0.000	0.000
2	7	88505688	0.372	0.000
2	7	90565456	0.232	0.465
2	7	9227657	0.571	0.302
2	7	95205017	0.547	0.461
2	7	95298520	0.314	0.528
2	7	96055652	0.296	0.000
2	7	98561959	0.421	0.181
2	8	104362862	0.444	0.606
2	8	108043942	0.356	0.000
1	8	111647834	0.000	0.101
1	8	114563854	0.000	0.535
1	8	116551195	0.000	0.451
2	8	117615713	0.480	0.388
1	8	122234933	0.000	0.000
2	8	124975564	0.065	0.478
2	8	129273738	0.281	0.000
2	8	129372882	0.567	0.049
2	8	129733477	0.261	1.022
1	8	131552936	0.000	0.000
1	8	133562727	0.000	0.418
2	8	135261370	0.549	0.543
1	8	136497219	0.000	0.225
2	8	17691704	0.296	0.000
2	8	19550828	0.480	0.000
1	8	19798556	0.000	0.310
1	8	20863668	0.000	0.148

1	8	25151260	0.000	0.247
2	8	25892225	0.325	0.000
1	8	26940513	0.000	0.000
2	8	30978684	0.424	0.000
1	8	32400294	0.000	0.231
1	8	32524703	0.000	0.148
1	8	3391860	0.000	0.000
1	8	34194743	0.000	0.517
2	8	36379501	0.351	0.326
2	8	36570758	0.444	0.000
1	8	41188472	0.000	0.362
1	8	43877252	0.000	0.310
1	8	46315152	0.000	0.352
2	8	48680993	0.711	0.159
1	8	49102414	0.000	0.000
1	8	4997791	0.000	0.248
1	8	50264084	0.000	0.362
2	8	51711248	0.388	0.207
1	8	53427578	0.000	0.348
1	8	57557945	0.000	0.000
1	8	5826762	0.000	0.225
1	8	58506466	0.000	0.000
1	8	59792693	0.000	0.163
2	8	63967261	0.327	0.585
1	8	64469993	0.000	0.418
1	8	924933	0.000	0.090
1	8	98657310	0.000	0.000
2	9	101191229	0.218	1.048
1	9	10229559	0.000	0.000
1	9	102339974	0.000	0.000
2	9	102487319	0.690	0.290
1	9	102993672	0.000	0.434
1	9	103368304	0.000	0.560
1	9	104257777	0.000	0.163
1	9	104331598	0.000	0.000
1	9	105298502	0.000	0.000
2	9	111554985	0.274	0.189
2	9	118247951	0.086	0.000
1	9	118261593	0.000	0.117
2	9	120700338	0.604	0.000
2	9	122872477	0.491	0.139
1	9	124851965	0.000	0.000

2	9	125539853	0.254	0.000
1	9	12626664	0.000	0.000
2	9	127663224	0.174	0.000
1	9	12964481	0.000	0.000
2	9	131190221	0.698	0.255
1	9	131860557	0.000	0.000
2	9	14104419	0.519	0.323
2	9	1822815	0.511	0.630
2	9	19348792	0.410	0.114
1	9	21096138	0.000	0.965
1	9	23036773	0.000	0.819
2	9	23699353	0.250	0.000
1	9	24723875	0.000	0.372
1	9	24924904	0.000	0.000
2	9	28150104	0.235	0.217
1	9	38292964	0.000	0.000
2	9	395970	0.404	0.420
1	9	42290039	0.000	0.136
2	9	4316936	0.622	0.000
2	9	50071	0.143	0.000
1	9	5583708	0.000	0.338
1	9	595757	0.000	0.374
2	9	61566797	0.401	0.000
2	9	73087756	0.610	0.000
1	9	73452437	0.000	0.310
1	9	74317541	0.000	0.068
2	9	77004114	0.808	0.000
2	9	83484424	0.322	0.724
2	9	84858874	0.267	0.452
1	9	88848472	0.000	0.000
1	9	9208774	0.000	0.000
1	9	95722226	0.000	0.489
1	9	98676266	0.000	0.869
1	10	102661031	0.000	0.370
2	10	10849842	0.410	0.362
2	10	109091857	0.485	0.278
1	10	10933457	0.000	0.000
1	10	110609195	0.000	0.000
2	10	11168124	0.625	0.000
2	10	111830308	0.267	0.374
2	10	113814958	0.638	0.000
1	10	115355253	0.000	0.514

2	10	115409272	1.091	0.374
1	10	116513252	0.000	1.357
2	10	116526803	0.400	0.507
1	10	118845653	0.000	0.286
3	10	120224348	2.256	0.000
2	10	12067169	0.356	0.000
2	10	121613197	0.519	0.000
2	10	124588678	0.278	0.159
1	10	131096200	0.000	0.334
1	10	132055519	0.000	0.235
1	10	14501740	0.000	0.286
1	10	19776842	0.000	0.452
2	10	23617158	0.464	0.543
2	10	23958376	0.281	0.472
1	10	24984824	0.000	0.000
2	10	27864409	0.460	0.286
1	10	30158617	0.000	0.483
1	10	33849532	0.000	0.000
2	10	34892061	0.213	0.000
1	10	37826153	0.000	0.505
2	10	4183898	0.406	0.000
1	10	42358812	0.000	0.000
1	10	46311814	0.000	0.000
2	10	47221434	0.370	0.302
2	10	54174376	0.403	0.334
1	10	54568022	0.000	0.280
2	10	56140794	0.267	0.000
2	10	56950504	0.556	0.278
2	10	60147699	0.278	0.047
2	10	62705771	0.747	0.380
2	10	64210270	0.491	0.318
1	10	65525504	0.000	0.000
2	10	67571470	0.403	0.152
2	10	70021310	0.320	1.018
1	10	73104873	0.000	0.475
1	10	75129152	0.000	0.000
2	10	75905930	0.523	0.150
2	10	78393818	0.344	0.235
2	10	83492892	0.544	0.000
1	10	86750662	0.000	0.000
1	10	90700494	0.000	0.575
2	10	9239651	0.325	0.225

1	10	92770872	0.000	0.000
2	10	93187731	0.741	0.724
1	10	93234495	0.000	0.000
2	10	94576898	0.508	0.434
2	10	94950682	0.186	0.193
1	11	103587114	0.000	0.204
2	11	105917523	0.083	0.000
1	11	107071946	0.000	0.579
1	11	11456484	0.000	0.283
1	11	114708783	0.000	0.248
1	11	114958122	0.000	0.290
1	11	117410900	0.000	0.000
2	11	118973448	0.421	0.000
2	11	121731425	0.356	0.066
1	11	122025753	0.000	0.639
1	11	122979808	0.000	0.000
1	11	124291010	0.000	0.251
1	11	132785635	0.000	1.062
1	11	133338345	0.000	0.374
1	11	133761282	0.000	0.620
2	11	17634218	0.640	0.235
2	11	18214790	0.680	0.000
1	11	19063121	0.000	0.000
1	11	20315541	0.000	0.620
1	11	20325176	0.000	0.000
2	11	25629284	0.533	0.000
2	11	29268317	0.348	0.000
2	11	30414572	0.434	0.255
1	11	36738318	0.000	0.181
2	11	40970112	0.271	0.000
2	11	42181489	0.444	0.000
2	11	4222159	0.148	0.000
1	11	42391773	0.000	0.383
2	11	42992114	0.276	0.109
1	11	44879657	0.000	0.414
2	11	49443145	0.457	0.271
2	11	59547928	0.148	0.000
2	11	62907973	0.696	0.310
2	11	6802600	0.600	0.526
2	11	69424352	0.430	0.181
2	11	70172132	0.556	0.000
2	11	71917009	0.406	0.000

2	11	78991748	0.696	0.255
1	11	81625515	0.000	0.121
1	11	82375060	0.000	0.704
1	11	83760647	0.000	0.646
2	11	83859358	0.242	0.000
1	11	8836985	0.000	0.263
1	11	88442713	0.000	0.056
2	11	89364256	0.314	0.056
1	11	91384045	0.000	0.461
2	11	92625315	0.296	0.000
1	11	93116767	0.000	0.334
2	11	93572399	0.583	0.117
2	11	98979001	0.355	0.000
2	11	99123208	0.441	0.000
1	12	100396583	0.000	0.114
2	12	101478301	0.317	0.000
1	12	105124962	0.000	0.247
1	12	109600533	0.000	0.000
2	12	111703138	0.356	0.517
2	12	112201473	0.978	0.000
1	12	113992218	0.000	0.362
1	12	114026760	0.000	0.000
1	12	115283072	0.000	0.557
1	12	126210035	0.000	0.000
1	12	128451756	0.000	0.000
1	12	129158414	0.000	0.101
2	12	129194036	0.260	0.743
2	12	129699976	0.533	0.000
2	12	130201343	0.571	0.000
2	12	132658754	0.387	0.000
1	12	18037838	0.000	0.114
1	12	20532321	0.000	0.334
2	12	27135862	0.333	0.229
1	12	32986809	0.000	0.111
2	12	37544249	0.533	0.000
1	12	38746830	0.000	0.000
2	12	39556427	0.474	0.000
2	12	45801804	0.271	0.399
2	12	47093368	0.444	0.226
2	12	54193118	0.274	0.124
2	12	61096059	0.390	0.310
2	12	6117683	0.107	0.514

1	12	61470230	0.000	0.000
1	12	6307820	0.000	0.000
2	12	67241412	0.303	0.000
1	12	6789295	0.000	0.217
1	12	77504158	0.000	0.145
1	12	78382902	0.000	0.814
1	12	79065377	0.000	0.404
2	12	80465550	0.209	0.041
1	12	80780558	0.000	0.263
1	12	8205043	0.000	0.894
1	12	84484910	0.000	0.000
1	12	87232193	0.000	0.698
1	12	87810848	0.000	0.000
1	12	88071791	0.000	0.000
1	12	90780665	0.000	0.911
1	12	95719552	0.000	0.213
2	12	96021004	0.222	0.931
1	12	96233025	0.000	0.746
2	12	99195605	0.356	0.000
1	13	106932614	0.000	0.217
1	13	109688927	0.000	0.000
2	13	112177050	0.588	0.241
1	13	112351281	0.000	0.000
2	13	19458900	0.200	0.181
2	13	21198801	0.296	0.000
2	13	21198803	0.505	0.277
2	13	22486341	0.667	0.202
2	13	24407230	0.424	0.000
2	13	35458160	0.800	0.106
2	13	44807574	0.290	0.000
2	13	51185115	0.274	0.000
1	13	51338069	0.000	0.181
2	13	54356322	0.222	0.000
2	13	57950093	0.923	0.000
2	13	60127004	0.889	0.181
2	13	61979712	0.208	0.070
1	13	64005202	0.000	0.000
1	13	68771491	0.000	0.000
1	13	81289794	0.000	0.310
2	13	86387551	0.333	0.633
1	13	95505939	0.000	0.000
2	14	81424362	0.598	0.383

1	14	87098315	0.000	0.668
2	14	87211954	0.390	0.000
1	14	94965692	0.000	0.271
1	14	96647102	0.000	0.000
1	14	99920248	0.000	0.000
1	15	25046210	0.000	0.000
2	15	25170693	0.248	0.000
1	15	26017195	0.000	0.000
1	15	45848202	0.000	0.000
1	15	48080087	0.000	0.302
1	15	48928081	0.000	0.000
2	15	56330772	0.406	0.310
1	15	60120205	0.000	0.174
1	15	60352764	0.000	0.281
2	15	60956234	0.327	0.343
2	15	63420915	0.325	0.582
1	15	72502854	0.000	0.691
1	15	77267388	0.000	0.420
1	15	80681168	0.000	0.226
2	15	80950475	0.497	0.045
1	15	83132180	0.000	0.000
2	15	83158851	0.344	0.161
1	15	87983261	0.000	0.000
1	15	95004101	0.000	0.000
2	15	96972773	0.390	0.207
2	15	99025033	0.323	0.326
1	16	1550273	0.000	0.000
1	16	18537225	0.000	0.000
1	16	20424714	0.000	0.000
2	16	22306291	0.600	0.000
1	16	22677228	0.000	0.283
2	16	25681378	0.232	0.290
2	16	3192939	0.311	0.475
2	16	34085953	0.946	0.350
2	16	35232723	0.570	0.000
2	16	47099927	0.325	0.000
2	16	47823047	0.174	0.000
2	16	48066801	0.622	0.592
1	16	50321074	0.000	0.062
2	16	51823583	0.213	0.760
2	16	52276870	0.261	0.000
1	16	5534803	0.000	0.434

1	16	57821035	0.000	0.402
2	16	61886296	0.230	0.000
2	16	64432341	0.232	0.000
2	16	64614439	0.427	0.452
1	16	64699930	0.000	0.000
2	16	64919655	0.174	0.391
2	16	66602481	0.250	0.046
1	16	67105628	0.000	0.465
2	16	67608941	0.081	0.000
1	16	69148663	0.000	0.000
1	16	70802883	0.000	0.395
2	16	71030715	0.254	0.345
2	16	7113874	0.195	0.395
2	16	72362589	0.505	0.434
2	16	7450654	0.857	0.286
1	16	77256710	0.000	0.000
1	16	77493285	0.000	0.362
2	16	77781037	0.370	0.287
1	16	82066056	0.000	0.000
1	16	82874338	0.000	0.229
2	16	83070380	0.667	0.109
3	16	85313584	0.912	0.981
2	16	86284090	0.194	0.334
1	16	9532674	0.000	0.000
1	17	10363095	0.000	0.212
1	17	10978272	0.000	0.241
1	17	14670774	0.000	0.000
2	17	14728731	0.267	0.000
1	17	30755275	0.000	0.000
1	17	31863521	0.000	0.000
2	17	32918315	0.686	0.225
2	17	34590397	0.722	0.307
1	17	34811921	0.000	0.060
2	17	34849723	0.247	0.362
1	17	40381174	0.000	0.000
1	17	41094327	0.000	0.000
2	17	41287213	0.348	0.822
1	17	41488518	0.000	0.347
1	17	41662731	0.000	0.271
1	17	4511744	0.000	0.248
2	17	48780087	0.567	0.543
1	17	4976602	0.000	0.000

1	17	52148573	0.000	0.000
2	17	52202053	0.092	0.000
1	17	59529075	0.000	0.000
1	17	63728030	0.000	0.000
1	17	64448232	0.000	0.000
2	17	72065475	0.566	0.000
2	17	73729002	0.296	0.000
1	17	74236263	0.000	0.000
2	17	78164605	0.333	0.420
2	17	8080028	0.364	0.388
2	17	82853402	0.427	0.322
2	17	9019997	0.310	0.000
1	17	9021059	0.000	0.271
1	18	14791710	0.000	0.000
1	18	15293364	0.000	0.112
2	18	27867692	0.577	0.000
2	18	29896712	0.351	0.000
2	18	50652311	0.242	0.339
2	18	51531692	0.296	0.197
1	18	524695	0.000	0.000
2	18	52758063	0.718	0.395
1	18	56544081	0.000	0.319
2	18	57470379	0.404	0.000
1	18	58043724	0.000	0.452
1	18	61155354	0.000	0.271
1	18	61706959	0.000	0.217
2	18	63454630	0.271	0.000
1	18	63887009	0.000	0.334
1	18	67455918	0.000	0.000
2	18	68909930	0.325	0.345
2	18	70340966	0.496	0.263
1	18	74237595	0.000	0.370
2	18	75784106	0.496	0.724
1	18	75984267	0.000	0.000
1	18	8101586	0.000	0.000
1	18	924915	0.000	0.445
2	18	9601220	0.491	0.566
2	19	10065329	0.571	0.494
3	19	12943750	0.687	0.000
2	19	16234332	0.424	0.193
1	19	19549515	0.000	0.901
1	19	22624713	0.000	0.136

2	19	22883084	0.982	0.000
2	19	22892036	0.649	0.246
1	19	27496424	0.000	0.651
2	19	27753864	0.333	0.672
1	19	28110861	0.000	0.457
2	19	28326785	0.821	0.197
1	19	31656627	0.000	0.062
2	19	3661315	0.410	0.343
1	19	38958665	0.000	0.519
2	19	40522348	0.250	0.452
1	19	41492820	0.000	0.000
2	19	48040435	0.415	0.300
1	19	53377680	0.000	0.524
2	19	7346680	0.325	0.000
1	19	8086471	0.000	0.987
1	20	12995529	0.000	0.414
2	20	13915926	0.323	0.543
1	20	14336990	0.000	0.334
1	20	21629260	0.000	0.383
1	20	22689135	0.000	0.000
2	20	28497717	0.125	0.000
2	20	39766967	0.260	0.434
1	20	39854921	0.000	0.000
2	20	44696207	0.430	0.000
2	20	45737606	0.213	0.293
1	20	48547263	0.000	0.045
1	20	50715766	0.000	0.404
1	20	50895064	0.000	0.000
1	20	52629042	0.000	0.223
2	20	53076039	0.513	0.000
2	20	55145960	0.533	0.271
1	20	64283610	0.000	0.000
2	21	10536357	0.362	0.000
1	21	24993236	0.000	0.079
2	21	25235168	0.667	0.132
2	21	26121394	0.397	0.378
2	21	27842645	0.286	0.000
2	21	28932587	0.260	0.899
2	21	31309695	0.186	0.449
2	21	32999596	0.267	0.000
2	21	35666262	0.496	0.475
1	21	36182754	0.000	0.000

2	21	37443516	0.063	0.157
1	21	40180743	0.000	0.000
1	21	40558302	0.000	0.000
2	21	42078747	0.119	0.259
1	21	46037210	0.000	0.511
1	22	11356944	0.075	0.000
2	22	20582589	1.143	0.000
1	22	32657960	0.000	0.326
2	22	33353441	0.260	0.286
1	22	34793591	0.000	0.000
2	22	37322215	0.360	0.000
1	22	37961231	0.000	0.000
1	22	42966759	0.000	0.170
2	22	44313831	0.545	0.452
2	22	45732457	0.278	0.703
1	22	48568894	0.000	0.302

Note: Results from multidimensional Bayesian Dirichlet Process-based mutation clustering. Columns *CCF_in_Twin_A* and *CCF_in_Twin_B* show for each cluster in each twin the proportion of tumour cells that the cluster represents. CCF, cancer cell fraction.

Supplementary Table 12. Additional candidate cancer genes

**Gene**			
ABL2	*EP300*	*MKL1*	*RAD21*
AFF3	*ERCC2*	*MLL*	*RB1*
APC	*ETV6*	*MLL2*	*RET*
AR	*EZH2*	*MLL3*	*RUNX1*
ARID2	*FBXW7*	*MLL5*	*SDHAF2*
ASXL1	*FLT3*	*MPL*	*SF1*
ATE1	*FOXP1*	*MYC*	*SF3A1*
ATM	*GATA2*	*MYD88*	*SF3B1*
ATRX	*GNAS*	*MYO5A*	*SH2B3*
BCOR	*GNB1*	*NF1*	*SRSF2*
BRAF	*IDH1*	*NOTCH1*	*STAG2*
CALR	*IDH2*	*NPM1*	*STAT3*
CBL	*IKZF1*	*NRAS*	*TET2*
CBLB	*JAK2*	*PABPC1*	*TMEM127*
CDKN2A	*KDM5A*	*PHF6*	*TP53*
CEBPA	*KDM5C*	*PPARG*	*TP63*
CHEK2	*KDM6A*	*PPM1D*	*U2AF1*
CREBBP	*KIT*	*PRPF40B*	*WT1*
CUX1	*KMT2C*	*PTEN*	*ZRSR2*
DNM2	*KRAS*	*PTPN11*	
DNMT3A	*LZTR1*	*PTPRD*	

Note: Additional gene list for putative driver mutation analyses. Curated list based on literature support for genes associated with blood cancer risk of biology.[44-46] Gene names are listed in alphabetical order and provided in italics in accordance with the current HUGO Gene Nomenclature Committee (HGNC) guidelines.[50]

APPENDIX A. Reporting Summary
