# Supplementary material for: In utero origin of myelofibrosis presenting in adult monozygotic twins
Source: Nat Med. 2022 May 30;28(6):1207–11. doi: 10.1038/s41591-022-01793-4 (PMC9205768; doi:10.1038/s41591-022-01793-4)
Supplement: Supplementary file 2 — Reporting Summary [file 41591_2022_1793_MOESM2_ESM.pdf]

nature research

Corresponding author(s): Adam J Mead, David C Wedge

Last updated by author(s): Mar 7, 2022

Reporting Summary

Nature Research wishes to improve the reproducibility of the work that we publish. This form provides structure for consistency and transparency in reporting. For further information on Nature Research policies, see our Editorial Policies and the Editorial Policy Checklist.

Statistics

For all statistical analyses, confirm that the following items are present in the figure legend, table legend, main text, or Methods section.

n/a	Confirmed	
☐	☒	The exact sample size (*n*) for each experimental group/condition, given as a discrete number and unit of measurement
☐	☒	A statement on whether measurements were taken from distinct samples or whether the same sample was measured repeatedly
☐	☒	The statistical test(s) used AND whether they are one- or two-sided<br>*Only common tests should be described solely by name; describe more complex techniques in the Methods section.*
☒	☐	A description of all covariates tested
☒	☐	A description of any assumptions or corrections, such as tests of normality and adjustment for multiple comparisons
☐	☒	A full description of the statistical parameters including central tendency (e.g. means) or other basic estimates (e.g. regression coefficient) AND variation (e.g. standard deviation) or associated estimates of uncertainty (e.g. confidence intervals)
☐	☒	For null hypothesis testing, the test statistic (e.g. *F*, *t*, *r*) with confidence intervals, effect sizes, degrees of freedom and *P* value noted<br>*Give P values as exact values whenever suitable.*
☐	☒	For Bayesian analysis, information on the choice of priors and Markov chain Monte Carlo settings
☐	☒	For hierarchical and complex designs, identification of the appropriate level for tests and full reporting of outcomes
☒	☐	Estimates of effect sizes (e.g. Cohen's *d*, Pearson's *r*), indicating how they were calculated

Our web collection on statistics for biologists contains articles on many of the points above.

Software and code

Policy information about availability of computer code

Data collection

The clinical, immunophenotyping, and genetic (including genome sequencing) data were acquired as outlined in the Methods section.

Data analysis

Bioinformatic analysis:

Open source code was used in all analyses described in this study. In each case, the publication and relevant GitHub repository are fully referenced in the Methods. This is especially relevant to:

• Bayesian Dirichlet processing as detailed in the Methods, using DPClust v2.2.2 implemented in R 3.4.0 (1,2)
• Bayesian probabilistic model for estimation of MPN time of origin (3)
• Extraction of mutational signatures using the SigProfilerMatrixGenerator (v1.1.23) and SigProfilerExtractor (v1.1.0) packages implemented in Python 3.8.3 (4,5,6)
• Methods for analysis of structural variation including Lumpy (v0.2.13), Manta (v1.6.0) and SVTyper (v0.7.1) (7,8,9)

1. https://github.com/Wedge-lab/dpclust
2. Bolli et al. Nat Comms. (2014)
3. https://github.com/Wedge-lab/InUtero_MPN
4. Bergstrom et al. BMC Genomics (2019)
5. https://github.com/AlexandrovLab/SigProfilerMatrixGenerator
6. https://github.com/AlexandrovLab/SigProfilerExtractor
7. https://github.com/Illumina/manta
8. https://github.com/arq5x/lumpy-sv
9. https://github.com/hall-lab/svtyper

Microscopy:
INFINITY ANALYZE software, release 6.5 (Lumenera Corporation, Ottawa, CA)
NDP.view2 viewer software, version 2.9.25 (Hamamatsu Photonics K.K., Hamamatsu, JP)
Adobe Photoshop version 21.2 (Adobe Inc., San Jose, US-CA)

Flow cytometry and cell sorting:
FACSDIVA™ software v8.0.1. (Becton Dickinson and Company, Franklin Lakes, US-NJ)
FlowJo software v10.7 (Becton Dickinson and Company, Franklin Lakes, US-NJ)
R studio version 3.6.3

Polymerase chain reaction (PCR):
NCBI Primer-BLAST® (National Library of Medicine, Bethesda, US-MD)
SnapGene® Viewer v5.3 (GSL Biotech LLC, Chicago, US-IL)
QX Manager Software, Standard Edition, v1.2 (Bio-Rad Laboratories, Inc, Hercules, US-CA)

Electrophoresis:
Agilent Fragment Analyzer™ version 2.2.1 (Agilent Technologies, Inc. Santa Clara, US-CA)
Agilent TapeStation 2200 A.0202 SR1 software (Agilent Technologies, Inc. Santa Clara, US-CA)

For manuscripts utilizing custom algorithms or software that are central to the research but not yet described in published literature, software must be made available to editors and reviewers. We strongly encourage code deposition in a community repository (e.g. GitHub). See the Nature Research guidelines for submitting code & software for further information.

Data

Policy information about availability of data

All manuscripts must include a data availability statement. This statement should provide the following information, where applicable:

- Accession codes, unique identifiers, or web links for publicly available datasets
- A list of figures that have associated raw data
- A description of any restrictions on data availability

Re twin study:
Please see 'Data availability' and 'Code availability' within the Methods section of the manuscript. As described:
Raw whole genome raw sequencing data have been deposited at the European Genome-phenome Archive (EGA), which is hosted by the European Bioinformatics Institute (EBI) and the Centre for Genomic Regulation (CRG), under accession number EGAS00001005744 (https://wwwdev.ebi.ac.uk/ega/studies/EGAS00001005744).
Data on all somatic SNVs, indels, and structural rearrangements for both individuals are available in Extended Data Tables 1 and 2. Source data for the Bayesian timing model is described in Extended Data Table 5. COSMIC Mutational Signatures v3.1 and Gene Curation data can be accessed at https://cancer.sanger.ac.uk/cosmic/. Source data described in Gerstung et al. (2020) and used in this study can be accessed via the ICGC Data Portal https://dcc.icgc.org/pcawg.
Patients' clinical details are described in the Main Text ("Clinical Findings" section).

Re JAK2V617F-mutant MPN cohort:
All patients' clinical details are described in the Main Text ("Neonatal Blood Spot Analysis in JAK2V617F-mutant MPN") plus Extended Data Table 7.

Field-specific reporting

Please select the one below that is the best fit for your research. If you are not sure, read the appropriate sections before making your selection.

☒ Life sciences ☐ Behavioural & social sciences ☐ Ecological, evolutionary & environmental sciences

For a reference copy of the document with all sections, see nature.com/documents/nr-reporting-summary-flat.pdf

Life sciences study design

All studies must disclose on these points even when the disclosure is negative.

Sample size	We did not perform sample size or formal power calculations as this was not relevant in the context of our analysis. Sample size was determined based on the total number of available samples
Data exclusions	No data were excluded in this study.
Replication	Re twin study, no replication was done. Rigor of the study was maintained by orthogonal validation of mutations as described in the Methods. Sanger DNA sequencing was applied for the validation of somatic variants called by next generation whole genome sequencing and tested on single-cell colonies (twin A) and bulk samples (twin B).<br><br>Re dried blood spot analysis, results were independently validated using a nested PCR assay.
Randomization	Re twin study: Randomization was not relevant to this study. A twin pair were recruited based on the observation of CALR mutation positive MPN. No other selection criteria were applied. Recruitment was therefore non-random based on this criterion only.<br><br>Re dried blood spot analysis: JAK2-mutant MPN cohort included all MPN patients followed up in OUH NHS Trust Haematology with stored Guthrie cards available. Randomization was not relevant in this part of the study either.

Blinding	Blinding was not relevant as this was not an intervention study.

Reporting for specific materials, systems and methods

We require information from authors about some types of materials, experimental systems and methods used in many studies. Here, indicate whether each material, system or method listed is relevant to your study. If you are not sure if a list item applies to your research, read the appropriate section before selecting a response.

Materials & experimental systems

n/a	Involved in the study
☐	☒ Antibodies
☐	☒ Eukaryotic cell lines
☒	☐ Palaeontology and archaeology
☒	☐ Animals and other organisms
☐	☒ Human research participants
☒	☐ Clinical data
☒	☐ Dual use research of concern

Methods

n/a	Involved in the study
☒	☐ ChIP-seq
☐	☒ Flow cytometry
☒	☐ MRI-based neuroimaging

Antibodies

Antibodies used

All antibodies used are described in Supplementary Table 1.

Re Granulocyte panel we used:
CD3 SK7 FITC, BD Biosciences, Catalog No.: 345763, Dilution 5 / 100
CD34 4HI1 APC-eFluor® 780, eBioscience™, Catalog No.: 47-0349-42, Dilution 2 / 100
CD71 M-A712 Alexa Fluor® 700, BD Biosciences, Catalog No.: 563769, Dilution 5 / 100
CD19 HIB19 V450, BD Biosciences, Catalog No.: 560354, Dilution 5 / 100
7AAD (7-Aminoactinomycin D), Cayman Chemical, Catalog No.: 11397, Dilution 1 / 100 (previously diluted 1:200 from the 5mg/ml stock solution)
CD11b ICRF44 APC, eBioscience™, Catalog No.: 17-0118-42, Dilution 5 / 100
CD14 61D3 APC, eBioscience™, Catalog No.: 17-0149-42, Dilution 5 / 100
CD33 WM53 PE, BioLegend, Catalog No.: 303404, Dilution 5 / 100

Re T-cell panel:
CD3 SK7 FITC, BD Biosciences, Catalog No.: 345763, Dilution 5 / 100
CD4 SK3 APC, BD Pharmingen™, Catalog No.: 565994, Dilution 5 / 100
DAPI (4',6-Diamidino-2-Phenylindole, Dilactate), Invitrogen™, Catalog No.: D3571, Dilution 1 / 100 (previously diluted 1:100 from the 5mg/ml stock solution)
CD19 HIB19 APC-Cyanine7, BioLegend, Catalog No.: 302218, Dilution 5 / 100
CD11b ICRF44 PE-Cyanine5, BioLegend, Catalog No.: 301308, Dilution 5 / 100
CD14 61D3 PE-Cyanine5, eBioscience™, Catalog No.: 15-0149-42, Dilution 5 / 100

Re HSPC panel:
CD34 4HI1 APC-eFluor® 780, eBioscience™, Catalog No.: 47-0349-42, Dilution 0.66 / 100
CD38 HIT2 PE-Texas Red®, Invitrogen™, Catalog No.: MHCD3817, Dilution 4.66 / 100
CD45RA HI100 PE, BioLegend, Catalog No.: 304108, Dilution 0.66 / 100
CD90 5E10 Brilliant Violet 421™, BioLegend, Catalog No.: 328122, Dilution 3.33 / 100
CD123 6H6 PE-Cyanine7, BioLegend, Catalog No.: 306010, Dilution 1.66 / 100
7AAD (7-Aminoactinomycin D), Cayman Chemical, Catalog No.: 11397, Dilution 1 / 100 (previously diluted 1:200 from the 5mg/ml stock solution)
Lineage Mix in a dilution ratio of 21.66 / 100:
CD8a RPA-T8 FITC, BioLegend, Catalog No.: 301006, Dilution 1 / 100 of the Lineage Mix
CD10 HI10a FITC, BioLegend, Catalog No.: 312208, Dilution 3.33 / 100 of the Lineage Mix
CD20 2H7 FITC, BioLegend, Catalog No.: 302304, Dilution 0.66 / 100 of the Lineage Mix
CD66b G10F5 FITC BioLegend, Catalog No.: 305104, Dilution 6.66 / 100 of the Lineage Mix
CD127 eBioRDR5 FITC eBioscience™, Catalog No.: 11-1278-52, Dilution 3.33 / 100 of the Lineage Mix
Human Hematopoietic Lineage Cocktail (CD2,CD3,CD14, CD16,CD56,CD235a) RPA-2.10, OKT3, 61D3, CB16, HIB19, TULY56, HIR2 FITC eBioscience™, Catalog No.: 22-7778-72, Dilution 6.66 / 100 of the Lineage Mix

BD Biosciences / BD Pharmingen, Becton, Dickinson and Company, Franklin Lakes, US-NJ; eBiosciences / Invitrogen, Thermo Fisher Scientific Inc., Waltham, US-MA; Cayman Chemical, Cayman Chemical Company, Ann Arbor, US-MI; BioLegend, San Diego, US-CA

Validation

Antibodies were purchased from BioLegend, Invitrogen, eBioscience, BD Biosciences, BD Pharmingen, and Cayman Chemical. Validation information is available from the manufacturers who provide references on their websites for the catalogue number listed in Supplementary Table 1. See https://www.biolegend.com/ for Biolegend, https://www.thermofisher.com/invitrogen for Invitrogen, https://www.bdbiosciences.com/ for BD Biosciences, https://www.thermofisher.com/ebioscience for eBioscience, https://www.bdbiosciences.com/ for BD Pharmingen, and https://www.caymanchem.com for Cayman Chemical.
Antibody validation and overall quality performance of each panel was done with use of Single Stain, fluorescence Minus One, and

unstained controls.

Eukaryotic cell lines

Policy information about cell lines

Cell line source(s)	Jurkat cell line (CVCL_0065) was used as the CALRdel52bp-negative control.<br>TF-1 (CVCL_0559) and HEL (CVCL_0001) cell lines were used as JAK2V617F positive and negative controls, respectively.<br>All cell lines were purchased from ATCC (American Type Culture Collection, Manassas, US-VA) (ATCC catalogue numbers: TIB-152, CRL-2003, and TIB-180 for Jurkat, TF-1 and HEL, respectively) by Haematopoietic Stem Cell Biology Laboratory, MRC Molecular Haematology Unit, MRC Weatherall Institute of Molecular Medicine, University of Oxford.
Authentication	None of the cell lines were authenticated
Mycoplasma contamination	All cell lines (Jurkat, TF-1, and HEL cells) were tested negative for Mycoplasma contamination
Commonly misidentified lines<br>(See ICLAC register)	No commonly misidentified cell lines were used in the study.

Human research participants

Policy information about studies involving human research participants

Population characteristics	Re the twin study, the clinical characteristics of the twins are outlined in detail in the Main Text (see "Clinical Findings").<br><br>Re dried blood spot analysis, the clinical characteristics of the patients included in the study are provided in the Main Text (see "Neonatal Blood Spot Analysis in JAK2V617F-mutant MPN" section) and Extended Data Table 7, including age and JAK2V617F variant allele frequency at MPN diagnosis.
Recruitment	Re the twin study: A twin pair were recruited based on the observation of CALR mutation positive MPN. No other selection criteria were applied. Due to the nature of the study design (twin study) no selection bias is relevant. The type of studies performed ensure no information bias or confounding is relevant / present either. Regarding twin A, samples were obtained from the centre he is followed up in India (Christian Medical College, Vellore). Regarding twin B and the control CALR mutation positive myelofibrosis patient, written informed consent was taken for additional genetic analysis carried out (The INForMeD Study, National Health Service (NHS) Health Research Authority, London - Brent Research Ethics Committee, REC Reference 16/LO/1376, Date 26 July 2016).<br><br>Re dried blood spot analysis: JAK2-mutant MPN cohort included all MPN patients followed up in OUH NHS Trust Haematology with stored Guthrie cards available. Inclusiveness of the group studied and the type of the analysis (molecular analysis of their dried blood spot sample for presence of the MPN driver mutation) ensure that no biases that are typical for retrospective studies (such as selection bias, misclassification bias, observer bias, recall bias and reporting bias) are present in this study. Samples were collected under The INForMeD Study with specific ethics approvals regarding Guthrie card retrieval and dried blood spot mutational analysis (ANNB_NBS_027 study, Public Health England Antenatal and Newborn (ANNB) screening research advisory committee, Date 13 March 2020).
Ethics oversight	All procedures followed in the present study were performed in accordance with the ethical standards of the current revision of the Declaration of Helsinki. All patients provided written informed consent. Regarding twin A, samples were obtained from the centre he is followed up in India (Christian Medical College, Vellore) for diagnostic purposes. Other patients were enrolled to The INForMeD Study (Version 1.0. Date 26 July 2016, REC Reference 16/LO/1376). All research analyses were conducted according to The INForMeD Study (Version 1.0. Date 26 July 2016, REC Reference 16/LO/1376) and ANNB_NBS_027 study, Public Health England Antenatal and Newborn (ANNB) screening research advisory committee, Date 13 March 2020.

Note that full information on the approval of the study protocol must also be provided in the manuscript.

Flow Cytometry

Plots

Confirm that:

- [x] The axis labels state the marker and fluorochrome used (e.g. CD4-FITC).
- [x] The axis scales are clearly visible. Include numbers along axes only for bottom left plot of group (a 'group' is an analysis of identical markers).
- [x] All plots are contour plots with outliers or pseudocolor plots.
- [x] A numerical value for number of cells or percentage (with statistics) is provided.

Methodology

Sample preparation	Relevant details are provided in the Methods section (Cell isolation, Flow cytometry and cell sorting, and Single-cell cloning assay paragraphs, plus Supplementary Table 1)

Instrument	BD FACSAria Fusion Cell Sorter (Becton Dickinson and Company, Franklin Lakes, US-NJ)
Software	FACSDIVA software v8.0.1; FlowJo software v10.7
Cell population abundance	Index-sorting analysis details are provided in the Methods Section, and Figure 2 of the main text.
Gating strategy	Relevant details are provided in the Methods section (Cell isolation, Flow cytometry and cell sorting, and Single-cell cloning assay paragraphs, plus Supplementary Figure 1 and Table 1)

☒ Tick this box to confirm that a figure exemplifying the gating strategy is provided in the Supplementary Information.
